# De novo lipogenesis is elicited dramatically in human hepatocellular carcinoma especially in hepatitis C virus‐induced hepatocellular carcinoma

**DOI:** 10.1002/mco2.15

**Published:** 2020-07-09

**Authors:** Shaojian Li, Ruonan Liu, Qinling Pan, Genshu Wang, Daorou Cheng, Jie Yang, Hui Chen, Geyang Xu

**Affiliations:** ^1^ Department of Physiology School of Medicine Jinan University Guangzhou China; ^2^ Department of Hepatic Surgery and Liver Transplantation Center The Third Affiliated Hospital of Sun Yat‐sen University Guangzhou China; ^3^ Hepatobiliary Pancreaticosplenic Surgery Shunde Hospital of Southern Medical University Foshan China; ^4^ Guangdong Key Laboratory of Liver Disease Research The Third Affiliated Hospital of Sun Yat‐sen University Guangzhou China

**Keywords:** de novo lipogenesis, HCV‐HCC, human hepatocellular carcinoma

## Abstract

Hepatocellular carcinoma (HCC) is the third leading cause of cancer deaths worldwide. Abnormal de novo lipogenesis is reported to be involved in hepatocarcinogenesis. In current study, de novo lipogenesis and its association with patient survival rate were investigated in human HCC samples induced by hepatitis B virus (HBV), hepatitis C virus (HCV), or nonviral factors. Hepatic mRNA and protein levels of lipogenic transcription factors and lipid synthesis enzymes were examined by realtime‐PCR (RT‐PCR) and western blot. Association of gene expression and patient survival was analyzed using The Cancer Genome Atlas (TCGA) data. Lipogenic pathway regulators such as AKT2, SREBP1c, PPARγ, and lipogenic enzymes such as ACC and FAS were increased in human HCC when compared with control livers. Notably, a more robust increase in de novo lipogenesis was observed in HCV‐HCC when compared to HBV‐HCC and nonviral HCC. High FAS and ACC expression correlated with poor overall survival (OS) in HCV‐HCC. High expression of lipogenesis gene panel significantly correlated with poor OS in HCV‐HCC, but not in HBV‐HCC or nonviral HCC. In sum, de novo lipogenesis is stimulated dramatically in human HCC especially in HCV‐HCC.

## Graphical abstract

De novo lipogenesis is elicited dramatically in human hepatocellular carcinoma (HCC) especially in hepatitis C virus‐induced HCC when compared to control livers.

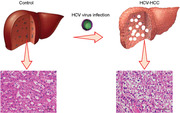

**Abbreviations**
ACC: acetyl‐CoA carboxylase
ChREBP: carbohydrate response element binding protein
FAS: fatty acid synthase
HBV: hepatitis B virus
HCC: hepatocellular carcinoma
HCV: hepatitis C virus
H&E: hematoxylin and eosin
OS: overall survival
PPARγ: peroxisome proliferator‐activated receptor γ
SCD1: stearoyl‐CoA desaturase 1
SREBP1c: sterol response element‐binding protein 1c

## 1 INTRODUCTION

Liver cancer is the third leading cause of cancer death worldwide and one of the cancers with a still increasing incidence rate.^1^ Hepatocellular carcinoma (HCC), the most common type of primary liver malignancy, accounts for 90% of liver cancers. Chronic infections with hepatitis B viruses (HBVs) or hepatitis C viruses (HCVs) are the main causes of HCC, which attribute to at least 75% of all HCC cases.^1^ In most cases, hepatitis virus‐associated HCC develops progressively from chronic liver diseases, including fatty liver and cirrhosis.^1^, ^2^ Multiple nonviral factors have also been implicated in the development of HCC. Nonalcoholic fatty liver disease, obesity, diabetes, and alcohol have consistently been shown to dramatically increase the risk of HCC.^3^, ^4^, ^5^ Although surgical resection and liver transplantation are considered curative in early stage HCC, the 5‐year recurrence rate is as high as 70%.^6^ Unfortunately, the majority of HCC patients are diagnosed at advanced stage, when treatment options for HCC are limited and generally ineffective. Thus, the investigation of potential mechanisms leading to HCC development and progression is crucial to identifying new targets for its early diagnosis, chemoprevention, and treatment. HCV infection is one of the major risk factors for both chronic liver disease and the increasing HCC incidence.^1^ HCV‐induced hepatocarcinogenesis involves multiple mechanisms affected by viral proteins including chronic inflammation, oxidative stress, hypoxia, and so forth.^7^ Hepatic steatosis is prevalent in HCV infection.^8^ HCV hijacks host lipid metabolism to support efficient virus replication, resulting in decreased lipid catabolism and activated lipogenic pathways.^8^, ^9^ Hepatic steatosis is associated with increased risk of HCC in patients infected by HCV,^10^ suggesting that change of lipid metabolism may contribute to carcinogenesis.

Metabolic reprogramming is now recognized as one of the hallmarks of cancer.^11^ Aberrant fatty acid metabolism has been observed in various cancer types.^12^ In particular, de novo lipogenesis is often seen to be upregulated in solid tumors, and tumor cells become less dependent on exogenous fatty acids for growth.^13^ In the fatty acid biosynthetic pathway, the enzymes such as acetyl‐CoA carboxylase (ACC), fatty acid synthase (FAS), and stearoyl‐CoA desaturase 1 (SCD1) are of particular importance.^14^ ACC and FAS catalyze the fatty acid biosynthesis from acetyl‐CoA and SCD1 introduces double bonds to fatty acids to form unsaturated fatty acids.^14^ These enzymes are known to be coordinately induced in lipogenesis by lipogenic transcription factors sterol response element‐binding protein 1c (SREBP1c) and peroxisome proliferator‐activated receptor γ (PPARγ).^15^, ^16^ Blocking the fatty acid synthesis by targeting these enzymes or transcription factors inhibits cell proliferation and induces cell death of various cancer cells in vitro and in vivo.^13^ Therefore, inhibitors for lipogenic enzymes and transcription factors are considered potential anticancer therapies.^13^

In the present study, we examined the key lipogenic transcription factors and enzymes in human control livers, cirrhotic livers, HBV‐HCC, HCV‐HCC, and nonviral HCC. We also analyzed correlation between lipogenic pathway activation and overall survival (OS) of HCC patients with different etiologies. Our study enhances the understanding on lipid metabolism in human HCC associated with different etiological factors. Potential use of lipogenic molecules as drug targets and prognosis markers may be etiology dependent.

## 2 MATERIALS AND METHODS

### 2.1 Antibodies

AKT2 rabbit mAb, PPARγ rabbit mAb, Acetyl‐CoA carboxylase rabbit mAb, and β‐actin mouse mAb were purchased from Cell Signaling Technology (Beverly, MA). Rabbit anti‐FAS and SREBP1 mouse mAb were from Abcam Inc (Cambridge, MA). Horseradish peroxidase‐conjugated, donkey anti‐Rabbit IgG and donkey anti‐Mouse IgG were purchased from Jackson ImmunoResearch (West Grove, PA).

### 2.2 Patients and tissue specimens

Patients with liver cirrhosis, HBV‐HCC, HCV‐HCC, or nonviral HCC (six subjects for each group) and six age‐matched control subjects were enrolled in the study. The control specimens were obtained from liver transplantation donors. All the control specimens were histologically normal. Cirrhotic samples and cancer samples were obtained from patients who underwent either tumor resection or transplantation. All participants did not take any antihyperlipidemic drugs for at least 1 week before biopsy collection. Individuals were excluded if they were treated with drugs known to affect hepatic steatosis. Anthropometric data are provided in Tables S1 and S2. Participation in this study was voluntary. Written informed consent was obtained from each participants. The guidelines of the Declaration of Helsinki of the World Medical Association were followed. All protocols were approved by the Research Ethics Committee of the Third Affiliated Hospital of Sun Yat‐Sen University. Tissue samples were extracted for protein and RNA or for HE staining, respectively.

### 2.3 Histological analysis

Human liver samples were fixed in 4% paraformaldehyde, paraffin‐embedded, cut into sections. H&E staining was performed according to standard procedures.

### 2.4 Quantitative real‐time PCR

Total RNAs from livers were isolated using the Trizol reagent. RNAs were reverse‐transcribed into cDNAs using Takara‐PrimeScriptTM RT reagent Kit. SYBR Green‐based real‐time PCR was performed as described previously.^17^ PCR primer sequences are listed in Table S3.

### 2.5 Western blot analysis

Western blot was performed as described previously.^17^ Primary and secondary antibodies are shown in Section 2.1.

### 2.6 TCGA data analysis

TCGA‐LIHC RNA sequencing data and patient information were obtained from National Cancer Institute GDC data portal (https://portal.gdc.cancer.gov). Only HCC sample data were used for analysis. Patients with history of hepatitis B or hepatitis C, or positive for HBV or HCV serologic test, are considered viral HCC patients. Patients with only hepatitis B history or positive for HBV and patients with only hepatitis C history or positive for HCV serologic test were considered HBV‐HCC and HCV‐HCC, respectively. Patients without history of hepatitis virus or positivity for serologic test are considered nonviral HCC patients. We evaluated the correlations between mRNA expression, which was represented by fragments per kilobase per million mapped reads (FPKM) value, of lipogenesis pathway genes and OS rate of HCC patients. For single variable analysis, the cutoff for high and low expression was determined by the optimal cut‐point. For lipogenesis gene panel analysis, patients with more than three out of four genes expression above the medians were considered lipogenesis high, otherwise, lipogenesis low. The clinicopathological information and FPKM value of lipogenic genes for patient samples are provided in Table S4.

### 2.7 Statistical analysis

All data are expressed as mean values ± SEM. Statistical differences were evaluated by one‐way ANOVA and Newman‐Student‐Keuls test. Differences were significant with *P*‐values less than .05. Correlations between gene expression and patient survival were presented as Kaplan‐Meier survival plots and a log‐rank test was used to assess the significance.

## 3 RESULTS

### 3.1 Histological characteristics of patients with fatty liver and HCV‐HCC

**FIGURE 1:**
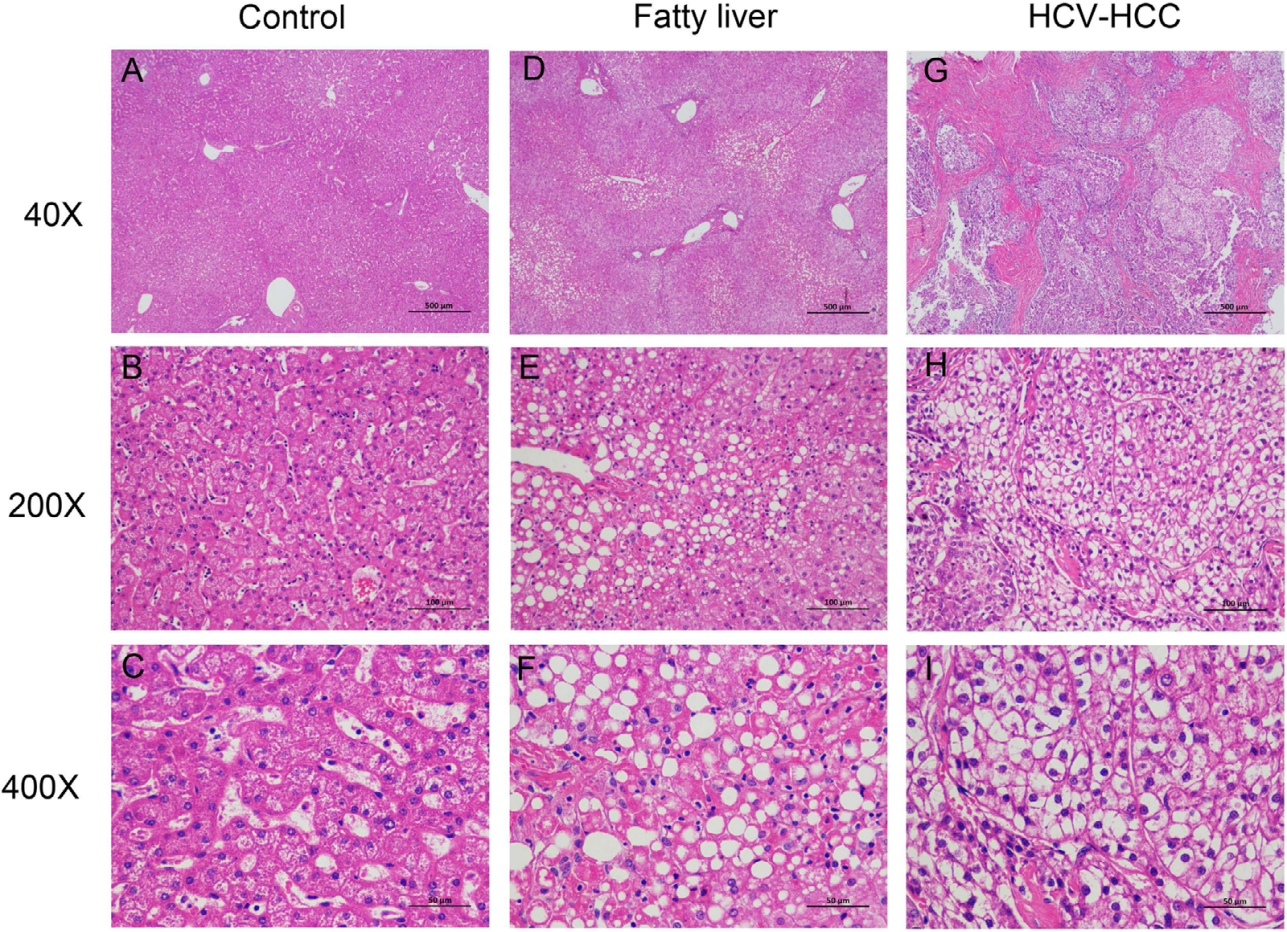
H&E staining analysis of hepatic steatosis in human control livers, fatty livers, and HCV‐HCC tissues. Representative H&E staining from human control livers (A‐C), fatty livers (D‐F), and HCV‐HCC (G‐I). Images were taken with magnification of 40×, 200×, and 400×

We performed H&E staining on human control liver, fatty liver, and HCV‐HCC. Compared to control liver tissue (Figure 1A‐C), nonalcoholic fatty liver showed increased lipid accumulation (Figure 1D‐F). Some hepatocytes had large droplets of fat that pushed the nucleus aside, which is a typical macrovesicular steatosis morphology. In HCV‐HCC, strikingly strong lipid accumulation was seen in the cytoplasm (Figure 1G‐I). In contrast to the large lipid droplet in fatty liver, lipid droplets in HCV‐HCC emerged as smaller droplets and in more diffused pattern.

### 3.2 Coordinated induction of lipogenic proteins in human HBV‐HCC, HCV‐HCC, and nonviral HCC

**FIGURE 2:**
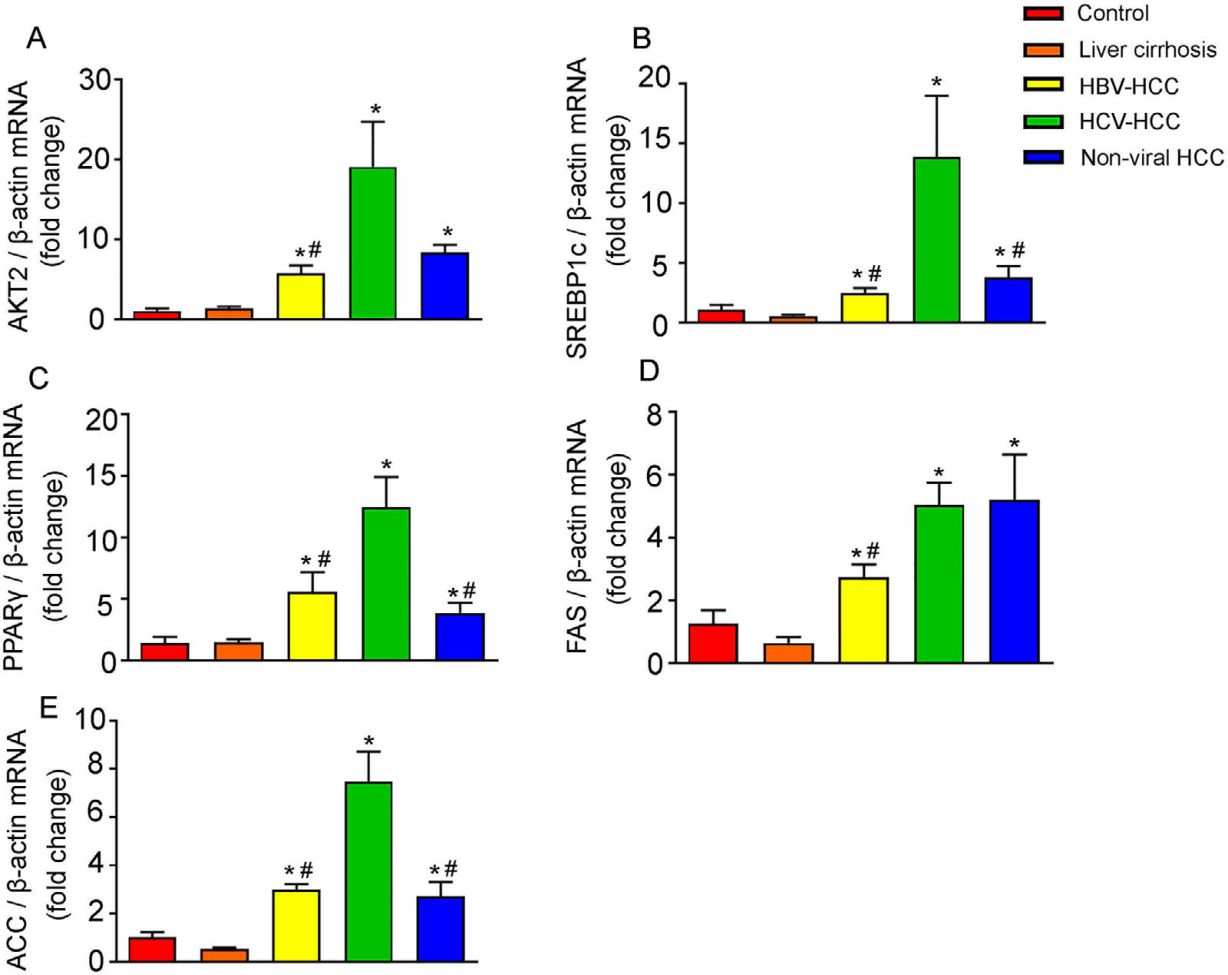
Quantitative reverse transcription polymerase chain reaction analysis of lipogenic proteins in human control livers, cirrhotic livers, and HCC tissues. Results of quantitative PCR analysis of AKT2 mRNA (A), SREBP1c mRNA (B), PPARγ mRNA (C), FAS mRNA (D), and ACC mRNA (E) in control human livers, cirrhotic livers, HBV‐HCC, HCV‐HCC, and nonviral HCC tissues are expressed as fold change over control livers using β‐actin as internal control. n = 6, ^*^ *P* < .05 versus control human livers; ^#^ *P* < .05 versus HCV‐HCC

**FIGURE 3:**
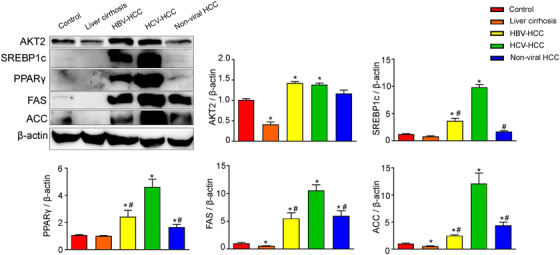
Western blot analysis of lipogenic proteins in human control livers, cirrhotic livers, and HCC tissues. Representative western blot of control human livers, cirrhotic livers, HBV‐HCC, HCV‐HCC, and nonviral HCC tissues. AKT2, SREBP1c, PPARγ, FAS, and ACC were blotted as described in Section 2. β‐Actin was used as loading controls. Quantification of image analysis of hepatic lipogenic proteins expression is expressed as mean values ± SEM. n = 6, ^*^ *P* < .05 versus control human livers; ^#^ *P* < .05 versus HCV‐HCC

Given the lipid accumulation in HCV‐HCC, we further investigated the expression of hepatic lipogenic enzymes and those of their upstream lipogenic transcription factors. As shown by quantitative RT‐PCR results, mRNAs of enzymes required for de novo lipogenesis ACC and FAS as well as their upstream transcription factors SREBP1c, PPARγ, and carbohydrate response element binding protein (ChREBP) were significantly upregulated in human HCC compared to control liver (Figures 2B‐E and S1). AKT2, which is essential for the induction of hepatic SREBP1c and lipogenesis,^18^ was also increased in HCC tissues (Figure 2A). Notably, ACC, SREBP1c, and PPARγ were more dramatically upregulated in HCV‐HCC compared to HBV‐HCC and nonviral HCC (Figure 2). In concordance with the RT‐PCR results, western blot showed upregulation of these proteins in HCC. Upregulation of FAS, ACC, PPARγ, and SREBP1c was more significant in HCV‐HCC than HCC associated with other etiologies (Figure 3). Expression of lipogenic genes was slightly downregulated or not altered in cirrhotic livers compared with control liver (Figures 2 and 3). Overexpression of lipogenic enzymes and their upstream regulators suggested robust activation of de novo lipogenesis in HCC, especially HCV‐associated HCC.

### 3.3 Lipogenesis is correlated with patient survival in viral HCC

**FIGURE 4:**
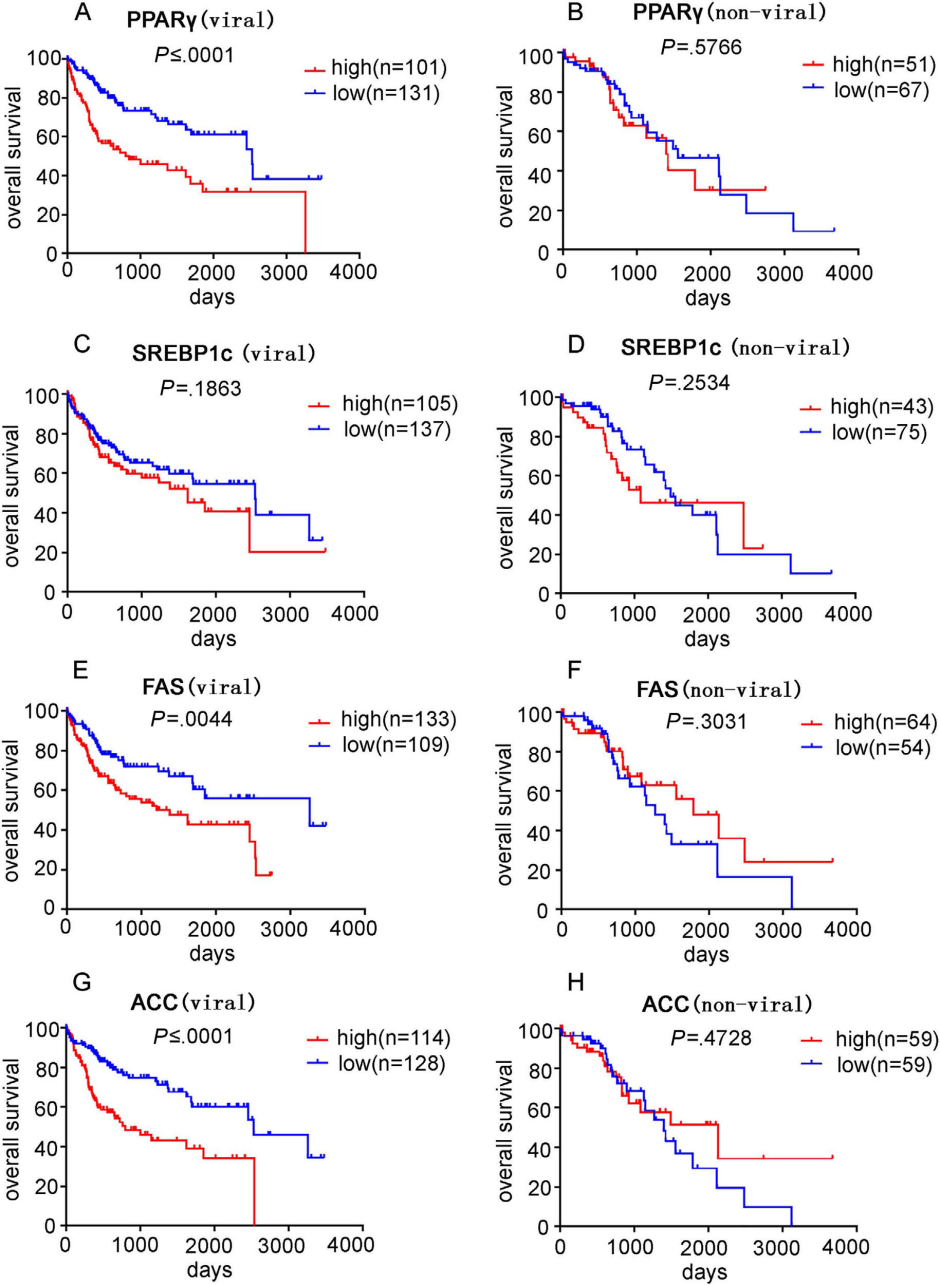
Analysis of the association between lipogenesis and survival outcome in HCC patients. The Kaplan‐Meier statistical analysis was used to analyze the survival curves of HCC patients with different expression levels of PPARγ, SREBP1c, FAS, and ACC. Survival curve of PPARγ in viral HCC (A) and nonviral HCC (B) patients; survival curve of SREBP1c in viral HCC (C) and nonviral HCC (D) patients; survival curve of FAS in viral HCC (E) and nonviral HCC (F) patients; survival curve of ACC in viral HCC (G) and nonviral HCC (H) patients

Because we observed more significant alterations of lipogenesis gene expression in HCV‐HCC compared to HBV and nonviral HCC, we further examined whether these changes had correlation with survival of patients with different etiologies. High expression of PPARγ, FAS, and ACC was significantly associated with poor OS in viral HCC (Figures 4A, 4E, and 4G). High SREBP1c also showed a trend of association with poor survival, although not statistically significant (Figure 4C). In contrast, none of the lipogenesis genes showed correlation with OS rate in nonviral HCC patients (Figures 4B, 4D, 4F, and 4H).

**FIGURE 5:**
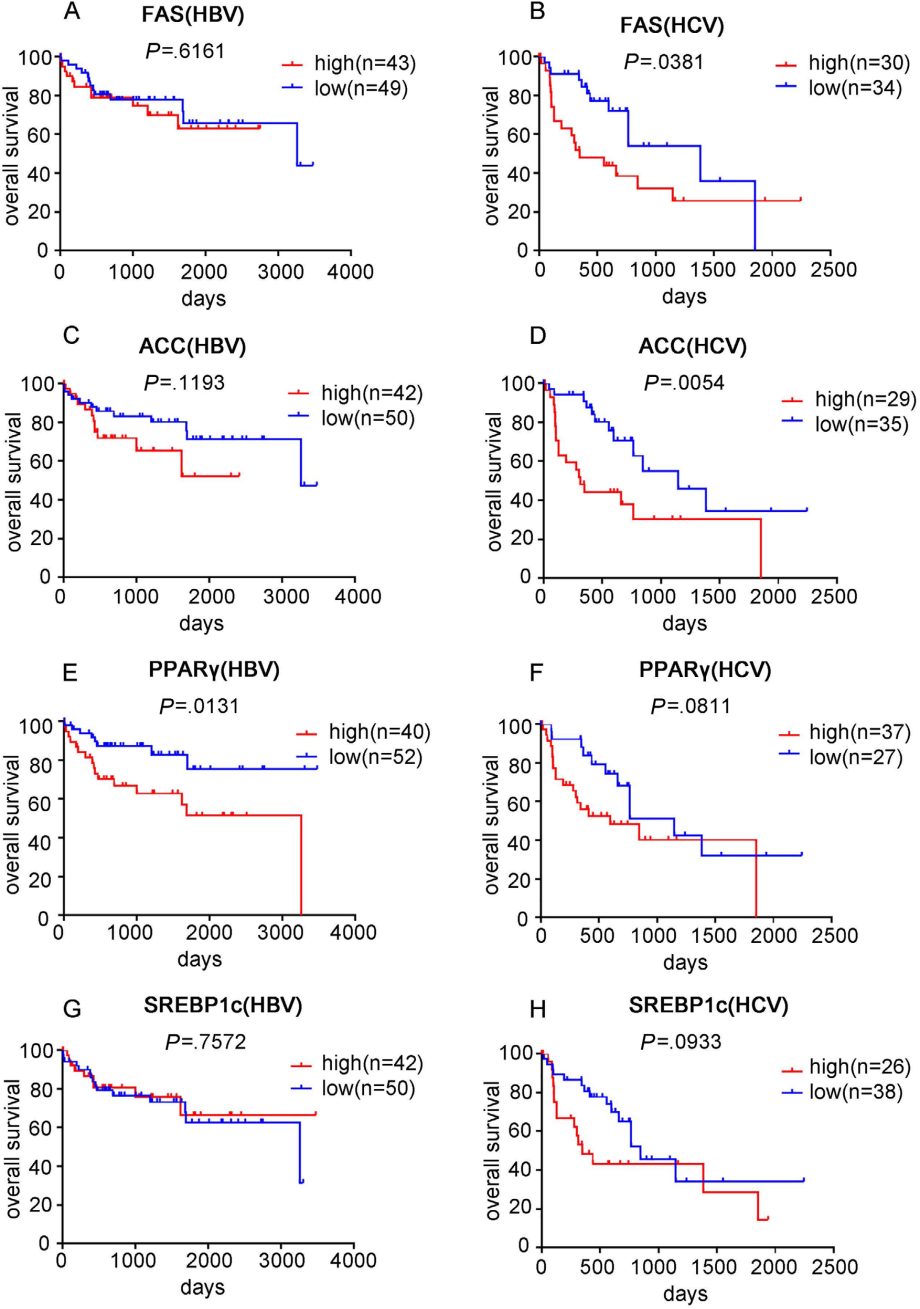
Analysis of the association between lipogenesis and survival outcome in HBV‐HCC and HCV‐HCC patients. The Kaplan‐Meier statistical analysis was used to analyze the survival curves of HCC patients with different expression levels of FAS, ACC, PPARγ, and SREBP1c. Survival curve of FAS in HBV‐HCC (A) and HCV‐HCC (B) patients; survival curve of ACC in HBV‐HCC (C) and HCV‐HCC (D) patients; survival curve of PPARγ in HBV‐HCC (E) and HCV‐HCC (F) patients; survival curve of SREBP1c in HBV‐HCC (G) and HCV‐HCC (H) patients

Because the viral HCC group consisted of patients with HBV, HCV, or both HBV and HCV infection, we further extracted the cases with either HBV or HCV infection for survival analysis. High PPARγ expression was associated with poor survival in patients with HBV‐HCC, but the other lipogenesis genes were not correlated with OS (Figures 5A, 5C, 5E, and 5G). In contrast, high expression FAS and ACC significantly correlated with poor survival in HCV‐HCC patients (Figures 5B and 5D). PPARγ and SREBP1c also presented a trend of negative association with survival in HCV‐HCC patients, although a lack of statistical significance was observed (Figures 5F and 5H).

**FIGURE 6:**
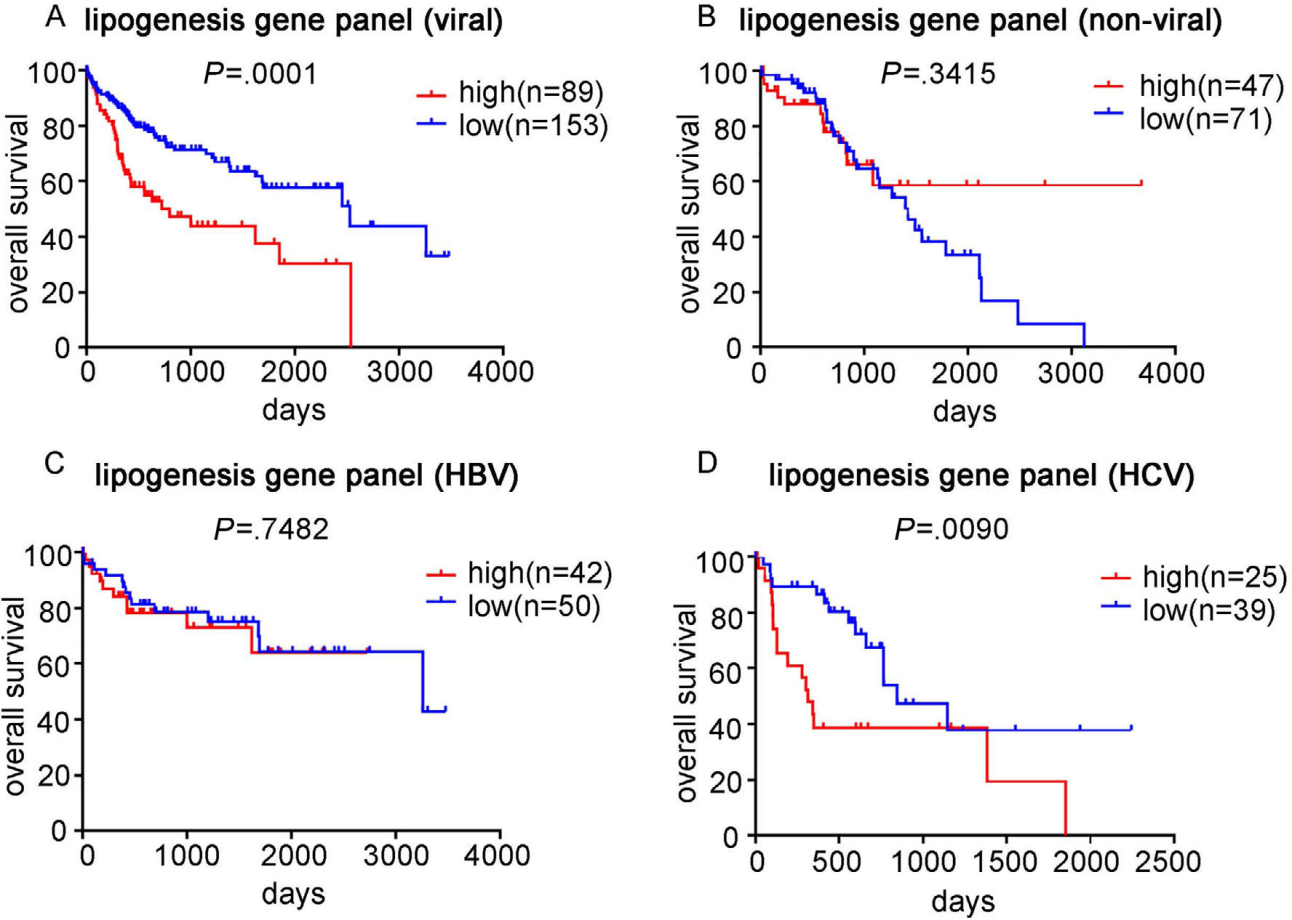
Analysis of the association between lipogenesis genes panel and survival outcome in HCC patients. The expression of FAS, ACC, PPARγ, and SREBP1c was considered as a lipogenesis genes panel. The Kaplan‐Meier statistical analysis was used to analyze the survival curves of HCC patients with different expression levels of the lipogenesis gene panel. Survival curve of the panel in viral HCC (A) and nonviral HCC (B) patients; survival curve of the panel in HBV‐HCC (C) and HCV‐HCC (D) patients

As expression of FAS, ACC, PPARγ, and SREBP1c was highly correlated (Table S5), we further analyzed their association with patient survival by considering them as a panel. Patients with three or more out of the four genes above the medians were considered lipogenesis high, otherwise, considered as lipogenesis low. Similar to the individual gene analysis, high lipogenesis significantly correlated with poor OS in viral HCC patients, but not in nonviral HCC patients (Figures 6A and 6B). Within the viral HCC group, the correlation between lipogenesis panel and survival only held in HCV‐HCC, but not in HBV‐HCC (Figures 6C and 6D).

## 4 DISCUSSION

Metabolic reprogramming is one of the hallmarks of cancer. In this study, we demonstrated that HCC elicited enhanced de novo lipogenesis, which is more pronounced in HCV‐HCC compared to HCC associated with other etiologies.

A complex combination of host, environmental, and viral factors may contribute to HCV‐related carcinogenesis. The induction of hepatocarcinogenesis is assumed to be mainly caused by the indirect effect of immune‐mediated chronic inflammation.^19^ However, HCV may also directly induce HCC by altering host regulatory pathways involved in proliferation, oxidative stress, and energy metabolism.^20^, ^21^ Dysregulated host lipid metabolism has been emerged as an additional factor that contributes to the pathogenesis of HCV‐induced HCC.^22^ Cumulating evidence has indicated that HCV infection can induce hepatic steatosis. It is believed that HCV induces hepatic de novo lipogenesis, which is evidenced by the upregulation of lipogenic genes, such as SREBP1c, FAS, ATP citrate lyase, and ACC, in HCV‐infected hepatic cell line,^22^ animal,^23^ and HCV transgenic mice.^24^ However, it is unclear whether the effect of HCV on lipid metabolism can also be seen in human HCV‐associated HCC. Here, we show that lipid accumulation is aberrantly high in human HCV‐HCC compared to control liver. Moreover, genes involved in de novo lipogenesis, such as AKT2, SREBP1c, PPARγ, ACC, and FAS, are dramatically upregulated in HCV‐HCC compared to control liver, suggesting robust activation of de novo lipogenesis. Another lipogenic transcription factor ChREBP also plays an important role in transcriptional activation of FAS and ACC genes.^25^ We found that ChREBP was significantly upregulated in human HCC compared to control liver, but there is no significant difference among HBV‐HCC, HCV‐HCC, and nonviral HCC. Activation of lipogenesis pathway was also seen in HBV‐HCC and nonviral HCC, but to a significantly less extent compared to that in HCV‐HCC. It suggests that the extent to which lipid metabolism is altered in HCC may be associated with etiologies. It is reported that HCV core proteins (including genotype 1b and 3) induce lipid accumulation in various HCC cell lines.^26^, ^27^ Moreover, core protein 1b can activate SREBP1 and PPARγ, which underlies the steatosis caused by HCV.^27^ Literatures documented that prevalence and mechanism of HCV‐induced steatosis appear to be genotype dependent. Steatosis occurs in 73% of patients infected with HCV genotype 3 (G3), and in 50% of patients infected with other genotypes.^28^ Lipid accumulation in G3 HCV‐infected patients appears to be a direct effect of the viral core proteins, but secondary to metabolic syndromes like insulin resistance and obesity in patients infected with other HCV genotypes.^29^ It is unclear whether the molecular mechanism of lipid accumulation is genotype dependent. A more accurate stratification of HCV‐HCC based on HCV genotype and cellular experiments with HCV proteins of different genotypes would be necessary to decipher the molecular mechanism of lipid accumulation in HCV‐HCC.

We also observed that high expression of lipogenic genes, regardless of whether considered individually or as a panel, was associated with poor survival only in viral HCC, but not in nonviral HCC, suggesting that aberrant activation of lipogenesis might be more important to cancer progression in the context of viral factors. Further stratifying the viral HCC group into HBV‐HCC and HCV‐HCC revealed that high expression of lipogenic genes still correlated with poor survival in HCC with only HCV background, suggesting activation of lipogenesis pathway to be a prognostic marker in HCV‐HCC. However, no significant correlation was seen between lipogenic genes expression and survival in HCC with only HBV background.

In conclusion, de novo lipogenesis is stimulated in human HCC. In particular, the level of de novo lipogenesis in HCV‐HCC was dramatically higher than that in HBV‐HCC and nonviral HCC. High expression of lipogenesis genes correlates with poor OS in HCV‐HCC patients. Given these findings, de novo lipogenesis pathway might be a potential target for anticancer drug and prognostic marker for HCC patients with HCV etiology.

## AUTHOR CONTRIBUTIONS

Geyang Xu, Genshu Wang, and Hui Chen designed the research. Shaojian Li, Ruonan Liu, Qinling Pan, Daorou Cheng, and Jie Yang performed the research. Shaojian Li, Ruonan Liu, Qinling Pan, Genshu Wang, and Daorou Cheng analyzed the data. Geyang Xu and Hui Chen wrote and revised the paper. Geyang Xu and Hui Chen are responsible for the integrity of the work as a whole.

## CONFLICT OF INTEREST

The authors declare no conflict of interest.

## Supporting information

Figure S1

Table S1

Table S2

Table S3

Table S4

Table S5
