# Supplementary material for: De novo lipogenesis is elicited dramatically in human hepatocellular carcinoma especially in hepatitis C virus‐induced hepatocellular carcinoma
Source: MedComm (2020). 2020 Jul 9;1(2):178–87. doi: 10.1002/mco2.15 (PMC8491216; doi:10.1002/mco2.15)
Supplement: Supplementary file 1 — Figure S1 [file MCO2-1-178-s005.docx]

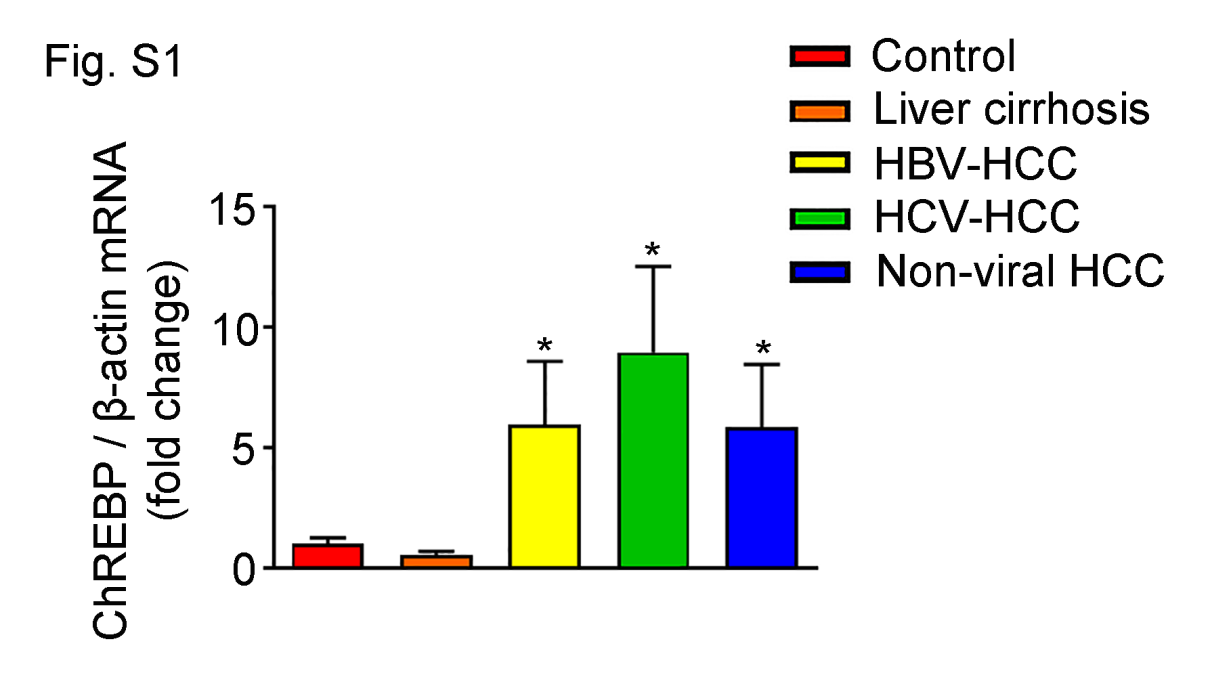


**Figure S1. Quantitative reverse transcription polymerase chain reaction analysis of ChREBP in human control livers, cirrhotic livers and HCC tissues.**

Results of quantitative PCR analysis of ChREBP mRNA in control human livers, cirrhotic livers, HBV-HCC, HCV-HCC and non-viral HCC tissues are expressed as fold change over control livers using β-actin as internal control. n=6, *P < 0.05 vs. control human livers.
