# Supplementary material for: De novo lipogenesis is elicited dramatically in human hepatocellular carcinoma especially in hepatitis C virus‐induced hepatocellular carcinoma
Source: MedComm (2020). 2020 Jul 9;1(2):178–87. doi: 10.1002/mco2.15 (PMC8491216; doi:10.1002/mco2.15)
Supplement: Supplementary file 2 — Table S1 [file MCO2-1-178-s004.docx]

| **Table S1. clinicopathological information of 18 HCC cases studied** | | |
| --- | --- | --- |
| **Classification** | **Subgroups** | **Numbers** |
| **Gender** | Male | 18 |
|  |  |  |
| **HBV or HCV Serology** | Positive | 12 |
|  | Negative | 6 |
|  |  |  |
| **Tumour size** | ≥50mm | 7 |
|  | <50mm | 11 |
|  |  |  |
| **Liver crirhosis** | Present | 12 |
|  | Absent | 6 |
|  |  |  |
| **T-stage** | T2 | 4 |
|  | T3 | 5 |
|  | T4 | 9 |
|  |  |  |
| **Multiple nodules** | ≥2 | 3 |
|  | 1 | 15 |
|  |  |  |
| **Macrovascular invasion** | Present | 11 |
|  | Absent | 7 |
|  |  |  |
