# Supplementary material for: De novo lipogenesis is elicited dramatically in human hepatocellular carcinoma especially in hepatitis C virus‐induced hepatocellular carcinoma
Source: MedComm (2020). 2020 Jul 9;1(2):178–87. doi: 10.1002/mco2.15 (PMC8491216; doi:10.1002/mco2.15)
Supplement: Supplementary file 3 — Table S2 [file MCO2-1-178-s006.docx]

| **Table S2. Clinical and biochemical characteristics of patients** | | | | | |
| --- | --- | --- | --- | --- | --- |
|  | **Control** | **Liver cirrhosis** | **HBV-HCC** | **HCV-HCC** | **Non-viral HCC** |
| **Age(year)** | 53.67 ± 4.16 | 49.60 ± 4.02 | 49.00 ± 11.02 | 59.00 ± 5.31 | 65.5 ± 4.29 |
| **Body weight (kg)** | 72.70 ± 7.28 | 63.00 ± 2.66 | 57.00 ± 1.53 | 65.50 ± 3.51 | 73.17 ± 2.90 |
| **Cholesterol (mmol/l)** | 6.33 ± 0.66 | 2.68 ± 0.39* | 4.04 ± 0.31* | 4.11 ± 0.31* | 4.10 ± 0.55* |
| **TG (mmol/l)** | 2.04 ± 0.40 | 0.73 ± 0.16* | 0.86 ± 0.23 | 1.28 ± 0.37 | 1.57 ± 0.42 |
| **LDL (mmol/l)** | 3.62 ± 0.58 | 1.21 ± 0.23* | 2.45 ± 0.40 | 2.64 ± 0.19 | 2.73 ± 0.39 |
| **HDL (mmol/l)** | 1.44 ± 0.18 | 0.23 ± 0.08* | 1.10 ± 0.18 | 0.91 ± 0.12* | 0.88 ± 0.05* |
| **Glucose (mmol/l)** | 5.48 ± 0.27 | 5.63 ± 1.39 | 5.27 ± 0.78 | 5.45 ± 0.26 | 7.69 ± 1.46 |
| **ALT(U/L)** | 22.83 ± 2.07 | 41.40 ± 3.88* | 43.00 ± 16.70 | 59.33 ± 11.42***** | 23.50 ± 4.72 |
| **AST(U/L)** | 21.00 ± 1.53 | 99.60 ± 14.33* | 42.67 ± 20.17 | 71.83 ± 9.94* | 40.67 ± 12.92 |

All patients undertook a 12-h fasting before blood collection for biochemical tests. Clinical biochemistry testing was conducted by Hitachi Automatic Biochemistry Analyzer (Hitachi High-technologies Corporation, Tokyo). ALT, alanine aminotransferase; AST, aspartate transaminase; HDL, high-density lipoprotein; LDL, low-density lipoprotein; TG, triglyceride. Data are mean ± SEM. Differences between groups were analyzed by Mann-Whitney U rank sum test. n = 6, *P < 0.05 vs. control.
