# Supplementary material for: De novo lipogenesis is elicited dramatically in human hepatocellular carcinoma especially in hepatitis C virus‐induced hepatocellular carcinoma
Source: MedComm (2020). 2020 Jul 9;1(2):178–87. doi: 10.1002/mco2.15 (PMC8491216; doi:10.1002/mco2.15)
Supplement: Supplementary file 4 — Table S3 [file MCO2-1-178-s003.doc]

| **Table S3. List and sequences of primers used in RT-PCR experiments.** | | | |
| --- | --- | --- | --- |
| **Gene name** | **Primer direction** | **Sequence** | **Accession number(s)** |
| **Human AKT2** | **sense** | GGTACTTCCTGCTGAAGAGC | NM_001330511.1 |
|  | **anti-sense** | ACTTCCATCTCCTCAGTCGT |  |
| **Human SREBP1c** | **sense** | CCATGGATTGCACTTTCGAA | NM_001321096.2 |
|  | **anti-sense** | GGCCAGGGAAGTCACTGTCTT |  |
| **Human PPARγ** | **sense** | CATGGCAATTGAATGTCGTGTC | NM_005037.6 |
|  | **anti-sense** | CCGGAAGAAACCCTTGCAT |  |
| **Human FAS** | **sense** | TATGCTTCTTCGTGCAGCAGTT | XM_011523538.2 |
|  | **anti-sense** | GCTGCCACACGCTCCTCTAG |  |
| **Human ACC** | **sense** | ATTGGGGCTTACCTTGTCCG | XM_005257267.5 |
|  | **anti-sense** | CGAGGACTTTGTTGAGGGCT |  |
| **Human ChREBP** | **sense**  **anti-sense** | CACACCAGCGTTTTGACCAG  AAGGACTCAAACAGAGGCCG | NM_032954.3 |
| **Human β-actin** | **sense** | TCATGAAGATCCTCACCGAG | NM_001101.4 |
|  | **anti-sense** | CATCTCTTGCTCGAAGTCCA |  |
|  |  |  |  |
