# Supplementary material for: De novo lipogenesis is elicited dramatically in human hepatocellular carcinoma especially in hepatitis C virus‐induced hepatocellular carcinoma
Source: MedComm (2020). 2020 Jul 9;1(2):178–87. doi: 10.1002/mco2.15 (PMC8491216; doi:10.1002/mco2.15)
Supplement: Supplementary file 5 — Table S4 [file MCO2-1-178-s002.pdf]

Table S4. HCC clinicopathological information from TCGA

1. HCC

barcode	gender	Age at diagnosis	histological type	FU OS status	FU days	pathologic stage	tnm.t	vascular tumor cell type	fibrosis ishak score	hist hepato carc fact	viral hepatitis serology	FAS	SREBF1	PPARG	ACACA
TCGA-ED-A7PZ	MALE	61	Hepatocellular Carcinoma	0	6	Stage II	T2	Micro	NotAvailable	No History of Primary Risk Factors	Hepatitis B Surface Antigen	46.22746544	36.98023366	1.355615273	4.014428525
TCGA-DD-AAVX	MALE	38	Hepatocellular Carcinoma	0	1570	Stage II	T2	Micro	6 - Established Cirrhosis	Hepatitis B	NotAvailable	22.35896454	8.210454049	4.283887727	2.327359447
TCGA-DD-A113	FEMALE	55	Hepatocellular Carcinoma	0	2425	Stage II	T2	Micro	0 - No Fibrosis	Other	Hepatitis B Surface Antigen	37.55177296	12.48839578	0.83243879	4.676007212
TCGA-CC-A5UC	MALE	63	Hepatocellular Carcinoma	1	347	Stage IIIA	T3	NotAvailable	NotAvailable	Alcohol consumption	Hepatitis C Antibody	352.3432693	58.6023195	10.3438332	12.03784196
TCGA-DD-AACX	MALE	66	Hepatocellular Carcinoma	0	170	Stage II	T2	None	3,4 - Fibrous Speta	Alcohol consumption	NotAvailable	110.3545815	40.22657352	0.828350478	6.308463591
TCGA-BC-A10U	MALE	69	Hepatocellular Carcinoma	1	837	NotAvailable	T2	NotAvailable	NotAvailable	Alcohol consumption	NotAvailable	68.25064326	44.57964375	3.248799811	4.507946886
TCGA-DD-AAE0	FEMALE	45	Hepatocellular Carcinoma	0	555	Stage IIIA	T3a	None	3,4 - Fibrous Speta	Hepatitis B	NotAvailable	27.44049215	12.43057362	0.861039863	4.428688781
TCGA-DD-A115	MALE	53	Hepatocellular Carcinoma	1	2542	Stage IIIA	T3	None	0 - No Fibrosis	Alcohol consumption	Hepatitis B Surface Antigen	97.60329393	17.36323724	2.243960685	3.83786457
TCGA-DD-AAE4	FEMALE	49	Hepatocellular Carcinoma	0	608	Stage I	T1	None	6 - Established Cirrhosis	Hepatitis B	NotAvailable	5.011447983	4.40811326	0.845980615	0.808653462
TCGA-DD-AAC8	MALE	72	Hepatocellular Carcinoma	1	16	Stage I	T1	Micro	1,2 - Portal Fibrosis	Hepatitis B	NotAvailable	45.58343487	6.222013285	7.219150775	3.044772198
TCGA-CC-A8HV	FEMALE	51	Hepatocellular Carcinoma	1	279	Stage II	T2	NotAvailable	NotAvailable	No History of Primary Risk Factors	Hepatitis C Antibody	155.878269	25.50374357	6.510655293	5.990620782
TCGA-DD-A73E	MALE	66	Hepatocellular Carcinoma	0	44	Stage I	T1	None	0 - No Fibrosis	Alcohol consumption	Hepatitis B Surface Antigen	94.98062956	8.186847636	7.503937599	5.741654166
TCGA-2Y-A9GU	FEMALE	55	Hepatocellular Carcinoma	0	1939	Stage I	T1	NotAvailable	NotAvailable	No History of Primary Risk Factors	Hepatitis C Antibody	123.2390458	41.29981819	2.842026877	2.910287395
TCGA-DD-A39W	FEMALE	29	Hepatocellular Carcinoma	1	827	Stage III	T3	None	NotAvailable	No History of Primary Risk Factors	NotAvailable	146.8902539	16.92549598	7.051987713	7.761207652
TCGA-EP-A26S	MALE	70	Hepatocellular Carcinoma	0	608	Stage I	T1	None	0 - No Fibrosis	Alcohol consumption	NotAvailable	95.07114356	41.68039782	3.200925301	5.208870163
TCGA-ED-A4XI	MALE	58	Hepatocellular Carcinoma	0	819	Stage II	T2	Micro	NotAvailable	No History of Primary Risk Factors	Hepatitis B Surface Antigen	45.64009449	38.3624941	3.235690722	2.354498647
TCGA-ZP-A9CZ	MALE	72	Hepatocellular Carcinoma	0	706	NotAvailable	T1	None	3,4 - Fibrous Speta	Hepatitis B	Hepatitis B Surface Antigen	111.4998549	14.23879312	1.721384415	6.456417677
TCGA-G3-A3CI	MALE	71	Hepatocellular Carcinoma	0	180	Stage I	T1	None	0 - No Fibrosis	Non-Alcoholic Fatty Liver Disease	NotAvailable	25.24240905	3.141053279	3.145160138	1.771717721
TCGA-ZP-A9D0	FEMALE	67	Hepatocellular Carcinoma	0	1091	NotAvailable	T1	None	0 - No Fibrosis	Non-Alcoholic Fatty Liver Disease	NotAvailable	14.12772427	6.776177729	2.522731601	0.976602205
TCGA-DD-AADU	MALE	60	Hepatocellular Carcinoma	0	554	Stage II	T2	Micro	3,4 - Fibrous Speta	Alcohol consumption	NotAvailable	18.74922347	7.354447331	4.061550401	1.138001701
TCGA-DD-A1EE	MALE	73	Hepatocellular Carcinoma	1	349	Stage IIIA	T3	None	6 - Established Cirrhosis	Hepatitis C	Hepatitis C Virus RNA	171.2477357	31.56232743	2.818505458	7.212838105
TCGA-GJ-A3OU	MALE	59	Hepatocellular Carcinoma	0	879	Stage I	T1	None	NotAvailable	Alcohol consumption	Hepatitis C Antibody	176.0277258	29.05646975	5.749406726	5.049172292
TCGA-CC-A7IJ	MALE	56	Hepatocellular Carcinoma	0	382	Stage II	T2	NotAvailable	NotAvailable	Alcohol consumption	Hepatitis C Antibody	29.40941146	53.18878536	9.95948377	3.85078456
TCGA-CC-A8HU	FEMALE	39	Hepatocellular Carcinoma	1	344	Stage IIIA	T3	NotAvailable	NotAvailable	No History of Primary Risk Factors	Hepatitis C Antibody	31.01665021	5.959179031	9.742199212	2.486943135
TCGA-G3-A5SK	MALE	58	Hepatocellular Carcinoma	0	744	Stage I	T1	NotAvailable	6 - Established Cirrhosis	Hepatitis C	NotAvailable	28.76042765	20.27476332	1.379266397	0.699957383
TCGA-DD-AAEA	MALE	65	Hepatocellular Carcinoma	0	575	Stage I	T1	None	1,2 - Portal Fibrosis	Alcohol consumption	NotAvailable	96.18540521	8.110674102	3.998302508	3.310961535
TCGA-XR-A8TG	MALE	58	Hepatocellular Carcinoma	0	898	Stage I	T1	None	6 - Established Cirrhosis	Hepatitis C	NotAvailable	29.71127635	18.91190129	5.289780207	3.04193043
TCGA-DD-A3A4	MALE	37	Hepatocellular Carcinoma	1	612	Stage IIIA	T3	None	0 - No Fibrosis	Other	NotAvailable	63.13735095	25.23407299	3.925411971	3.010452779
TCGA-DD-AAD8	FEMALE	73	Hepatocellular Carcinoma	0	1219	Stage I	T1	None	1,2 - Portal Fibrosis	Hepatitis B	NotAvailable	48.78757385	45.78684926	5.915565217	2.234575807
TCGA-BC-A10X	FEMALE	52	Hepatocellular Carcinoma	1	770	Stage IIIA	T3a	Micro	NotAvailable	No History of Primary Risk Factors	NotAvailable	42.2693865	24.72597022	2.330830076	1.669190239
TCGA-K7-A5RF	MALE	64	Hepatocellular Carcinoma	0	631	Stage I	T1	NotAvailable	NotAvailable	Hepatitis C	Hepatitis C Virus RNA	19.91464746	14.25842509	2.325291372	0.924920034
TCGA-ZS-A9CF	MALE	64	Hepatocellular Carcinoma	0	2412	Stage II	T2	Micro	0 - No Fibrosis	Hemochromatosis	Hepatitis C Antibody	48.54899912	31.76876975	1.28147202	3.710624129
TCGA-DD-AADS	MALE	63	Hepatocellular Carcinoma	0	474	Stage I	T1	None	NotAvailable	Alcohol consumption	NotAvailable	4.246434955	3.946627623	1.732744477	0.837478902
TCGA-2Y-A9GS	MALE	58	Hepatocellular Carcinoma	1	724	NotAvailable	T2	NotAvailable	NotAvailable	Alcohol consumption	HCV Genotype	98.8013014	21.3948724	5.409158543	4.759835724
TCGA-BC-A216	FEMALE	62	Hepatocellular Carcinoma	0	1351	Stage IIIA	T3	None	0 - No Fibrosis	No History of Primary Risk Factors	NotAvailable	74.63144503	88.87105276	4.719073507	4.193764586
TCGA-DD-A39X	FEMALE	78	Hepatocellular Carcinoma	1	1694	Stage I	T1	None	0 - No Fibrosis	No History of Primary Risk Factors	Hepatitis B Surface Antigen	42.31029664	14.22938512	2.459248121	2.455735285
TCGA-ZS-A9CG	MALE	55	Hepatocellular Carcinoma	0	341	Stage II	T2	Micro	0 - No Fibrosis	NotAvailable	Hepatitis C Antibody	36.07056351	4.940350321	2.65109278	1.94975614
TCGA-BC-A10W	MALE	50	Hepatocellular Carcinoma	1	91	NotAvailable	T4	NotAvailable	NotAvailable	Hepatitis B	Hepatitis B Surface Antigen	87.94499928	18.69450873	4.811624085	5.337103686
TCGA-DD-A118	FEMALE	77	Hepatocellular Carcinoma	0	3437	Stage II	T2	None	0 - No Fibrosis	NotAvailable	Hepatitis B Surface Antigen	37.11812007	18.34720355	3.820890589	2.754213445
TCGA-DD-AADW	MALE	48	Hepatocellular Carcinoma	0	587	Stage I	T1	None	6 - Established Cirrhosis	Hepatitis B	NotAvailable	262.0518876	21.64545907	3.160424607	5.889634834
TCGA-BC-A3KF	FEMALE	66	Hepatocellular Carcinoma	0	8	Stage I	T1	None	NotAvailable	No History of Primary Risk Factors	NotAvailable	81.78618545	23.24642317	4.344645087	3.871896326
TCGA-DD-A1EI	MALE	46	Hepatocellular Carcinoma	0	183	Stage I	T1	None	6 - Established Cirrhosis	Hepatitis B	Hepatitis B Surface Antigen	94.35856415	27.4027725	12.20716007	7.735490162
TCGA-DD-AAEE	MALE	55	Hepatocellular Carcinoma	0	810	Stage I	T1	None	6 - Established Cirrhosis	Hepatitis B	NotAvailable	43.45675107	14.04087585	4.773940965	5.659115523
TCGA-EP-A3JL	MALE	76	Hepatocellular Carcinoma	0	303	Stage I	T1	None	NotAvailable	Alcohol consumption	NotAvailable	40.91387732	15.25735805	3.093293347	1.91012291
TCGA-BD-A2L6	MALE	69	Hepatocellular Carcinoma	0	1363	NotAvailable	T2	Micro	3,4 - Fibrous Speta	Alcohol consumption	Hepatitis B Surface Antigen	50.44725633	18.1104137	4.263692782	1.939972906
TCGA-G3-A25Z	MALE	58	Hepatocellular Carcinoma	0	655	Stage I	T1	None	5 - Nodular Formation and Incomplete Cirrho	Hepatitis B	NotAvailable	155.4292121	6.432353775	4.536046234	4.065499824
TCGA-FV-A3R2	MALE	75	Hepatocellular Carcinoma	1	194	Stage I	T1	NotAvailable	NotAvailable	No History of Primary Risk Factors	Hepatitis C Antibody	60.63167256	13.09210556	7.827583761	6.430484664
TCGA-RC-A7SB	MALE	53	Hepatocellular Carcinoma	0	588	Stage II	T2	None	0 - No Fibrosis	Hepatitis B	NotAvailable	41.75390313	5.684825968	5.641269466	2.145437565
TCGA-5C-A9VH	MALE	70	Hepatocellular Carcinoma	0	322	Stage I	T1	None	NotAvailable	No History of Primary Risk Factors	NotAvailable	33.94873621	19.08315547	2.872009373	2.880274837
TCGA-5C-A9VG	MALE	58	Hepatocellular Carcinoma	0	328	Stage II	T2	Micro	NotAvailable	Other	NotAvailable	103.4613073	25.86997558	3.757217363	10.20932435
TCGA-CC-5261	MALE	44	Hepatocellular Carcinoma	1	97	Stage II	T2	None	NotAvailable	NotAvailable	Hepatitis C Antibody	61.75398231	14.65813544	2.322933777	4.668615557
TCGA-RC-A7SF	MALE	66	Hepatocellular Carcinoma	0	579	Stage I	T1	None	3,4 - Fibrous Speta	Hepatitis C	NotAvailable	287.3975975	70.03215383	1.871034719	3.449339248
TCGA-NI-A8LF	MALE	74	Hepatocellular Carcinoma	0	799	Stage I	T1	None	NotAvailable	Alcohol consumption	NotAvailable	150.9229965	6.634075063	7.67551391	3.906992301
TCGA-ED-A7PX	FEMALE	48	Hepatocellular Carcinoma	0	6	Stage II	T2	Micro	NotAvailable	No History of Primary Risk Factors	Hepatitis B Surface Antigen	77.28547677	23.27155821	1.170861519	13.37050353
TCGA-DD-A11D	FEMALE	57	Hepatocellular Carcinoma	1	1560	Stage I	T1	None	6 - Established Cirrhosis	Non-Alcoholic Fatty Liver Disease	NotAvailable	130.0341656	21.58690057	2.859404368	2.742900346
TCGA-DD-A1EB	FEMALE	72	Hepatocellular Carcinoma	0	2017	Stage I	T1	None	0 - No Fibrosis	Other	Hepatitis B Surface Antigen	386.3508109	12.19302708	2.515804759	6.410652568
TCGA-2Y-A9H7	FEMALE	81	Hepatocellular Carcinoma	0	1168	Stage I	T1	None	3,4 - Fibrous Speta	No History of Primary Risk Factors	Hepatitis C Antibody	74.78375467	41.39569846	7.671169932	5.583750946
TCGA-BC-A10Q	FEMALE	72	Hepatocellular Carcinoma	1	1135	NotAvailable	T2	None	NotAvailable	No History of Primary Risk Factors	NotAvailable	16.42605231	15.2541232	4.150982032	1.79817636
TCGA-G3-A25Y	FEMALE	52	Hepatocellular Carcinoma	1	452	Stage I	T1	None	3,4 - Fibrous Speta	Hepatitis B	NotAvailable	36.78101852	8.266749951	3.147699718	2.406178993
TCGA-DD-AAEH	MALE	73	Hepatocellular Carcinoma	0	784	Stage I	T1	Micro	1,2 - Portal Fibrosis	NotAvailable	NotAvailable	6.579694738	6.252246761	3.02425637	0.700111601
TCGA-4R-AA8I	MALE	66	Hepatocellular Carcinoma	1	262	Stage II	T2	Micro	6 - Established Cirrhosis	Non-Alcoholic Fatty Liver Disease	Hepatitis C Antibody	4.649227477	1.479036686	2.719484232	1.265169625
TCGA-DD-AAD2	MALE	66	Hepatocellular Carcinoma	0	658	Stage I	T1	Micro	1,2 - Portal Fibrosis	Hepatitis B	NotAvailable	48.88275503	35.62400519	3.899109004	3.563789701
TCGA-ZP-A9D1	FEMALE	56	Hepatocellular Carcinoma	0	21	NotAvailable	T1	None	5 - Nodular Formation and Incomplete Cirrho	Non-Alcoholic Fatty Liver Disease	NotAvailable	33.7998475	13.27867022	4.307229109	2.249402704
TCGA-DD-AACY	MALE	61	Hepatocellular Carcinoma	0	1450	Stage I	T1	None	3,4 - Fibrous Speta	Hepatitis B	NotAvailable	38.10785097	58.6561023	1.566370128	1.403780067
TCGA-OA-A7B7	MALE	48	Hepatocellular Carcinoma	0	94	Stage II	T2	Micro	0 - No Fibrosis	Hepatitis B	NotAvailable	558.5990297	9.341860448	0.735383118	7.307906855
TCGA-KR-A7K7	FEMALE	61	Hepatocellular Carcinoma	0	951	Stage II	T2	Micro	NotAvailable	No History of Primary Risk Factors	NotAvailable	22.55157517	5.1310501	5.20713003	2.407748896
TCGA-DD-AAVO	MALE	38	Hepatocellular Carcinoma	0	2728	Stage I	T1	None	6 - Established Cirrhosis	Hepatitis B	NotAvailable	67.83390497	18.06255302	3.459593768	2.928457054
TCGA-DD-A11A	MALE	67	Hepatocellular Carcinoma	0	79	Stage I	T1	None	0 - No Fibrosis	Other	Hepatitis B Surface Antigen	202.7973703	22.08159038	11.45240665	9.099840515
TCGA-DD-AADG	MALE	70	Hepatocellular Carcinoma	0	1145	Stage IIIA	T3a	None	3,4 - Fibrous Speta	Alcohol consumption	NotAvailable	320.9120891	16.91143286	1.5886101	8.517032559
TCGA-G3-A7M7	MALE	65	Hepatocellular Carcinoma	0	361	Stage I	T1	None	3,4 - Fibrous Speta	Alcohol consumption	Hepatitis C Antibody	23.62952323	19.86512303	2.349289987	1.983621931
TCGA-BC-A5W4	MALE	69	Hepatocellular Carcinoma	1	547	Stage IIIA	T3a	Micro	NotAvailable	Alcohol consumption	NotAvailable	24.46506493	7.392713554	4.002043076	2.183088487
TCGA-DD-AAW2	MALE	69	Hepatocellular Carcinoma	0	1855	Stage I	T1	None	6 - Established Cirrhosis	Non-Alcoholic Fatty Liver Disease	NotAvailable	42.32141451	47.15192199	3.38816584	2.684991279
TCGA-DD-AADI	FEMALE	43	Hepatocellular Carcinoma	0	1085	Stage I	T1	None	NotAvailable	Hepatitis B	NotAvailable	13.16556356	11.66794234	2.302309092	1.581880434
TCGA-DD-A3A7	MALE	67	Hepatocellular Carcinoma	1	419	Stage IIIB	T3b	Micro	0 - No Fibrosis	Alcohol consumption	Hepatitis B Surface Antigen	75.99395026	81.91079604	1.5924461	4.251626928
TCGA-CC-A9FW	MALE	68	Hepatocellular Carcinoma	0	248	Stage IIIA	T3	NotAvailable	NotAvailable	Alcohol consumption	Hepatitis C Antibody	172.8680083	33.56407948	13.13794349	9.910166657
TCGA-RC-A7S9	FEMALE	47	Hepatocellular Carcinoma	0	640	Stage I	T1	None	6 - Established Cirrhosis	Hepatitis B	NotAvailable	28.17905612	52.23758414	1.667180482	2.776343015
TCGA-3K-AAZ8	MALE	65	Hepatocellular Carcinoma	0	396	Stage IIIB	T3b	NotAvailable	5 - Nodular Formation and Incomplete Cirrho	Hepatitis C	NotAvailable	42.17715464	9.989275835	4.237541694	2.892015052
TCGA-MI-A75I	MALE	61	Hepatocellular Carcinoma	0	630	NotAvailable	T2	NotAvailable	NotAvailable	Hepatitis C	NotAvailable	155.1220033	14.44159457	4.951347417	4.785145094
TCGA-5R-AA1D	FEMALE	17	Hepatocellular Carcinoma	0	449	Stage IIIA	T3a	Micro	0 - No Fibrosis	No History of Primary Risk Factors	Hepatitis C Antibody	9.008337356	6.456495899	3.619471077	1.423569143
TCGA-DD-A116	MALE	68	Hepatocellular Carcinoma	1	1622	Stage IIIA	T3	NotAvailable	3,4 - Fibrous Speta	Hepatitis B	Hepatitis B Surface Antigen	82.14236987	33.28550893	4.604862931	4.63919003
TCGA-BD-A3EP	FEMALE	75	Hepatocellular Carcinoma	0	409	Stage I	T1	None	1,2 - Portal Fibrosis	No History of Primary Risk Factors	NotAvailable	126.5758919	13.19447854	7.077346344	2.779479635
TCGA-CC-A123	FEMALE	24	Hepatocellular Carcinoma	0	219	Stage IIIA	T3	None	NotAvailable	NotAvailable	Hepatitis C Antibody	41.57970014	8.059349312	11.49412111	4.017051535
TCGA-DD-AAVW	MALE	35	Hepatocellular Carcinoma	0	2317	Stage I	T1	None	6 - Established Cirrhosis	Hepatitis B	NotAvailable	41.9977929	12.34449204	4.710399125	2.932025793
TCGA-DD-A4NR	FEMALE	85	Hepatocellular Carcinoma	1	9	Stage I	T1	None	6 - Established Cirrhosis	Other	Hepatitis B Surface Antigen	149.5191275	42.15121392	5.575209299	5.778894284
TCGA-DD-AAEG	FEMALE	59	Hepatocellular Carcinoma	0	719	Stage I	T1	None	6 - Established Cirrhosis	No History of Primary Risk Factors	NotAvailable	52.80917949	10.5030274	1.882628961	1.909960486
TCGA-DD-A1EA	MALE	68	Hepatocellular Carcinoma	0	2415	Stage II	T2	Micro	1,2 - Portal Fibrosis	Hepatitis B	Hepatitis B Surface Antigen	96.00189831	7.629237594	2.630774095	5.450202707
TCGA-BC-A110	FEMALE	51	Hepatocellular Carcinoma	1	2116	NotAvailable	T1	None	NotAvailable	Alcohol consumption	NotAvailable	14.2600088	7.724585331	3.035238171	0.728939998
TCGA-BW-A5NP	FEMALE	26	Hepatocellular Carcinoma	0	0	Stage IV	T2	Micro	3,4 - Fibrous Speta	Hepatitis B	NotAvailable	65.60107808	35.17188812	12.33693715	4.092725003
TCGA-DD-A73B	FEMALE	72	Hepatocellular Carcinoma	1	283	Stage I	T1	None	6 - Established Cirrhosis	Alpha-1 Antitrypsin Deficiency	Hepatitis B Surface Antigen	61.21888336	9.746508163	1.678044937	4.628285428
TCGA-ED-A66Y	FEMALE	51	Hepatocellular Carcinoma	1	296	Stage IIIA	T3a	Micro	NotAvailable	No History of Primary Risk Factors	Hepatitis B Surface Antigen	44.96633728	61.6022001	7.372068179	4.023831366
TCGA-CC-A7II	MALE	54	Hepatocellular Carcinoma	0	399	Stage IIIA	T3	NotAvailable	NotAvailable	Alcohol consumption	Hepatitis C Antibody	32.56525405	6.066664527	5.636599642	6.598449614
TCGA-DD-AACT	FEMALE	69	Hepatocellular Carcinoma	0	1562	Stage I	T1	None	NotAvailable	Hepatitis B	NotAvailable	37.80510244	8.062792877	2.241004666	1.343692835
TCGA-CC-5263	MALE	35	Hepatocellular Carcinoma	1	129	Stage IIIA	T3	None	NotAvailable	No History of Primary Risk Factors	Hepatitis C Antibody	78.25763913	20.37243958	4.156819839	4.864728773
TCGA-DD-AADV	MALE	50	Hepatocellular Carcinoma	0	574	Stage I	T1	None	6 - Established Cirrhosis	Alcohol consumption	NotAvailable	32.85571063	9.676485955	6.602604716	1.315358049
TCGA-EP-A2KB	FEMALE	46	Hepatocellular Carcinoma	1	596	Stage I	T1	None	NotAvailable	Hepatitis C	NotAvailable	27.27083342	10.5315698	15.35883156	3.258766832
TCGA-DD-AACA	MALE	65	Hepatocellular Carcinoma	0	2301	Stage I	T1	None	6 - Established Cirrhosis	Hepatitis B	NotAvailable	147.2871873	21.81553581	8.340089259	9.258887475
TCGA-ZS-A9CD	MALE	73	Hepatocellular Carcinoma	1	1386	Stage II	T2	None	5 - Nodular Formation and Incomplete Cirrho	NotAvailable	Hepatitis C Antibody	57.68075079	20.24077171	2.620381115	2.046700612
TCGA-K7-A5RG	MALE	66	Hepatocellular Carcinoma	0	519	Stage I	T1	None	NotAvailable	Alcohol consumption	HCV Genotype	27.90201077	12.61613667	8.879424931	2.455176467
TCGA-WX-AA46	MALE	61	Hepatocellular Carcinoma	0	756	Stage II	T2	Macro	0 - No Fibrosis	No History of Primary Risk Factors	Hepatitis C Antibody	44.56836968	8.026018694	2.317561233	1.531956756
TCGA-EP-A2KA	FEMALE	52	Hepatocellular Carcinoma	1	627	Stage IIIA	T3a	NotAvailable	1,2 - Portal Fibrosis	No History of Primary Risk Factors	NotAvailable	30.09406873	57.00053874	7.458825337	3.081580811
TCGA-5R-AA1C	MALE	57	Hepatocellular Carcinoma	0	520	Stage II	T2	Micro	1,2 - Portal Fibrosis	No History of Primary Risk Factors	Hepatitis C Antibody	13.86386392	4.106873862	5.85559917	2.737206407
TCGA-ED-A82E	FEMALE	60	Hepatocellular Carcinoma	0	408	Stage IIIA	T3a	Micro	NotAvailable	No History of Primary Risk Factors	Hepatitis C Antibody	11.07098885	4.943530191	0.579661454	1.481110803
TCGA-DD-AACG	MALE	52	Hepatocellular Carcinoma	1	469	Stage II	T2	Micro	6 - Established Cirrhosis	Hepatitis B	NotAvailable	28.00462612	13.82738582	11.6920013	4.848896807
TCGA-UB-A7MC	MALE	59	Hepatocellular Carcinoma	0	500	Stage IIIA	T3a	Micro	1,2 - Portal Fibrosis	Alcohol consumption	Hepatitis C Antibody	35.2313773	3.809392027	3.178604588	4.675241482
TCGA-DD-A119	MALE	40	Hepatocellular Carcinoma	1	223	Stage IV	T3a	Micro	0 - No Fibrosis	Hepatitis B	Hepatitis B Surface Antigen	15.26215757	7.181914193	1.658046016	1.648328917
TCGA-G3-AAV2	MALE	50	Hepatocellular Carcinoma	0	372	Stage I	T1	None	6 - Established Cirrhosis	Alcohol consumption	Hepatitis C Antibody	60.06901043	16.64311692	3.161845879	2.397366969
TCGA-CC-A3MC	MALE	54	Hepatocellular Carcinoma	0	363	Stage IIIA	T3	NotAvailable	NotAvailable	Alcohol consumption	Hepatitis C Antibody	88.0342438	8.529882528	2.413007846	3.33901839
TCGA-DD-AACH	MALE	69	Hepatocellular Carcinoma	1	195	Stage II	T2	Micro	6 - Established Cirrhosis	Hepatitis B	NotAvailable	79.05129227	15.20669634	8.87366793	4.840465581
TCGA-G3-A3CG	MALE	80	Hepatocellular Carcinoma	0	673	Stage I	T1	Micro	6 - Established Cirrhosis	Hepatitis C	NotAvailable	261.6235756	102.8241115	2.40491564	5.145438345
TCGA-CC-A3MB	MALE	36	Hepatocellular Carcinoma	1	315	Stage IIIA	T3	NotAvailable	NotAvailable	No History of Primary Risk Factors	Hepatitis C Antibody	103.513112	65.70120654	6.308582346	7.871580521
TCGA-DD-AAD5	MALE	54	Hepatocellular Carcinoma	0	1345	Stage I	T1	None	3,4 - Fibrous Speta	Alcohol consumption	NotAvailable	65.68215061	56.9462891	8.188373036	7.886950092
TCGA-ED-A627	MALE	74	Hepatocellular Carcinoma	0	423	Stage I	T1	NotAvailable	NotAvailable	Alcohol consumption	Hepatitis C Antibody	27.78633922	16.46697227	2.436506499	1.543201446
TCGA-ZP-A9CV	MALE	59	Hepatocellular Carcinoma	1	1088	NotAvailable	T1	None	NotAvailable	Alcohol consumption	NotAvailable	96.25878612	60.25695281	2.349004373	3.45344669
TCGA-G3-A25T	FEMALE	45	Hepatocellular Carcinoma	0	1553	Stage IIIA	T3	None	0 - No Fibrosis	Hepatitis C	NotAvailable	9.840705427	15.43974363	2.430765624	1.531885334
TCGA-DD-A1EK	FEMALE	64	Hepatocellular Carcinoma	1	558	Stage IVB	T4	None	0 - No Fibrosis	Other	Hepatitis B Surface Antigen	101.3639657	19.56154136	1.822307465	4.55990854
TCGA-KR-A7K8	MALE	57	Hepatocellular Carcinoma	0	906	Stage I	T1	None	NotAvailable	No History of Primary Risk Factors	NotAvailable	90.26559562	22.11520485	7.671383204	1.107703422
TCGA-CC-A9FU	FEMALE	52	Hepatocellular Carcinoma	0	0	Stage IIIA	T3a	NotAvailable	NotAvailable	Hepatitis B	Hepatitis C Antibody	128.227866	30.52904403	21.00258977	6.118658936
TCGA-HP-A5N0	FEMALE	90	Hepatocellular Carcinoma	1	1147	NotAvailable	TX	NotAvailable	0 - No Fibrosis	No History of Primary Risk Factors	NotAvailable	39.84123449	7.915800113	2.759848932	1.640960717
TCGA-DD-AACN	MALE	32	Hepatocellular Carcinoma	0	1302	Stage I	T1	None	NotAvailable	Hepatitis B	NotAvailable	28.06110146	10.23873081	2.873206573	1.983358972
TCGA-2Y-A9GZ	FEMALE	82	Hepatocellular Carcinoma	1	848	Stage II	T2	NotAvailable	NotAvailable	No History of Primary Risk Factors	Hepatitis C Antibody	153.7925495	2.586303624	5.29106502	1.653412624
TCGA-DD-AADP	MALE	45	Hepatocellular Carcinoma	0	458	Stage I	T1	Micro	NotAvailable	Hepatitis B	NotAvailable	171.6408011	63.52753909	3.610401721	6.288990917
TCGA-2Y-A9HA	MALE	70	Hepatocellular Carcinoma	1	36	Stage II	T2	NotAvailable	3,4 - Fibrous Speta	Alcohol consumption	Hepatitis C Antibody	85.42310782	11.87389198	9.333454665	4.979738932
TCGA-NI-A4U2	MALE	71	Hepatocellular Carcinoma	1	1791	Stage IIIA	T3	None	1,2 - Portal Fibrosis	Alcohol consumption	NotAvailable	55.27210876	6.184697907	4.59662587	1.643960598
TCGA-DD-A39Y	MALE	67	Hepatocellular Carcinoma	1	171	Stage I	T1	None	0 - No Fibrosis	No History of Primary Risk Factors	Hepatitis B Surface Antigen	166.4086798	33.68100221	13.24174901	6.59828502
TCGA-G3-A5SL	MALE	70	Hepatocellular Carcinoma	0	621	Stage II	T2	Micro	0 - No Fibrosis	Alcohol consumption	NotAvailable	159.8903676	6.353057295	3.200623583	10.28536525
TCGA-DD-A73D	FEMALE	68	Hepatocellular Carcinoma	0	693	Stage II	T2	Micro	6 - Established Cirrhosis	Hepatitis C	Hepatitis C Virus RNA	36.62111796	3.321474261	4.871095444	3.766990665
TCGA-UB-AA0U	MALE	60	Hepatocellular Carcinoma	0	327	Stage II	T2	None	0 - No Fibrosis	Non-Alcoholic Fatty Liver Disease	Hepatitis C Antibody	110.6991527	19.68872744	3.228452273	5.064336023
TCGA-BD-A3ER	MALE	62	Hepatocellular Carcinoma	0	1115	Stage II	T2	None	6 - Established Cirrhosis	Hemochromatosis	Hepatitis B Surface Antigen	70.31048954	30.08199124	1.68426832	3.468893691
TCGA-FV-A495	FEMALE	51	Hepatocellular Carcinoma	0	1	Stage II	T2	None	3,4 - Fibrous Speta	Hepatitis C	NotAvailable	69.45027742	23.13687422	2.920166597	1.963071364
TCGA-DD-A3A3	MALE	45	Hepatocellular Carcinoma	1	535	Stage I	T1	Macro	0 - No Fibrosis	No History of Primary Risk Factors	NotAvailable	67.61481263	10.99815801	2.574215355	3.532728402
TCGA-DD-AAVY	MALE	56	Hepatocellular Carcinoma	0	1970	Stage IIIA	T3	Micro	0 - No Fibrosis	No History of Primary Risk Factors	NotAvailable	50.43033876	13.73000775	2.67330824	3.929209134
TCGA-2Y-A9HB	MALE	66	Hepatocellular Carcinoma	0	260	Stage I	T1	None	NotAvailable	Alcohol consumption	Hepatitis C Virus RNA	113.4226544	73.71895475	1.609301666	5.745996483
TCGA-KR-A7K0	MALE	65	Hepatocellular Carcinoma	1	65	Stage I	T1	None	NotAvailable	Alcohol consumption	Hepatitis C Antibody	45.18377581	9.881454938	3.782142061	2.524280926
TCGA-DD-A4NF	MALE	72	Hepatocellular Carcinoma	0	942	Stage I	T1	None	6 - Established Cirrhosis	Hepatitis C	Hepatitis C Virus RNA	38.53690401	11.80172355	4.843333056	5.053814754
TCGA-XR-A8TF	MALE	74	Hepatocellular Carcinoma	1	693	Stage I	T1	None	3,4 - Fibrous Speta	Hepatitis B	NotAvailable	30.52110704	8.266115558	4.50976608	2.120107984
TCGA-WX-AA47	FEMALE	33	Hepatocellular Carcinoma	1	556	Stage IIIA	T3a	Macro	0 - No Fibrosis	No History of Primary Risk Factors	Hepatitis C Antibody	77.82479241	8.521244602	1.749905076	1.623915939
TCGA-DD-A4NI	MALE	67	Hepatocellular Carcinoma	0	816	Stage II	T2	None	0 - No Fibrosis	Alcohol consumption	Hepatitis B Surface Antigen	54.3276073	10.53683623	1.71136376	1.460707925
TCGA-BW-A5NQ	MALE	63	Hepatocellular Carcinoma	0	0	Stage I	T1	None	6 - Established Cirrhosis	Alcohol consumption	NotAvailable	71.40372799	7.949008686	11.77911903	7.078588924
TCGA-CC-5260	FEMALE	61	Hepatocellular Carcinoma	1	87	Stage IIIC	T4	None	NotAvailable	NotAvailable	Hepatitis C Antibody	31.59466464	22.69848223	4.545498608	5.288087613
TCGA-FV-A3I1	FEMALE	81	Hepatocellular Carcinoma	1	247	Stage II	T2	Micro	0 - No Fibrosis	Alcohol consumption	Hepatitis C Antibody	239.4858935	36.37915381	6.825140981	5.583106964
TCGA-DD-A1EC	FEMALE	20	Hepatocellular Carcinoma	0	602	Stage I	T1	None	0 - No Fibrosis	NotAvailable	NotAvailable	28.54724958	31.79494068	2.446980626	6.124338501
TCGA-FV-A3R3	FEMALE	38	Hepatocellular Carcinoma	1	366	Stage I	T1	None	NotAvailable	No History of Primary Risk Factors	NotAvailable	12.41658589	24.99075418	4.492028329	2.553023486
TCGA-DD-AAVS	MALE	56	Hepatocellular Carcinoma	0	1823	Stage I	T1	None	3,4 - Fibrous Speta	Hepatitis B	NotAvailable	33.12415257	21.21615866	4.936105949	2.527253166
TCGA-YA-A8S7	MALE	68	Hepatocellular Carcinoma	1	412	Stage IIIA	T3a	None	NotAvailable	Hepatitis C	NotAvailable	22.81635384	13.87341997	7.75784656	2.827828214
TCGA-O8-A75V	MALE	54	Hepatocellular Carcinoma	0	538	Stage I	T1	None	6 - Established Cirrhosis	Alcohol consumption	NotAvailable	55.54877714	22.99367399	3.913312835	3.430389811
TCGA-BC-A69H	MALE	64	Hepatocellular Carcinoma	0	444	Stage II	T2	Micro	NotAvailable	Alcohol consumption	NotAvailable	197.5220198	32.85330165	18.4217719	8.272651453
TCGA-DD-AAED	MALE	51	Hepatocellular Carcinoma	0	763	Stage I	T1	None	1,2 - Portal Fibrosis	Alcohol consumption	NotAvailable	21.12528064	33.71987663	7.589523711	4.099811253
TCGA-RC-A6M4	FEMALE	74	Hepatocellular Carcinoma	0	22	Stage IIIA	T3	None	NotAvailable	No History of Primary Risk Factors	NotAvailable	131.530462	30.06255001	2.732691975	7.381488159
TCGA-DD-AACP	MALE	64	Hepatocellular Carcinoma	0	415	Stage I	T1	None	NotAvailable	Alcohol consumption	NotAvailable	117.2442141	19.75110673	12.48199463	6.509160985
TCGA-DD-A73G	FEMALE	73	Hepatocellular Carcinoma	0	3478	Stage I	T1	None	0 - No Fibrosis	No History of Primary Risk Factors	Hepatitis B Surface Antigen	39.65254989	41.22614334	3.323730246	2.370609157
TCGA-FV-A4ZP	MALE	78	Hepatocellular Carcinoma	1	2486	Stage IIIA	T3	Micro	NotAvailable	Alcohol consumption	NotAvailable	57.97509595	26.25301525	2.279576204	2.260646115
TCGA-UB-AA0V	FEMALE	69	Hepatocellular Carcinoma	0	314	Stage I	NotAvailable	None	0 - No Fibrosis	Hemochromatosis	Hepatitis C Antibody	67.43549711	17.50065179	1.74723534	1.78959338
TCGA-2Y-A9GT	MALE	51	Hepatocellular Carcinoma	1	1624	Stage I	T1	None	NotAvailable	Alcohol consumption	Hepatitis C Virus RNA	67.41940699	32.84095054	3.369772649	3.403193796
TCGA-WJ-A86L	FEMALE	68	Hepatocellular Carcinoma	0	345	Stage I	T1	None	0 - No Fibrosis	No History of Primary Risk Factors	Hepatitis C Antibody	100.0201622	26.37742503	0.303370104	7.636300942
TCGA-CC-A7IE	MALE	57	Hepatocellular Carcinoma	1	217	Stage IIIA	T3	NotAvailable	NotAvailable	Alcohol consumption	Hepatitis C Antibody	129.8837692	80.901433	10.61883058	5.505385886
TCGA-G3-A3CJ	MALE	52	Hepatocellular Carcinoma	0	594	Stage II	T2	Micro	5 - Nodular Formation and Incomplete Cirrho	Hepatitis C	NotAvailable	54.49914199	12.26989785	2.895612104	2.727803972
TCGA-MR-A520	MALE	58	Hepatocellular Carcinoma	0	229	Stage I	T1	NotAvailable	NotAvailable	Other	Hepatitis C Antibody	71.66417691	4.372529035	1.891067018	2.946878084
TCGA-DD-AAE1	MALE	52	Hepatocellular Carcinoma	0	552	Stage I	T1	None	6 - Established Cirrhosis	Hepatitis B	NotAvailable	48.14240483	43.1189918	10.84294068	4.034345989
TCGA-LG-A9OC	MALE	48	Hepatocellular Carcinoma	0	425	Stage I	T1	Micro	NotAvailable	No History of Primary Risk Factors	NotAvailable	324.1306685	43.01674817	2.834358622	4.627048985
TCGA-BC-A217	FEMALE	75	Hepatocellular Carcinoma	1	1397	Stage II	T2	Micro	0 - No Fibrosis	Alcohol consumption	NotAvailable	26.68174018	14.95798181	5.264557559	1.920575177
TCGA-ED-A8O5	FEMALE	59	Hepatocellular Carcinoma	0	406	Stage IIIA	T3a	Micro	NotAvailable	No History of Primary Risk Factors	Hepatitis C Antibody	49.29493513	24.37789971	9.861459031	1.685658837
TCGA-DD-AADL	MALE	58	Hepatocellular Carcinoma	0	636	Stage I	T1	Micro	NotAvailable	Alcohol consumption	NotAvailable	65.1943466	15.25482267	4.070707072	5.101438915
TCGA-2Y-A9H9	MALE	70	Hepatocellular Carcinoma	0	697	Stage I	T1	NotAvailable	NotAvailable	Alcohol consumption	NotAvailable	74.71218183	5.73465591	2.113919225	2.595891863
TCGA-G3-A5SJ	MALE	59	Hepatocellular Carcinoma	0	698	Stage I	T1	None	6 - Established Cirrhosis	Alcohol consumption	NotAvailable	13.38628109	41.74749306	3.420392505	2.514032376
TCGA-DD-A4NA	FEMALE	67	Hepatocellular Carcinoma	0	1008	Stage IIIC	T2	NotAvailable	5 - Nodular Formation and Incomplete Cirrho	No History of Primary Risk Factors	Hepatitis B Surface Antigen	8.255566614	8.931317612	0.559144223	3.478317229
TCGA-G3-AAV3	FEMALE	58	Hepatocellular Carcinoma	0	412	Stage II	T2	None	6 - Established Cirrhosis	Alcohol consumption	NotAvailable	39.76988148	8.760149078	3.81974026	2.376179135
TCGA-DD-A1ED	MALE	68	Hepatocellular Carcinoma	0	2301	Stage I	T1	None	0 - No Fibrosis	Alcohol consumption	Hepatitis B Surface Antigen	10.5533033	11.09449834	3.353769163	1.282369685
TCGA-CC-A9FS	MALE	55	Hepatocellular Carcinoma	0	211	Stage II	T2	NotAvailable	NotAvailable	Alcohol consumption	Hepatitis C Antibody	35.60560074	6.113524488	3.1568057	2.03757406
TCGA-UB-A7MB	MALE	24	Hepatocellular Carcinoma	0	601	Stage II	T2	Micro	0 - No Fibrosis	No History of Primary Risk Factors	Hepatitis C Antibody	333.3578943	18.95175337	4.751915422	11.31364229
TCGA-G3-AAV0	MALE	58	Hepatocellular Carcinoma	0	476	Stage I	T1	None	0 - No Fibrosis	Hepatitis B	Hepatitis C Antibody	68.17217375	40.95374397	2.125075221	1.25569147
TCGA-MI-A75E	MALE	61	Hepatocellular Carcinoma	0	507	Stage IIIC	T4	Micro	1,2 - Portal Fibrosis	Alcohol consumption	HCV Genotype	79.82198185	19.55364563	2.217183565	4.129149759
TCGA-G3-AAV5	MALE	67	Hepatocellular Carcinoma	0	354	Stage II	T2	Macro	6 - Established Cirrhosis	Alcohol consumption	Hepatitis C Antibody	69.65336911	11.20071485	2.010958654	7.471867326
TCGA-2Y-A9H2	FEMALE	64	Hepatocellular Carcinoma	0	1731	Stage I	T1	None	NotAvailable	Alcohol consumption	Hepatitis C Antibody	29.88083286	15.7629821	1.033468664	3.645373348
TCGA-G3-A6UC	MALE	65	Hepatocellular Carcinoma	0	671	Stage IIIB	T3b	Micro	6 - Established Cirrhosis	Alcohol consumption	Hepatitis C Antibody	80.27290176	5.632139759	2.687058356	2.241479214
TCGA-BC-A10Z	FEMALE	62	Hepatocellular Carcinoma	1	34	Stage I	T1	None	NotAvailable	No History of Primary Risk Factors	NotAvailable	534.7769863	31.48728426	10.59181365	18.56039969
TCGA-BC-A10T	MALE	76	Hepatocellular Carcinoma	1	837	NotAvailable	T4	None	1,2 - Portal Fibrosis	No History of Primary Risk Factors	NotAvailable	94.96374176	11.75179443	1.073698718	4.769200853
TCGA-G3-A25V	MALE	68	Hepatocellular Carcinoma	0	860	Stage I	T1	None	6 - Established Cirrhosis	Alcohol consumption	NotAvailable	41.26864913	23.72280415	2.985473228	2.132132203
TCGA-ZP-A9D2	MALE	51	Hepatocellular Carcinoma	1	765	NotAvailable	T2	Micro	1,2 - Portal Fibrosis	Hepatitis C	Hepatitis C Virus RNA	23.37276315	4.292433642	1.029497401	6.084070048
TCGA-CC-A3MA	MALE	61	Hepatocellular Carcinoma	1	303	Stage IIIA	T3	NotAvailable	NotAvailable	Alcohol consumption	Hepatitis C Antibody	13.65698766	10.67180836	0.682023006	4.805025691
TCGA-2Y-A9H4	MALE	68	Hepatocellular Carcinoma	0	1452	Stage I	T1	None	NotAvailable	Alcohol consumption	Hepatitis C Antibody	8.302740828	2.114172259	1.368243722	1.593188134
TCGA-DD-AACW	MALE	43	Hepatocellular Carcinoma	0	1424	Stage I	T1	None	NotAvailable	Alcohol consumption	NotAvailable	43.43051679	44.27545585	12.50710244	4.596389763
TCGA-T1-A6J8	MALE	68	Hepatocellular Carcinoma	0	23	NotAvailable	T1	None	NotAvailable	Alcohol consumption	NotAvailable	31.9874301	9.297626914	6.837452883	2.410825597
TCGA-DD-A3A5	FEMALE	66	Hepatocellular Carcinoma	1	3125	Stage III	T3	None	0 - No Fibrosis	No History of Primary Risk Factors	NotAvailable	39.6665923	14.1544423	0.703398688	0.671737332
TCGA-DD-A4NS	FEMALE	61	Hepatocellular Carcinoma	1	2456	Stage I	T1	None	0 - No Fibrosis	Alcohol consumption	Hepatitis B Surface Antigen	77.08213492	31.99487944	2.832926042	2.500892995
TCGA-DD-AACV	MALE	53	Hepatocellular Carcinoma	0	1531	Stage I	T1	Micro	NotAvailable	Alcohol consumption	NotAvailable	84.27829016	19.31048376	1.102194105	2.486544788
TCGA-CC-A8HS	MALE	18	Hepatocellular Carcinoma	1	300	Stage IIIC	T3	NotAvailable	NotAvailable	No History of Primary Risk Factors	Hepatitis C Antibody	63.29733083	44.74855523	5.26277986	5.812341338
TCGA-2Y-A9GW	MALE	64	Hepatocellular Carcinoma	1	1271	Stage I	T1	NotAvailable	NotAvailable	Alcohol consumption	NotAvailable	34.64177538	14.51388412	2.425484574	2.271759246
TCGA-PD-A5DF	FEMALE	58	Hepatocellular Carcinoma	1	639	Stage IIIB	T4	NotAvailable	NotAvailable	No History of Primary Risk Factors	NotAvailable	51.58443238	11.49532168	4.806020378	4.50532432
TCGA-DD-AACF	MALE	68	Hepatocellular Carcinoma	1	365	Stage I	T1	None	6 - Established Cirrhosis	Hepatitis C	NotAvailable	40.5850971	4.093340471	0.7235322	1.233982504
TCGA-BC-A69I	MALE	69	Hepatocellular Carcinoma	0	387	Stage I	T1	None	NotAvailable	No History of Primary Risk Factors	NotAvailable	58.03627745	6.86136934	2.527184284	1.545488215
TCGA-ZP-A9D4	FEMALE	64	Hepatocellular Carcinoma	0	395	NotAvailable	T1	None	0 - No Fibrosis	No History of Primary Risk Factors	NotAvailable	85.6673349	23.99595693	2.660595792	6.833362029
TCGA-FV-A2QR	MALE	75	Hepatocellular Carcinoma	1	581	Stage I	T1	NotAvailable	NotAvailable	Other	NotAvailable	179.8452452	29.92853388	0.545774422	4.324096361
TCGA-DD-A11C	MALE	69	Hepatocellular Carcinoma	0	662	Stage I	T1	None	0 - No Fibrosis	NotAvailable	Hepatitis B Surface Antigen	41.55265831	11.9575257	1.567561013	1.994119502
TCGA-FV-A4ZO	MALE	52	Hepatocellular Carcinoma	0	12	Stage I	T1	None	NotAvailable	Hepatitis C	Hepatitis C Virus RNA	110.136577	30.56297335	12.85758501	5.954798723
TCGA-DD-AAEK	MALE	51	Hepatocellular Carcinoma	0	1067	Stage II	T2	None	6 - Established Cirrhosis	Hepatitis B	NotAvailable	274.3637837	26.35411551	4.078058299	4.885526949
TCGA-DD-AAE7	MALE	72	Hepatocellular Carcinoma	0	644	Stage I	T1	None	3,4 - Fibrous Speta	Alcohol consumption	NotAvailable	10.90129429	7.248101415	2.308531417	1.037988971
TCGA-DD-AAW3	MALE	69	Hepatocellular Carcinoma	0	1633	Stage I	T1	None	0 - No Fibrosis	No History of Primary Risk Factors	NotAvailable	69.76200786	29.10505955	3.736415175	4.107677555
TCGA-WQ-A9G7	FEMALE	71	Hepatocellular Carcinoma	0	30	NotAvailable	T3a	None	NotAvailable	No History of Primary Risk Factors	NotAvailable	87.92366374	62.82614167	4.165640719	8.978180208
TCGA-CC-A8HT	MALE	74	Hepatocellular Carcinoma	1	140	Stage IIIA	T3	NotAvailable	NotAvailable	Alcohol consumption	Hepatitis C Antibody	55.21794444	14.76417941	7.13487031	5.190492573
TCGA-DD-A4ND	FEMALE	56	Hepatocellular Carcinoma	0	2746	Stage I	T1	None	0 - No Fibrosis	No History of Primary Risk Factors	NotAvailable	160.4854831	44.79147192	4.780462753	5.272453702
TCGA-CC-A1HT	MALE	50	Hepatocellular Carcinoma	1	101	Stage IIIA	T3	NotAvailable	NotAvailable	No History of Primary Risk Factors	Hepatitis C Antibody	32.62391736	13.72724962	3.576905346	7.416743244
TCGA-DD-A3A9	FEMALE	64	Hepatocellular Carcinoma	1	931	Stage IVB	T4	None	0 - No Fibrosis	Other	NotAvailable	30.1890054	30.91599893	2.83514089	3.159928132

TCGA-ED-A7XP	FEMALE		53	Hepatocellular Carcinoma	0	400	Stage II	T2	Micro	NotAvailable	No History of Primary Risk Factors	Hepatitis B Surface Antigen	35.64054918	10.263986	3.223364835	2.790551453
TCGA-G3-A7M6	FEMALE		60	Hepatocellular Carcinoma	0	632	Stage I	T1	None	1,2 - Portal Fibrosis	Hepatitis C	NotAvailable	33.60391377	12.9661357	8.503783012	5.721688291
TCGA-DD-AADF	FEMALE		64	Hepatocellular Carcinoma	1	115	Stage I	T1	None	NotAvailable	Hepatitis B	NotAvailable	49.5328932	19.54496625	1.492617659	0.974220846
TCGA-FV-A2QQ	MALE		80	Hepatocellular Carcinoma	0	729	Stage I	T1	None	NotAvailable	Alcohol consumption	NotAvailable	38.56819904	15.48962751	3.309940275	2.2980919
TCGA-ZP-A9CY	FEMALE		66	Hepatocellular Carcinoma	0	782	NotAvailable	T1	None	6 - Established Cirrhosis	Non-Alcoholic Fatty Liver Disease	NotAvailable	54.45559309	25.92447548	2.176435128	2.661806762
TCGA-DD-AADJ	FEMALE		70	Hepatocellular Carcinoma	0	1066	Stage I	T1	None	NotAvailable	NotAvailable	NotAvailable	55.29889873	57.41362016	3.464909143	3.217550195
TCGA-DD-AACS	MALE		39	Hepatocellular Carcinoma	0	1804	Stage I	T1	None	NotAvailable	Hepatitis B	NotAvailable	408.6402122	84.09066544	2.762831373	4.96538461
TCGA-DD-A4NJ	FEMALE		54	Hepatocellular Carcinoma	0	928	Stage II	T2	None	1,2 - Portal Fibrosis	Non-Alcoholic Fatty Liver Disease	Hepatitis B Surface Antigen	107.3294151	8.662809341	1.819598365	8.15088811
TCGA-GJ-A9DB	MALE		68	Hepatocellular Carcinoma	1	67	Stage I	T1	None	NotAvailable	No History of Primary Risk Factors	NotAvailable	80.21766099	20.71122322	2.812368687	4.485808446
TCGA-DD-AACB	FEMALE		74	Hepatocellular Carcinoma	0	2324	Stage I	T1	Micro	6 - Established Cirrhosis	Hepatitis B	NotAvailable	31.03942867	21.90131408	5.889517769	2.412580492
TCGA-2V-A95S	MALE	NotAvailable		Hepatocellular Carcinoma	0	NotAvaila	Stage II	T2	Micro	6 - Established Cirrhosis	Other	NotAvailable	60.2237991	46.09881458	8.500983844	3.658097649
TCGA-ED-A459	MALE		47	Hepatocellular Carcinoma	0	910	Stage II	T2	Micro	NotAvailable	NotAvailable	Hepatitis B Surface Antigen	63.32819473	42.47811955	13.76195716	4.795209646
TCGA-MI-A75H	MALE		77	Hepatocellular Carcinoma	0	747	NotAvailable	NotAvailable	Micro	6 - Established Cirrhosis	Hepatitis C	HCV Genotype	33.73881428	6.614166144	3.923277482	1.59080586
TCGA-DD-AACQ	MALE		50	Hepatocellular Carcinoma	1	432	Stage II	T2	None	NotAvailable	Hepatitis B	NotAvailable	217.4578804	90.26693263	2.282000658	5.281190662
TCGA-G3-A25X	MALE		73	Hepatocellular Carcinoma	0	1779	Stage II	T2	None	1,2 - Portal Fibrosis	Hepatitis B	NotAvailable	10.37390723	13.31918748	1.361225827	2.63982402
TCGA-DD-AACZ	FEMALE		63	Hepatocellular Carcinoma	1	171	Stage I	T1	Macro	NotAvailable	NotAvailable	NotAvailable	293.6570647	68.82404257	21.43795095	12.48052214
TCGA-ZS-A9CE	FEMALE		79	Hepatocellular Carcinoma	0	1241	Stage II	T2	Micro	0 - No Fibrosis	NotAvailable	Hepatitis C Antibody	134.6956032	7.978296035	0.320795758	1.959726155
TCGA-LG-A9QD	MALE		68	Hepatocellular Carcinoma	0	366	Stage IIIA	T3a	None	NotAvailable	No History of Primary Risk Factors	NotAvailable	39.85410446	21.49984847	3.244306786	1.734402841
TCGA-DD-AAVP	MALE		48	Hepatocellular Carcinoma	0	2752	Stage I	T1	None	6 - Established Cirrhosis	Hepatitis B	NotAvailable	105.4434865	8.820539422	3.438179681	2.09682591
TCGA-BW-A5NO	MALE		50	Hepatocellular Carcinoma	0	20	Stage IIIA	T3a	None	3,4 - Fibrous Speta	Hepatitis C	NotAvailable	118.4495983	16.19240409	5.97655406	5.780826008
TCGA-G3-A3CH	MALE		53	Hepatocellular Carcinoma	0	780	Stage IIIA	T3a	None	6 - Established Cirrhosis	Hepatitis B	NotAvailable	43.77876416	6.955590044	1.438079436	3.153686029
TCGA-DD-AADN	MALE		59	Hepatocellular Carcinoma	0	898	Stage I	T1	Micro	NotAvailable	Alcohol consumption	NotAvailable	140.9267061	28.38084853	11.26167576	3.299471576
TCGA-CC-A5UE	MALE		48	Hepatocellular Carcinoma	1	272	Stage IIIB	T4	NotAvailable	NotAvailable	Alcohol consumption	Hepatitis C Antibody	122.4294361	40.69655053	14.91296259	5.094792483
TCGA-DD-AACL	FEMALE		66	Hepatocellular Carcinoma	1	107	Stage I	T1	None	NotAvailable	Hepatitis C	NotAvailable	122.3638785	21.36575212	10.72592292	5.616332916
TCGA-2Y-A9H1	MALE		58	Hepatocellular Carcinoma	1	1229	Stage I	T1	NotAvailable	1,2 - Portal Fibrosis	Alcohol consumption	Hepatitis C Antibody	39.62069251	30.46595112	1.535581289	1.937251956
TCGA-DD-AACU	MALE		59	Hepatocellular Carcinoma	0	1567	Stage I	T1	None	NotAvailable	Hepatitis B	NotAvailable	44.31382225	33.16533441	8.36980364	2.940005689
TCGA-2Y-A9GV	FEMALE		54	Hepatocellular Carcinoma	1	2532	Stage I	T1	NotAvailable	NotAvailable	Alcohol consumption	Hepatitis C Antibody	173.1542765	13.93745299	2.461680226	2.339694565
TCGA-DD-AADK	FEMALE		68	Hepatocellular Carcinoma	0	1049	Stage II	T2	Micro	6 - Established Cirrhosis	Hepatitis B	NotAvailable	50.77906557	19.6810922	1.56462182	3.309460907
TCGA-BC-4072	FEMALE		74	Hepatocellular Carcinoma	1	1490	Stage IIIA	T3	Macro	NotAvailable	No History of Primary Risk Factors	NotAvailable	26.19817708	18.12193184	3.584001556	5.3826676
TCGA-DD-AAVR	MALE		44	Hepatocellular Carcinoma	0	2513	Stage I	T1	None	6 - Established Cirrhosis	Hepatitis B	NotAvailable	7.744355816	10.52354718	4.690963428	1.259310537
TCGA-RC-A7SH	MALE		42	Hepatocellular Carcinoma	0	468	Stage II	T2	Micro	3,4 - Fibrous Speta	Hepatitis B	NotAvailable	192.6492615	45.25693712	4.070914815	3.559663052
TCGA-G3-AAV7	MALE		38	Hepatocellular Carcinoma	0	361	Stage II	T2	Micro	5 - Nodular Formation and Incomplete Cirrho	Hepatitis B	Hepatitis B Surface Antigen	106.8044246	44.03867044	4.878304096	5.886164397
TCGA-DD-AACJ	MALE		75	Hepatocellular Carcinoma	0	2102	Stage II	T2	Micro	NotAvailable	NotAvailable	NotAvailable	89.46705523	11.05756037	3.570381319	8.113809877
TCGA-CC-5264	MALE		71	Hepatocellular Carcinoma	1	102	Stage IIIA	T3	None	NotAvailable	NotAvailable	Hepatitis C Antibody	63.66102835	32.90913141	5.974803858	6.694134318
TCGA-ES-A2HS	MALE		80	Hepatocellular Carcinoma	1	688	Stage I	T1	None	0 - No Fibrosis	No History of Primary Risk Factors	NotAvailable	10.86727474	37.82148528	4.184989341	1.914879785
TCGA-EP-A12J	MALE		62	Hepatocellular Carcinoma	0	570	Stage I	T1	None	NotAvailable	Hepatitis C	NotAvailable	53.18848029	25.55438145	8.296543295	4.453507434
TCGA-CC-A7IH	MALE		58	Hepatocellular Carcinoma	0	365	Stage IIIA	T3	NotAvailable	NotAvailable	Alcohol consumption	Hepatitis C Antibody	52.35567593	9.454729837	3.506751884	2.630128985
TCGA-RC-A6M3	MALE		24	Hepatocellular Carcinoma	0	0	Stage II	T2	Micro	NotAvailable	NotAvailable	NotAvailable	23.28338654	24.05675741	2.10800764	4.35510046
TCGA-CC-5262	MALE		67	Hepatocellular Carcinoma	1	103	Stage IIIC	T4	NotAvailable	NotAvailable	No History of Primary Risk Factors	Hepatitis C Antibody	164.6504411	33.37674152	5.158298356	4.132235679
TCGA-DD-A114	MALE		42	Hepatocellular Carcinoma	1	1149	Stage II	T2	Micro	5 - Nodular Formation and Incomplete Cirrho	Hepatitis C	Hepatitis C Virus RNA	102.5492797	16.0029413	2.464405697	3.608413625
TCGA-DD-AADD	MALE		51	Hepatocellular Carcinoma	0	1231	Stage I	T1	None	NotAvailable	Hepatitis B	NotAvailable	275.510312	42.79741474	12.72404637	11.04603212
TCGA-2Y-A9H0	MALE		49	Hepatocellular Carcinoma	0	3675	Stage IIIA	T3	NotAvailable	NotAvailable	Alcohol consumption	NotAvailable	83.04038468	20.61132463	2.720367709	3.392066983
TCGA-DD-A1EL	MALE		23	Hepatocellular Carcinoma	1	415	Stage II	T2	None	0 - No Fibrosis	Hepatitis B	Hepatitis B Surface Antigen	44.45905908	8.632217834	7.974559241	6.730572247
TCGA-DD-A39V	MALE		77	Hepatocellular Carcinoma	1	643	Stage II	T2	None	0 - No Fibrosis	No History of Primary Risk Factors	NotAvailable	8.661050466	5.139731213	7.409190061	1.864677174
TCGA-ED-A5KG	FEMALE		60	Hepatocellular Carcinoma	0	854	Stage II	T2	Micro	NotAvailable	No History of Primary Risk Factors	Hepatitis B Surface Antigen	51.8344541	18.57790416	5.128315702	3.42355759
TCGA-DD-A4NV	MALE		61	Hepatocellular Carcinoma	0	2398	Stage IIIA	T3	None	0 - No Fibrosis	Non-Alcoholic Fatty Liver Disease	Hepatitis B Surface Antigen	454.0000166	15.91643957	3.654609681	3.48491532
TCGA-DD-AAE2	MALE		51	Hepatocellular Carcinoma	0	638	Stage I	T1	None	6 - Established Cirrhosis	Hepatitis B	NotAvailable	246.6385523	16.3357645	3.271827228	3.270711011
TCGA-DD-A4NL	MALE		46	Hepatocellular Carcinoma	0	1711	Stage I	T1	None	0 - No Fibrosis	Alcohol consumption	Hepatitis B Surface Antigen	47.1195854	27.875703	3.589342469	2.125143177
TCGA-DD-A3A1	MALE		65	Hepatocellular Carcinoma	1	233	Stage IIIA	T3b	Macro	0 - No Fibrosis	No History of Primary Risk Factors	NotAvailable	82.88855091	48.24157982	2.059333005	4.038486365
TCGA-DD-A73A	MALE		71	Hepatocellular Carcinoma	0	728	Stage I	T1	None	3,4 - Fibrous Speta	Alcohol consumption	Hepatitis B Surface Antigen	58.36501265	31.17096798	4.168904839	3.579579729
TCGA-CC-A7IF	MALE		59	Hepatocellular Carcinoma	1	649	Stage IIIA	T3	NotAvailable	NotAvailable	Alcohol consumption	Hepatitis C Antibody	68.41921755	5.013834472	1.80324018	3.570664224
TCGA-DD-A73F	FEMALE		77	Hepatocellular Carcinoma	0	1085	Stage I	T1	None	0 - No Fibrosis	No History of Primary Risk Factors	NotAvailable	128.2692524	24.96227618	2.20588942	5.306697264
TCGA-G3-A7M9	MALE		70	Hepatocellular Carcinoma	1	56	Stage IIIB	T3b	Macro	6 - Established Cirrhosis	Hemochromatosis	Hepatitis C Antibody	57.0626093	12.76573097	12.60379679	5.920305874
TCGA-DD-A1EF	FEMALE		57	Hepatocellular Carcinoma	1	394	Stage I	T1	None	3,4 - Fibrous Speta	No History of Primary Risk Factors	Hepatitis B Surface Antigen	29.33255981	12.56949509	23.12914344	3.723312112
TCGA-DD-A4NG	MALE		77	Hepatocellular Carcinoma	1	802	Stage IIIA	T3a	Micro	NotAvailable	Alcohol consumption	Hepatitis B Surface Antigen	105.1912799	27.36019748	7.312236641	4.835965457
TCGA-DD-AADC	MALE		53	Hepatocellular Carcinoma	1	425	Stage I	T1	Macro	NotAvailable	Hepatitis B	NotAvailable	111.115912	25.40459636	9.398126706	3.526943737
TCGA-DD-A1EH	MALE		23	Hepatocellular Carcinoma	0	1495	Stage III	T3	Macro	3,4 - Fibrous Speta	Hepatitis B	Hepatitis B Surface Antigen	123.0620211	34.93840124	6.758114407	5.958636403
TCGA-CC-A3M9	MALE		45	Hepatocellular Carcinoma	1	300	Stage IIIA	T3	NotAvailable	NotAvailable	Alcohol consumption	Hepatitis C Antibody	12.51887873	18.24087687	9.548144473	2.382155337
TCGA-DD-AAD1	FEMALE		51	Hepatocellular Carcinoma	0	564	Stage I	T1	None	NotAvailable	Hepatitis B	NotAvailable	87.95637512	31.86566	2.632586593	5.915725821
TCGA-DD-AAE6	FEMALE		59	Hepatocellular Carcinoma	0	141	Stage I	T1	Micro	6 - Established Cirrhosis	NotAvailable	NotAvailable	133.6058728	80.17778918	9.669875001	5.210496211
TCGA-MI-A75G	MALE		63	Hepatocellular Carcinoma	0	698	Stage II	T2	None	6 - Established Cirrhosis	Alcohol consumption	HCV Genotype	317.3240035	175.9084908	5.206287872	2.644820116
TCGA-ED-A7XO	MALE		29	Hepatocellular Carcinoma	0	427	Stage IIIA	T3a	Micro	NotAvailable	No History of Primary Risk Factors	Hepatitis C Antibody	29.86465449	6.937619317	1.862672971	1.395479541
TCGA-DD-AAW0	MALE		54	Hepatocellular Carcinoma	0	2015	Stage I	T1	None	3,4 - Fibrous Speta	Hepatitis B	NotAvailable	92.72983606	14.75119788	2.926219257	2.721700008
TCGA-G3-AAV4	FEMALE		83	Hepatocellular Carcinoma	1	27	Stage I	T1	None	3,4 - Fibrous Speta	Hepatitis B	NotAvailable	61.13105925	15.72341021	6.603662432	3.366424235
TCGA-UB-A7MF	MALE		56	Hepatocellular Carcinoma	1	214	Stage IIIA	T3a	Micro	6 - Established Cirrhosis	Hepatitis B	Hepatitis C Antibody	29.98603129	38.27957277	3.134923036	4.071943597
TCGA-ED-A66X	MALE		35	Hepatocellular Carcinoma	0	406	Stage IIIA	T3a	Micro	NotAvailable	Alcohol consumption	Hepatitis B Surface Antigen	179.6117372	23.81663371	6.613418521	4.41439382
TCGA-5R-AAAM	FEMALE		65	Hepatocellular Carcinoma	1	46	Stage II	T2	Micro	6 - Established Cirrhosis	No History of Primary Risk Factors	Hepatitis C Antibody	40.78570908	11.09734557	3.397863446	2.87683988
TCGA-DD-AACC	MALE		61	Hepatocellular Carcinoma	1	1685	Stage I	T1	None	6 - Established Cirrhosis	Hepatitis B	NotAvailable	34.70702032	15.0000639	4.658065278	1.744236543
TCGA-DD-AADY	FEMALE		55	Hepatocellular Carcinoma	0	555	Stage I	T1	None	6 - Established Cirrhosis	Hepatitis B	NotAvailable	76.65406344	61.0823058	1.812014788	4.429060475
TCGA-FV-A23B	FEMALE		70	Hepatocellular Carcinoma	1	1852	Stage II	T2	NotAvailable	NotAvailable	Hepatitis C	NotAvailable	50.68394213	20.70666467	10.92522691	5.702100166
TCGA-DD-AADA	FEMALE		66	Hepatocellular Carcinoma	0	1233	Stage I	T1	Macro	NotAvailable	Hepatitis B	NotAvailable	13.52638145	8.319093675	2.939794695	0.902240475
TCGA-DD-AAC9	MALE		51	Hepatocellular Carcinoma	0	347	Stage I	T1	None	6 - Established Cirrhosis	Hepatitis B	NotAvailable	56.56278175	16.3942853	2.371094789	3.152828773
TCGA-XR-A8TD	FEMALE		49	Hepatocellular Carcinoma	0	1030	Stage IIIB	T3	None	0 - No Fibrosis	No History of Primary Risk Factors	NotAvailable	351.0009255	45.57549305	2.056007104	3.299524025
TCGA-BC-A10S	MALE		81	Hepatocellular Carcinoma	1	1423	NotAvailable	T3	Micro	NotAvailable	Alcohol consumption	NotAvailable	16.47983223	14.93100951	5.331846785	2.04537753
TCGA-XR-A8TE	MALE		16	Hepatocellular Carcinoma	0	925	Stage IIIA	T3	None	0 - No Fibrosis	No History of Primary Risk Factors	NotAvailable	119.7188828	26.82320263	4.771268663	4.338881566
TCGA-DD-AAEB	MALE		60	Hepatocellular Carcinoma	0	478	Stage I	T1	None	NotAvailable	Alcohol consumption	NotAvailable	27.06143253	4.405713605	3.104127238	1.468034045
TCGA-LG-A6GG	FEMALE		79	Hepatocellular Carcinoma	0	387	Stage II	T2	Micro	NotAvailable	Other	NotAvailable	40.03513455	26.47285727	6.934768164	2.362605067
TCGA-BC-A10R	FEMALE		66	Hepatocellular Carcinoma	1	308	NotAvailable	T3	Micro	NotAvailable	No History of Primary Risk Factors	NotAvailable	32.65685269	34.4032877	2.960218666	1.985646012
TCGA-2Y-A9H5	FEMALE		59	Hepatocellular Carcinoma	1	555	Stage I	T1	None	1,2 - Portal Fibrosis	Alcohol consumption	Hepatitis C Antibody	82.77895557	27.63646264	3.750127808	3.369707914
TCGA-DD-A4NP	MALE		32	Hepatocellular Carcinoma	0	3308	Stage I	T1	None	0 - No Fibrosis	No History of Primary Risk Factors	Hepatitis B Surface Antigen	47.08663945	13.8758756	2.932378518	2.103589802
TCGA-CC-A7IK	MALE		59	Hepatocellular Carcinoma	1	262	Stage IIIA	T3	NotAvailable	NotAvailable	Alcohol consumption	Hepatitis C Antibody	123.7561075	8.521139091	10.81419273	8.58132072
TCGA-DD-A4NN	FEMALE		56	Hepatocellular Carcinoma	1	899	Stage I	T1	None	0 - No Fibrosis	No History of Primary Risk Factors	NotAvailable	61.24232012	17.25831047	2.898827042	2.336459512
TCGA-G3-A7M5	MALE		76	Hepatocellular Carcinoma	0	447	Stage I	T1	None	0 - No Fibrosis	Alcohol consumption	Hepatitis C Antibody	136.4911631	3.258287589	1.555516943	1.287162383
TCGA-DD-A3A2	FEMALE		76	Hepatocellular Carcinoma	1	2131	Stage I	T1	None	0 - No Fibrosis	No History of Primary Risk Factors	NotAvailable	117.8359741	10.5066399	2.394750427	3.195960277
TCGA-DD-AAW1	MALE		55	Hepatocellular Carcinoma	0	1989	Stage IIIA	T3	Micro	6 - Established Cirrhosis	No History of Primary Risk Factors	NotAvailable	60.52690593	20.95123035	5.380180246	4.462527526
TCGA-BC-4073	MALE		73	Hepatocellular Carcinoma	0	849	Stage IIIA	T3	Macro	NotAvailable	Hemochromatosis	NotAvailable	172.2786531	70.93281062	8.906923239	9.326019521
TCGA-DD-AAD0	FEMALE		73	Hepatocellular Carcinoma	0	137	Stage I	T1	None	NotAvailable	Hepatitis B	NotAvailable	135.6281997	23.10309257	4.148754266	3.12617131
TCGA-DD-A4NO	MALE		65	Hepatocellular Carcinoma	0	2245	Stage I	T1	None	0 - No Fibrosis	Hepatitis C	Hepatitis C Virus RNA	105.8240857	8.562818463	2.679466137	4.017252919
TCGA-EP-A3RK	MALE		73	Hepatocellular Carcinoma	0	363	Stage IIIA	T3a	NotAvailable	1,2 - Portal Fibrosis	Alcohol consumption	NotAvailable	51.86710619	8.545199024	4.278850581	3.615233383
TCGA-DD-AAVV	MALE		56	Hepatocellular Carcinoma	0	2455	Stage II	T2	Micro	3,4 - Fibrous Speta	Hepatitis B	NotAvailable	50.7453905	21.01785196	3.344908299	2.589705549
TCGA-BC-A8YO	FEMALE		66	Hepatocellular Carcinoma	0	562	Stage IIIC	T4	Macro	NotAvailable	Alcohol consumption	NotAvailable	56.15755593	16.94970941	3.372039899	5.715604484
TCGA-DD-A4NQ	MALE		60	Hepatocellular Carcinoma	1	373	Stage II	T2	Macro	NotAvailable	Hepatitis B	Hepatitis C Virus RNA	184.2231051	42.02585505	3.485279411	9.275415967
TCGA-DD-AACE	MALE		62	Hepatocellular Carcinoma	0	2184	Stage I	T1	None	6 - Established Cirrhosis	Hepatitis B	NotAvailable	78.03995486	7.451054094	6.56507473	2.939735613
TCGA-DD-AACD	MALE		48	Hepatocellular Carcinoma	1	381	Stage I	T1	Micro	1,2 - Portal Fibrosis	Hepatitis B	NotAvailable	22.11687848	7.774573354	2.043352897	2.001744438
TCGA-2Y-A9GY	FEMALE		64	Hepatocellular Carcinoma	1	757	Stage II	T2	NotAvailable	NotAvailable	No History of Primary Risk Factors	NotAvailable	45.46071016	25.49030247	9.634418593	4.328909992
TCGA-BC-A3KG	FEMALE		68	Hepatocellular Carcinoma	0	680	Stage II	T2	Micro	NotAvailable	Alcohol consumption	NotAvailable	219.7612963	15.98237237	13.27370644	6.327459563
TCGA-CC-A9FV	MALE		57	Hepatocellular Carcinoma	0	0	Stage IIIA	T3	NotAvailable	NotAvailable	Alcohol consumption	Hepatitis C Antibody	7.271537814	9.373383224	22.6032698	1.806409358
TCGA-DD-A3A8	MALE		75	Hepatocellular Carcinoma	1	11	Stage II	T2	None	0 - No Fibrosis	No History of Primary Risk Factors	Hepatitis B Surface Antigen	59.41352446	12.69470208	2.54449943	2.511960073
TCGA-G3-AAV6	FEMALE		53	Hepatocellular Carcinoma	1	65	Stage IIIA	T3a	Micro	0 - No Fibrosis	No History of Primary Risk Factors	Hepatitis B Surface Antigen	77.91199587	25.60160645	8.185790793	2.922284136
TCGA-ES-A2HT	MALE		54	Hepatocellular Carcinoma	1	438	Stage I	T1	None	0 - No Fibrosis	Hepatitis C	NotAvailable	29.41584572	29.86534411	0.601138464	1.684021587
TCGA-XR-A8TC	FEMALE		43	Hepatocellular Carcinoma	0	1339	Stage I	T1	None	6 - Established Cirrhosis	Hepatitis B	NotAvailable	65.80291304	9.998084893	2.65018227	3.319410339
TCGA-2Y-A9H3	MALE		45	Hepatocellular Carcinoma	0	1516	Stage II	T2	Micro	1,2 - Portal Fibrosis	Alcohol consumption	Hepatitis C Antibody	9.9427272	12.39870232	2.868148414	1.001086034
TCGA-DD-AADR	MALE		58	Hepatocellular Carcinoma	0	2028	Stage I	T1	None	6 - Established Cirrhosis	Alcohol consumption	NotAvailable	19.60064348	15.58659509	7.174082347	3.38387216
TCGA-CC-A7IG	MALE		47	Hepatocellular Carcinoma	1	299	Stage II	T2	NotAvailable	NotAvailable	Alcohol consumption	Hepatitis C Antibody	179.7765947	75.57058812	11.98105806	4.366017857
TCGA-RC-A6M6	MALE		75	Hepatocellular Carcinoma	0	9	Stage II	T2	None	NotAvailable	Alcohol consumption	NotAvailable	54.97310336	8.934035178	17.3622309	4.223766035
TCGA-DD-A1EG	MALE		76	Hepatocellular Carcinoma	1	1372	Stage I	T1	None	1,2 - Portal Fibrosis	Alcohol consumption	Hepatitis B Surface Antigen	52.36435474	17.43668564	4.979361875	3.341142142
TCGA-DD-AADM	MALE		58	Hepatocellular Carcinoma	1	12	Stage II	T2	None	NotAvailable	Alcohol consumption	NotAvailable	27.83579172	17.14500919	1.890987138	1.920925528
TCGA-DD-A73C	FEMALE		65	Hepatocellular Carcinoma	0	701	Stage IIIA	T3a	None	0 - No Fibrosis	No History of Primary Risk Factors	Hepatitis B Surface Antigen	237.5359837	7.586085991	3.787100048	5.874619246
TCGA-G3-A7M8	MALE		31	Hepatocellular Carcinoma	0	430	Stage I	T1	None	6 - Established Cirrhosis	Alcohol consumption	Hepatitis C Antibody	8.215694013	4.199130344	0.969059785	1.651170104
TCGA-CC-A7IL	MALE		61	Hepatocellular Carcinoma	1	278	Stage IIIA	T3	NotAvailable	NotAvailable	Alcohol consumption	Hepatitis C Antibody	226.4494305	17.50475891	2.623365917	11.45662986
TCGA-GJ-A6C0	FEMALE		75	Hepatocellular Carcinoma	1	31	Stage II	T2	Micro	NotAvailable	No History of Primary Risk Factors	NotAvailable	158.2532109	32.65835269	3.458480949	1.838142876
TCGA-DD-AAEI	MALE		72	Hepatocellular Carcinoma	0	1531	Stage I	T1	None	6 - Established Cirrhosis	Hepatitis B	NotAvailable	64.36096458	5.46258697	2.727351907	1.829459639
TCGA-DD-AACK	MALE		70	Hepatocellular Carcinoma	0	9	Stage I	T1	Micro	6 - Established Cirrhosis	Hepatitis B	NotAvailable	183.5277152	35.2808815	5.75003522	6.308846841
TCGA-BC-A10Y	MALE		76	Hepatocellular Carcinoma	1	711	NotAvailable	T4	Micro	NotAvailable	Alcohol consumption	NotAvailable	37.11127532	5.564391013	1.031471427	2.057512206
TCGA-G3-A25S	MALE		64	Hepatocellular Carcinoma	1	416	Stage I	T1	Micro	6 - Established Cirrhosis	Alcohol consumption	NotAvailable	33.93264794	18.16174111	7.892510267	2.393192961
TCGA-WQ-AB4B	MALE		62	Hepatocellular Carcinoma	0	395	Stage II	T2	Micro	NotAvailable	Alcohol consumption	NotAvailable	14.01291192	8.431351518	3.168827702	1.534235598
TCGA-DD-A4NH	FEMALE		65	Hepatocellular Carcinoma	0	917	Stage IIIB	T3b	Macro	1,2 - Portal Fibrosis	Alcohol consumption	Hepatitis B Surface Antigen	67.57764516	18.73056935	5.942659327	3.879539714
TCGA-WX-AA44	FEMALE		64	Hepatocellular Carcinoma	0	615	Stage I	T1	None	6 - Established Cirrhosis	Non-Alcoholic Fatty Liver Disease	Hepatitis C Antibody	35.74759224	16.49625628	2.186843892	3.68234806
TCGA-RG-A7D4	MALE		69	Hepatocellular Carcinoma	0	1098	Stage II	T2	NotAvailable	NotAvailable	Hepatitis C	Hepatitis C Antibody	58.4577994	8.222505363	10.89771786	7.710954959
TCGA-DD-AAE3	MALE		50	Hepatocellular Carcinoma	0	566	Stage I	T1	None	1,2 - Portal Fibrosis	Alcohol consumption	NotAvailable	9.712481334	5.732129892	4.766461428	0.839388954
TCGA-DD-AAE9	MALE		69	Hepatocellular Carcinoma	0	722	Stage I	T1	None	1,2 - Portal Fibrosis	Alcohol consumption	NotAvailable	32.5138205	7.647208798	11.42778625	1.850685567
TCGA-RC-A7SK	MALE		59	Hepatocellular Carcinoma	0	472	Stage I	T1	Micro	1,2 - Portal Fibrosis	Hepatitis C	NotAvailable	38.27241917	14.12737358	3.021037412	2.777680829
TCGA-DD-AAVZ	MALE		38	Hepatocellular Carcinoma	0	1900	Stage I	T1	None	6 - Established Cirrhosis	Hepatitis B	NotAvailable	62.93784594	20.849151	7.922898882	3.27377346
TCGA-G3-AAUZ	MALE		48	Hepatocellular Carcinoma	0	480	Stage I	T1	None	1,2 - Portal Fibrosis	Hepatitis B	Hepatitis C Antibody	18.53301925	4.258976939	0.5116102	2.186320322
TCGA-UB-A7MD	MALE		67	Hepatocellular Carcinoma	1	52	Stage I	T1	Micro	6 - Established Cirrhosis	Alcohol consumption	Hepatitis C Antibody	61.6625228	7.390882671	5.922962419	2.488767735
TCGA-MI-A75C	MALE		64	Hepatocellular Carcinoma	0	291	Stage I	T1	None	NotAvailable	Hepatitis C	HBV Surface Antibody	63.74571257	70.15869963	3.756992099	3.764559076
TCGA-CC-A5UD	MALE		45	Hepatocellular Carcinoma	1	304	Stage IIIA	T3	NotAvailable	NotAvailable	Alcohol consumption	Hepatitis C Antibody	54.35557581	63.10025463	12.04761673	7.07800757
TCGA-ED-A7PY	FEMALE		20	Hepatocellular Carcinoma	0	390	Stage II	T2	Micro	NotAvailable	No History of Primary Risk Factors	Hepatitis B Surface Antigen	22.87971457	25.0985338	4.231924005	1.806584609
TCGA-DD-A4NK	FEMALE		80	Hepatocellular Carcinoma	1	1210	Stage IIIA	T3	None	0 - No Fibrosis	No History of Primary Risk Factors	Hepatitis B Surface Antigen	143.4113918	6.356812583	1.033035009	2.438914152
TCGA-EP-A2KC	MALE		62	Hepatocellular Carcinoma	1	19	Stage I	T1	None	NotAvailable	Hepatitis C	NotAvailable	99.98486277	8.88424776	4.733373142	6.533093477
TCGA-DD-AAD6	MALE		66	Hepatocellular Carcinoma	0	672	Stage IIIA	T3a	None	NotAvailable	Hepatitis B	NotAvailable	84.7011449	25.72715246	14.69678632	8.006447592
TCGA-DD-AACI	MALE		69	Hepatocellular Carcinoma	0	1618	Stage II	T2	None	5 - Nodular Formation and Incomplete Cirrho	NotAvailable	NotAvailable	6.05496454	10.17545167	2.327193589	0.984313229
TCGA-G3-AAV1	MALE		51	Hepatocellular Carcinoma	1	359	Stage IIIC	T4	Micro	6 - Established Cirrhosis	Hepatitis B	Hepatitis C Antibody	65.1534394	7.372136458	6.004651343	5.584956431
TCGA-G3-A5SM	MALE		58	Hepatocellular Carcinoma	0	520	Stage II	T2	None	6 - Established Cirrhosis	Alcohol consumption	NotAvailable	44.65743735	10.86994413	3.1818537	1.649105042
TCGA-DD-A1EJ	FEMALE		71	Hepatocellular Carcinoma	1	1005	Stage IIIC	T1	None	0 - No Fibrosis	No History of Primary Risk Factors	Hepatitis B Surface Antigen	196.6064199	65.64743725	19.57166118	10.64990904
TCGA-DD-AADO	MALE		55	Hepatocellular Carcinoma	0	453	Stage I	T1	Micro	NotAvailable	Alcohol consumption	NotAvailable	86.45697978	24.06875264	5.57257798	3.049488726
TCGA-CC-5258	MALE		48	Hepatocellular Carcinoma	1	129	Stage II	T2	None	NotAvailable	NotAvailable	Hepatitis C Antibody	138.1099645	20.85487555	14.05592295	7.458566297
TCGA-DD-A3A6	FEMALE		72	Hepatocellular Carcinoma	1	3258	Stage II	T2	Micro	0 - No Fibrosis	No History of Primary Risk Factors	Hepatitis B Surface Antigen	5.82791927	5.014243722	64.39303131	0.940745129
TCGA-2Y-A9GX	MALE		68	Hepatocellular Carcinoma	0	2442	Stage I	T1	NotAvailable	NotAvailable	Alcohol consumption	Hepatitis B Surface Antigen	77.99422282	24.44129941	2.41069751	2.96988247
TCGA-DD-A11B	MALE		73	Hepatocellular Carcinoma	1	14	Stage I	T1	None	6 - Established Cirrhosis	Non-Alcoholic Fatty Liver Disease	Hepatitis B Surface Antigen	27.38670205	3.424652504	4.745184239	1.502475647
TCGA-G3-A5SI	MALE		44	Hepatocellular Carcinoma	1	768	Stage II	T2	None	6 - Established Cirrhosis	Hepatitis C	NotAvailable	35.23821592	10.51856337	0.449133161	1.210112035
TCGA-DD-AAVU	MALE		46	Hepatocellular Carcinoma	0	2202	Stage II	T2	Micro	3,4 - Fibrous Speta	Hepatitis B	NotAvailable	21.97164127	7.572154625	7.126979588	2.364329774
TCGA-BC-A112	MALE		80	Hepatocellular Carcinoma	1	153	NotAvailable	T3	None	0 - No Fibrosis	Alcohol consumption	NotAvailable	55.70864601	16.34880512	2.325365836	6.91436767
TCGA-DD-A4NE	FEMALE		75	Hepatocellular Carcinoma	1	660	Stage IIIA	T3a	None	0 - No Fibrosis	No History of Primary Risk Factors	Hepatitis C Virus RNA	137.018847	12.6953084	1.051236579	4.482108729
TCGA-UB-A7ME	MALE		51	Hepatocellular Carcinoma	0	486	Stage I	T1	None	1,2 - Portal Fibrosis	Hepatitis B	Hepatitis B Surface Antigen	35.45277405	17.03431863	5.983884043	2.489490403
TCGA-DD-AADQ	MALE		59	Hepatocellular Carcinoma	0	436	Stage II	T2	None	NotAvailable	Alcohol consumption	NotAvailable	18.17838795	11.81655835	6.832524971	1.622059932
TCGA-DD-AAD3	MALE		43	Hepatocellular Carcinoma	0	1295	Stage I	T1	None	1,2 - Portal Fibrosis	Alcohol consumption	NotAvailable	58.9129694	19.42495075	2.90732453	1.502338749
TCGA-K7-A6G5	MALE		66	Hepatocellular Carcinoma	0	512	Stage I	T1	None	NotAvailable	Alcohol consumption	HCV Genotype	238.5316393	58.16386567	1.88907723	2.179768743
TCGA-DD-A39Z	FEMALE		43	Hepatocellular Carcinoma	1	601	Stage II	T2	None	NotAvailable	Alcohol consumption	NotAvailable	62.21710765	34.3605601	2.574042903	3.137008652
TCGA-DD-AACO	MALE		40	Hepatocellular Carcinoma	0	1876	Stage I	T1	None	6 - Established Cirrhosis	Hepatitis B	NotAvailable	7.622869565	11.24756112	0.842706871	1.845958874
TCGA-DD-AADB	MALE		51	Hepatocellular Carcinoma	0	1242	Stage I	T1	None	NotAvailable	Hepatitis B	NotAvailable	215.9735992	40.75177954	13.90062175	5.229352455
TCGA-CC-5259	FEMALE		60	Hepatocellular Carcinoma	0	250	Stage IIIC	T4	NotAvailable	NotAvailable	No History of Primary Risk Factors	Hepatitis C Antibody	16.35628616	25.18456077	8.897492042	1.309887486
TCGA-FV-A496	FEMALE		84	Hepatocellular Carcinoma	0	10	Stage I	T1	None	0 - No Fibrosis	No History of Primary Risk Factors	NotAvailable	323.442354	49.08917899	2.507103495	5.722974434
TCGA-2Y-A9H8	FEMALE		85	Hepatocellular Carcinoma	1	633	NotAvailable	T1	NotAvailable	NotAvailable	Alcohol consumption	Hepatitis C Virus RNA	130.8545634	23.56848438	4.548001136	2.189085139
TCGA-HP-A5MZ	MALE		78	Hepatocellular Carcinoma	1	91	Stage I	T1	None	NotAvailable	No History of Primary Risk Factors	Hepatitis C Antibody	66.34265366	20.30384517	2.421898438	2.684095033
TCGA-2Y-A9H6	FEMALE		68	Hepatocellular Carcinoma	0	357	Stage I	T1	Macro	NotAvailable	Alcohol consumption	NotAvailable	17.78779157	20.39687671	5.505748866	1.902971851
TCGA-ED-A8O6	FEMALE		50	Hepatocellular Carcinoma	1	56	Stage IIIA	T3a	Micro	NotAvailable	No History of Primary Risk Factors	Hepatitis C Antibody	125.0288171	36.57743402	9.730002951	7.06355762
TCGA-G3-A3CK	MALE		61	Hepatocellular Carcinoma	0	585	Stage I	T1	None	6 - Established Cirrhosis	Hepatitis B	NotAvailable	55.22751152	6.077709657	2.291661747	2.604285439
TCGA-G3-A25U	FEMALE		63	Hepatocellular Carcinoma	0	1636	Stage I	T1	None	0 - No Fibrosis	Hepatitis B	NotAvailable	192.9447089	8.457721204	1.37537594	3.498760574

2. HBV+HCV

barcode	gender	Age at diagnosis	histological type	FU OS status	FU days	pathologic stage	tnm.t	vascular tumor cell type	fibrosis ishak score	hist hepato carc fact	viral hepatitis serology	FAS	SREBF1	PPARG	ACACA
TCGA-DD-AAVX	MALE	38	Hepatocellular Carcinoma	0	1570	Stage II	T2	Micro	6 - Established Cirrhosis	Hepatitis B	NotAvailable	22.35896454	8.210454049	4.283887727	2.327359447
TCGA-DD-AAE0	FEMALE	45	Hepatocellular Carcinoma	0	555	Stage IIIA	T3a	None	3,4 - Fibrous Speta	Hepatitis B	NotAvailable	27.44049215	12.43057362	0.861039863	4.428688781
TCGA-DD-AAE4	FEMALE	49	Hepatocellular Carcinoma	0	608	Stage I	T1	None	6 - Established Cirrhosis	Hepatitis B	NotAvailable	5.011447983	4.40811326	0.845980615	0.808653462
TCGA-DD-AAC8	MALE	72	Hepatocellular Carcinoma	1	16	Stage I	T1	Micro	1,2 - Portal Fibrosis	Hepatitis B	NotAvailable	45.58343487	6.222013285	7.219150775	3.044772198
TCGA-ZP-A9CZ	MALE	72	Hepatocellular Carcinoma	0	706	NotAvailable	T1	None	3,4 - Fibrous Speta	Hepatitis B	Hepatitis B Surface Antiger	111.4998549	14.23879312	1.721384415	6.456417677
TCGA-DD-A1EE	MALE	73	Hepatocellular Carcinoma	1	349	Stage IIIA	T3	None	6 - Established Cirrhosis	Hepatitis C	Hepatitis C Virus RNA	171.2477357	31.56232743	2.818505458	7.212838105
TCGA-G3-A5SK	MALE	58	Hepatocellular Carcinoma	0	744	Stage I	T1	NotAvailable	6 - Established Cirrhosis	Hepatitis C	NotAvailable	28.76042765	20.27476332	1.379266397	0.699957383
TCGA-XR-A8TG	MALE	58	Hepatocellular Carcinoma	0	898	Stage I	T1	None	6 - Established Cirrhosis	Hepatitis C	NotAvailable	29.71127635	18.91190129	5.289780207	3.04193043
TCGA-DD-AAD8	FEMALE	73	Hepatocellular Carcinoma	0	1219	Stage I	T1	None	1,2 - Portal Fibrosis	Hepatitis B	NotAvailable	48.78757385	45.78684926	5.915565217	2.234575807
TCGA-K7-A5RF	MALE	64	Hepatocellular Carcinoma	0	631	Stage I	T1	NotAvailable	NotAvailable	Hepatitis C	Hepatitis C Virus RNA	19.91464746	14.25842509	2.325291372	0.924920034
TCGA-BC-A10W	MALE	50	Hepatocellular Carcinoma	1	91	NotAvailable	T4	NotAvailable	NotAvailable	Hepatitis B	Hepatitis B Surface Antiger	87.94499928	18.69450873	4.811624085	5.337103686
TCGA-DD-AADW	MALE	48	Hepatocellular Carcinoma	0	587	Stage I	T1	None	6 - Established Cirrhosis	Hepatitis B	NotAvailable	262.0518876	21.64545907	3.160424607	5.889634834
TCGA-DD-A1EI	MALE	46	Hepatocellular Carcinoma	0	183	Stage I	T1	None	6 - Established Cirrhosis	Hepatitis B	Hepatitis B Surface Antiger	94.35856415	27.4027725	12.20716007	7.735490162
TCGA-DD-AAEE	MALE	55	Hepatocellular Carcinoma	0	810	Stage I	T1	None	6 - Established Cirrhosis	Hepatitis B	NotAvailable	43.45675107	14.04087585	4.773940965	5.659115523
TCGA-G3-A25Z	MALE	58	Hepatocellular Carcinoma	0	655	Stage I	T1	None	5 - Nodular Formation and Incomplete Cirrhosis	Hepatitis B	NotAvailable	155.4292121	6.432353775	4.536046234	4.065499824
TCGA-RC-A7SB	MALE	53	Hepatocellular Carcinoma	0	588	Stage II	T2	None	0 - No Fibrosis	Hepatitis B	NotAvailable	41.75390313	5.684825968	5.641269466	2.145437565
TCGA-RC-A7SF	MALE	66	Hepatocellular Carcinoma	0	579	Stage I	T1	None	3,4 - Fibrous Speta	Hepatitis C	NotAvailable	287.3975975	70.03215383	1.871034719	3.449339248
TCGA-G3-A25Y	FEMALE	52	Hepatocellular Carcinoma	1	452	Stage I	T1	None	3,4 - Fibrous Speta	Hepatitis B	NotAvailable	36.78101852	8.266749951	3.147699718	2.406178993
TCGA-DD-AAD2	MALE	66	Hepatocellular Carcinoma	0	658	Stage I	T1	Micro	1,2 - Portal Fibrosis	Hepatitis B	NotAvailable	48.88275503	35.62400519	3.899109004	3.563789701
TCGA-DD-AACY	MALE	61	Hepatocellular Carcinoma	0	1450	Stage I	T1	None	3,4 - Fibrous Speta	Hepatitis B	NotAvailable	38.10785097	58.6561023	1.566370128	1.403780067
TCGA-OA-A7B7	MALE	48	Hepatocellular Carcinoma	0	94	Stage II	T2	Micro	0 - No Fibrosis	Hepatitis B	NotAvailable	558.5990297	9.341860448	0.735383118	7.307906855
TCGA-DD-AAVO	MALE	38	Hepatocellular Carcinoma	0	2728	Stage I	T1	None	6 - Established Cirrhosis	Hepatitis B	NotAvailable	67.83390497	18.06255302	3.459593768	2.928457054
TCGA-DD-AADI	FEMALE	43	Hepatocellular Carcinoma	0	1085	Stage I	T1	None	NotAvailable	Hepatitis B	NotAvailable	13.16556356	11.66794234	2.302309092	1.581880434
TCGA-RC-A7S9	FEMALE	47	Hepatocellular Carcinoma	0	640	Stage I	T1	None	6 - Established Cirrhosis	Hepatitis B	NotAvailable	28.17905612	52.23758414	1.667180482	2.776343015
TCGA-3K-AAZ8	MALE	65	Hepatocellular Carcinoma	0	396	Stage IIIB	T3b	NotAvailable	5 - Nodular Formation and Incomplete Cirrhosis	Hepatitis C	NotAvailable	42.17715464	9.989275835	4.237541694	2.892015052
TCGA-MI-A75I	MALE	61	Hepatocellular Carcinoma	0	630	NotAvailable	T2	NotAvailable	NotAvailable	Hepatitis C	NotAvailable	155.1220033	14.44159457	4.951347417	4.785145094
TCGA-DD-A116	MALE	68	Hepatocellular Carcinoma	1	1622	Stage IIIA	T3	NotAvailable	3,4 - Fibrous Speta	Hepatitis B	Hepatitis B Surface Antiger	82.14236987	33.28550893	4.604862931	4.63919003
TCGA-DD-AAVW	MALE	35	Hepatocellular Carcinoma	0	2317	Stage I	T1	None	6 - Established Cirrhosis	Hepatitis B	NotAvailable	41.9977929	12.34449204	4.710399125	2.932025793
TCGA-DD-A1EA	MALE	68	Hepatocellular Carcinoma	0	2415	Stage II	T2	Micro	1,2 - Portal Fibrosis	Hepatitis B	Hepatitis B Surface Antiger	96.00189831	7.629237594	2.630774095	5.450202707
TCGA-BW-A5NP	FEMALE	26	Hepatocellular Carcinoma	0	0	Stage IV	T2	Micro	3,4 - Fibrous Speta	Hepatitis B	NotAvailable	65.60107808	35.17188812	12.33693715	4.092725003
TCGA-DD-AACT	FEMALE	69	Hepatocellular Carcinoma	0	1562	Stage I	T1	None	NotAvailable	Hepatitis B	NotAvailable	37.80510244	8.062792877	2.241004666	1.343692835
TCGA-EP-A2KB	FEMALE	46	Hepatocellular Carcinoma	1	596	Stage I	T1	None	NotAvailable	Hepatitis C	NotAvailable	27.27083342	10.5315698	15.35883156	3.258766832
TCGA-DD-AACA	MALE	65	Hepatocellular Carcinoma	0	2301	Stage I	T1	None	6 - Established Cirrhosis	Hepatitis B	NotAvailable	147.2871873	21.81553581	8.340089259	9.258887475
TCGA-DD-AACG	MALE	52	Hepatocellular Carcinoma	1	469	Stage II	T2	Micro	6 - Established Cirrhosis	Hepatitis B	NotAvailable	28.00462612	13.82738582	11.6920013	4.848896807
TCGA-DD-A119	MALE	40	Hepatocellular Carcinoma	1	223	Stage IV	T3a	Micro	0 - No Fibrosis	Hepatitis B	Hepatitis B Surface Antiger	15.26215757	7.181914193	1.658046016	1.648328917
TCGA-DD-AACH	MALE	69	Hepatocellular Carcinoma	1	195	Stage II	T2	Micro	6 - Established Cirrhosis	Hepatitis B	NotAvailable	79.05129227	15.20669634	8.87366793	4.840465581
TCGA-G3-A3CG	MALE	80	Hepatocellular Carcinoma	0	673	Stage I	T1	Micro	6 - Established Cirrhosis	Hepatitis C	NotAvailable	261.6235756	102.8241115	2.40491564	5.145438345
TCGA-G3-A25T	FEMALE	45	Hepatocellular Carcinoma	0	1553	Stage IIIA	T3	None	0 - No Fibrosis	Hepatitis C	NotAvailable	9.840705427	15.43974363	2.430765624	1.531885334
TCGA-CC-A9FU	FEMALE	52	Hepatocellular Carcinoma	0	0	Stage IIIA	T3a	NotAvailable	NotAvailable	Hepatitis B	Hepatitis C Antibody	128.227866	30.52904403	21.00258977	6.118658936
TCGA-DD-AACN	MALE	32	Hepatocellular Carcinoma	0	1302	Stage I	T1	None	NotAvailable	Hepatitis B	NotAvailable	28.06110146	10.23873081	2.873206573	1.983358972
TCGA-DD-AADP	MALE	45	Hepatocellular Carcinoma	0	458	Stage I	T1	Micro	NotAvailable	Hepatitis B	NotAvailable	171.6408011	63.52753909	3.610401721	6.288990917
TCGA-DD-A73D	FEMALE	68	Hepatocellular Carcinoma	0	693	Stage II	T2	Micro	6 - Established Cirrhosis	Hepatitis C	Hepatitis C Virus RNA	36.62111796	3.321474261	4.871095444	3.766990665
TCGA-FV-A495	FEMALE	51	Hepatocellular Carcinoma	0	1	Stage II	T2	None	3,4 - Fibrous Speta	Hepatitis C	NotAvailable	69.45027742	23.13687422	2.920166597	1.963071364
TCGA-DD-A4NF	MALE	72	Hepatocellular Carcinoma	0	942	Stage I	T1	None	6 - Established Cirrhosis	Hepatitis C	Hepatitis C Virus RNA	38.53690401	11.80172355	4.843333056	5.053814754
TCGA-XR-A8TF	MALE	74	Hepatocellular Carcinoma	1	693	Stage I	T1	None	3,4 - Fibrous Speta	Hepatitis B	NotAvailable	30.52110704	8.266115558	4.50976608	2.120107984
TCGA-DD-AAVS	MALE	56	Hepatocellular Carcinoma	0	1823	Stage I	T1	None	3,4 - Fibrous Speta	Hepatitis B	NotAvailable	33.12415257	21.21615866	4.936105949	2.527253166
TCGA-YA-A8S7	MALE	68	Hepatocellular Carcinoma	1	412	Stage IIIA	T3a	None	NotAvailable	Hepatitis C	NotAvailable	22.81635384	13.87341997	7.75784656	2.827828214
TCGA-G3-A3CJ	MALE	52	Hepatocellular Carcinoma	0	594	Stage II	T2	Micro	5 - Nodular Formation and Incomplete Cirrhosis	Hepatitis C	NotAvailable	54.49914199	12.26989785	2.895612104	2.727803972
TCGA-DD-AAE1	MALE	52	Hepatocellular Carcinoma	0	552	Stage I	T1	None	6 - Established Cirrhosis	Hepatitis B	NotAvailable	48.14240483	43.1189918	10.84294068	4.034345989
TCGA-G3-AAV0	MALE	58	Hepatocellular Carcinoma	0	476	Stage I	T1	None	0 - No Fibrosis	Hepatitis B	Hepatitis C Antibody	68.17217375	40.95374397	2.125075221	1.25569147
TCGA-ZP-A9D2	MALE	51	Hepatocellular Carcinoma	1	765	NotAvailable	T2	Micro	1,2 - Portal Fibrosis	Hepatitis C	Hepatitis C Virus RNA	23.37276315	4.292433642	1.029497401	6.084070048
TCGA-DD-AACF	MALE	68	Hepatocellular Carcinoma	1	365	Stage I	T1	None	6 - Established Cirrhosis	Hepatitis C	NotAvailable	40.5850971	4.093340471	0.7235322	1.233982504
TCGA-FV-A4ZQ	MALE	52	Hepatocellular Carcinoma	0	12	Stage I	T1	None	NotAvailable	Hepatitis C	Hepatitis C Virus RNA	110.136577	30.56297335	12.85758501	5.954798723
TCGA-DD-AAEK	MALE	51	Hepatocellular Carcinoma	0	1067	Stage II	T2	None	6 - Established Cirrhosis	Hepatitis B	NotAvailable	274.3637837	26.35411551	4.078058299	4.885526949
TCGA-G3-A7M6	FEMALE	60	Hepatocellular Carcinoma	0	632	Stage I	T1	None	1,2 - Portal Fibrosis	Hepatitis C	NotAvailable	33.60391377	12.9661357	8.503783012	5.721688291
TCGA-DD-AADF	FEMALE	64	Hepatocellular Carcinoma	1	115	Stage I	T1	None	NotAvailable	Hepatitis B	NotAvailable	49.5328932	19.54496625	1.492617659	0.974220846
TCGA-DD-AACS	MALE	39	Hepatocellular Carcinoma	0	1804	Stage I	T1	None	NotAvailable	Hepatitis B	NotAvailable	408.6402122	84.09066544	2.762831373	4.96538461
TCGA-DD-AACB	FEMALE	74	Hepatocellular Carcinoma	0	2324	Stage I	T1	Micro	6 - Established Cirrhosis	Hepatitis B	NotAvailable	31.03942867	21.90131408	5.889517769	2.412580492
TCGA-MI-A75H	MALE	77	Hepatocellular Carcinoma	0	747	NotAvailable	NotAvaila	Micro	6 - Established Cirrhosis	Hepatitis C	HCV Genotype	33.73881428	6.614166144	3.923277482	1.59080586
TCGA-DD-AACO	MALE	50	Hepatocellular Carcinoma	1	432	Stage II	T2	None	NotAvailable	Hepatitis B	NotAvailable	217.4578804	90.26693263	2.282000658	5.281190662
TCGA-G3-A25X	MALE	73	Hepatocellular Carcinoma	0	1779	Stage II	T2	None	1,2 - Portal Fibrosis	Hepatitis B	NotAvailable	10.37390723	13.31918748	1.361225827	2.63982402
TCGA-DD-AAVP	MALE	48	Hepatocellular Carcinoma	0	2752	Stage I	T1	None	6 - Established Cirrhosis	Hepatitis B	NotAvailable	105.4434865	8.820539422	3.438179681	2.09682591
TCGA-BW-A5NO	MALE	50	Hepatocellular Carcinoma	0	20	Stage IIIA	T3a	None	3,4 - Fibrous Speta	Hepatitis C	NotAvailable	118.4495983	16.19240409	5.97655406	5.780826008
TCGA-G3-A3CH	MALE	53	Hepatocellular Carcinoma	0	780	Stage IIIA	T3a	None	6 - Established Cirrhosis	Hepatitis B	NotAvailable	43.77876416	6.955590044	1.438079436	3.153686029
TCGA-DD-AACL	FEMALE	66	Hepatocellular Carcinoma	1	107	Stage I	T1	None	NotAvailable	Hepatitis C	NotAvailable	122.3638785	21.36575212	10.72592292	5.616332916
TCGA-DD-AACU	MALE	59	Hepatocellular Carcinoma	0	1567	Stage I	T1	None	NotAvailable	Hepatitis B	NotAvailable	44.31382225	33.16533441	8.36980364	2.940005689
TCGA-DD-AADK	FEMALE	68	Hepatocellular Carcinoma	0	1049	Stage II	T2	Micro	6 - Established Cirrhosis	Hepatitis B	NotAvailable	50.77906557	19.6810922	1.56462182	3.309460907
TCGA-DD-AAVR	MALE	44	Hepatocellular Carcinoma	0	2513	Stage I	T1	None	6 - Established Cirrhosis	Hepatitis B	NotAvailable	7.744355816	10.52354718	4.690963428	1.259310537
TCGA-RC-A7SH	MALE	42	Hepatocellular Carcinoma	0	468	Stage II	T2	Micro	3,4 - Fibrous Speta	Hepatitis B	NotAvailable	192.6492615	45.25693712	4.070914815	3.559663052
TCGA-G3-AAV7	MALE	38	Hepatocellular Carcinoma	0	361	Stage II	T2	Micro	5 - Nodular Formation and Incomplete Cirrhosis	Hepatitis B	Hepatitis B Surface Antiger	106.8044246	44.03867044	4.878304096	5.886164397
TCGA-EP-A12J	MALE	62	Hepatocellular Carcinoma	0	570	Stage I	T1	None	NotAvailable	Hepatitis C	NotAvailable	53.18848029	25.55438145	8.296543295	4.453507434
TCGA-DD-A114	MALE	42	Hepatocellular Carcinoma	1	1149	Stage II	T2	Micro	5 - Nodular Formation and Incomplete Cirrhosis	Hepatitis C	Hepatitis C Virus RNA	102.5492797	16.0029413	2.464405697	3.608413625
TCGA-DD-AADD	MALE	51	Hepatocellular Carcinoma	0	1231	Stage I	T1	None	NotAvailable	Hepatitis B	NotAvailable	275.510312	42.79741474	12.72404637	11.04603212
TCGA-DD-A1EL	MALE	23	Hepatocellular Carcinoma	1	415	Stage II	T2	None	0 - No Fibrosis	Hepatitis B	Hepatitis B Surface Antiger	44.45905908	8.632217834	7.974559241	6.730572247
TCGA-DD-AAE2	MALE	51	Hepatocellular Carcinoma	0	638	Stage I	T1	None	6 - Established Cirrhosis	Hepatitis B	NotAvailable	246.6385523	16.3357645	3.271827228	3.270711011
TCGA-DD-AADC	MALE	53	Hepatocellular Carcinoma	1	425	Stage I	T1	Macro	NotAvailable	Hepatitis B	NotAvailable	111.115912	25.40459636	9.398126706	3.526943737
TCGA-DD-A1EH	MALE	23	Hepatocellular Carcinoma	0	1495	Stage III	T3	Macro	3,4 - Fibrous Speta	Hepatitis B	Hepatitis B Surface Antiger	123.0620211	34.93840124	6.758114407	5.958636403
TCGA-DD-AAD1	FEMALE	51	Hepatocellular Carcinoma	0	564	Stage I	T1	None	NotAvailable	Hepatitis B	NotAvailable	87.95637512	31.86566	2.632586593	5.915725821
TCGA-DD-AAW0	MALE	54	Hepatocellular Carcinoma	0	2015	Stage I	T1	None	3,4 - Fibrous Speta	Hepatitis B	NotAvailable	92.72983606	14.75119788	2.926219257	2.721700008
TCGA-G3-AAV4	FEMALE	83	Hepatocellular Carcinoma	1	27	Stage I	T1	None	3,4 - Fibrous Speta	Hepatitis B	NotAvailable	61.13105925	15.72341021	6.603662432	3.366424235
TCGA-UB-A7MF	MALE	56	Hepatocellular Carcinoma	1	214	Stage IIIA	T3a	Micro	6 - Established Cirrhosis	Hepatitis B	Hepatitis C Antibody	29.98603129	38.27957277	3.134923036	4.071943597
TCGA-DD-AACC	MALE	61	Hepatocellular Carcinoma	1	1685	Stage I	T1	None	6 - Established Cirrhosis	Hepatitis B	NotAvailable	34.70702032	15.0000639	4.658065278	1.744236543
TCGA-DD-AADY	FEMALE	55	Hepatocellular Carcinoma	0	555	Stage I	T1	None	6 - Established Cirrhosis	Hepatitis B	NotAvailable	76.65406344	61.0823058	1.812014788	4.429060475
TCGA-FV-A23B	FEMALE	70	Hepatocellular Carcinoma	1	1852	Stage II	T2	NotAvailable	NotAvailable	Hepatitis C	NotAvailable	50.68394213	20.70666467	10.92522691	5.702100166
TCGA-DD-AADA	FEMALE	66	Hepatocellular Carcinoma	0	1233	Stage I	T1	Macro	NotAvailable	Hepatitis B	NotAvailable	13.52638145	8.319093675	2.939794695	0.902240475
TCGA-DD-AAC9	MALE	51	Hepatocellular Carcinoma	0	347	Stage I	T1	None	6 - Established Cirrhosis	Hepatitis B	NotAvailable	56.56278175	16.3942853	2.371094789	3.152828773
TCGA-DD-AAD0	FEMALE	73	Hepatocellular Carcinoma	0	137	Stage I	T1	None	NotAvailable	Hepatitis B	NotAvailable	135.6281997	23.10309257	4.148754266	3.12617131
TCGA-DD-A4NO	MALE	65	Hepatocellular Carcinoma	0	2245	Stage I	T1	None	0 - No Fibrosis	Hepatitis C	Hepatitis C Virus RNA	105.8240857	8.562818463	2.679466137	4.017252919
TCGA-DD-AAVV	MALE	56	Hepatocellular Carcinoma	0	2455	Stage II	T2	Micro	3,4 - Fibrous Speta	Hepatitis B	NotAvailable	50.7453905	21.01785196	3.344908299	2.589705549
TCGA-DD-A4NQ	MALE	60	Hepatocellular Carcinoma	1	373	Stage II	T2	Macro	NotAvailable	Hepatitis B	Hepatitis C Virus RNA	184.2231051	42.02585505	3.485279411	9.275415967
TCGA-DD-AACE	MALE	62	Hepatocellular Carcinoma	0	2184	Stage I	T1	None	6 - Established Cirrhosis	Hepatitis B	NotAvailable	78.03995486	7.451054094	6.56507473	2.939735613
TCGA-DD-AACD	MALE	48	Hepatocellular Carcinoma	1	381	Stage I	T1	Micro	1,2 - Portal Fibrosis	Hepatitis B	NotAvailable	22.11687848	7.774573354	2.043352897	2.001744438
TCGA-ES-A2HT	MALE	54	Hepatocellular Carcinoma	1	438	Stage I	T1	None	0 - No Fibrosis	Hepatitis C	NotAvailable	29.41584572	29.86534411	0.601138464	1.684021587
TCGA-XR-A8TC	FEMALE	43	Hepatocellular Carcinoma	0	1339	Stage I	T1	None	6 - Established Cirrhosis	Hepatitis B	NotAvailable	65.80291304	9.998084893	2.65018227	3.319410339
TCGA-DD-AAEI	MALE	72	Hepatocellular Carcinoma	0	1531	Stage I	T1	None	6 - Established Cirrhosis	Hepatitis B	NotAvailable	64.36096458	5.46258697	2.727351907	1.829459639
TCGA-DD-AACK	MALE	70	Hepatocellular Carcinoma	0	9	Stage I	T1	Micro	6 - Established Cirrhosis	Hepatitis B	NotAvailable	183.5277152	35.2808815	5.75003522	6.308846841
TCGA-RG-A7D4	MALE	69	Hepatocellular Carcinoma	0	1098	Stage II	T2	NotAvailable	NotAvailable	Hepatitis C	Hepatitis C Antibody	58.4577994	8.222505363	10.89771786	7.710954959
TCGA-RC-A7SK	MALE	59	Hepatocellular Carcinoma	0	472	Stage I	T1	Micro	1,2 - Portal Fibrosis	Hepatitis C	NotAvailable	38.27241917	14.12737358	3.021037412	2.777680829
TCGA-DD-AAVZ	MALE	38	Hepatocellular Carcinoma	0	1900	Stage I	T1	None	6 - Established Cirrhosis	Hepatitis B	NotAvailable	62.93784594	20.849151	7.922898882	3.27377346
TCGA-G3-AAUZ	MALE	48	Hepatocellular Carcinoma	0	480	Stage I	T1	None	1,2 - Portal Fibrosis	Hepatitis B	Hepatitis C Antibody	18.53301925	4.258976939	0.5116102	2.186320322
TCGA-MI-A75C	MALE	64	Hepatocellular Carcinoma	0	291	Stage I	T1	None	NotAvailable	Hepatitis C	HBV Surface Antibody	63.74571257	70.15869963	3.756992099	3.764559076
TCGA-EP-A2KC	MALE	62	Hepatocellular Carcinoma	1	19	Stage I	T1	None	NotAvailable	Hepatitis C	NotAvailable	99.98486277	8.88424776	4.733373142	6.533093477
TCGA-DD-AAD6	MALE	66	Hepatocellular Carcinoma	0	672	Stage IIIA	T3a	None	NotAvailable	Hepatitis B	NotAvailable	84.7011449	25.72715246	14.69678632	8.006447592
TCGA-G3-AAV1	MALE	51	Hepatocellular Carcinoma	1	359	Stage IIIC	T4	Micro	6 - Established Cirrhosis	Hepatitis B	Hepatitis C Antibody	65.1534394	7.372136458	6.004651343	5.584956431
TCGA-G3-A5SI	MALE	44	Hepatocellular Carcinoma	1	768	Stage II	T2	None	6 - Established Cirrhosis	Hepatitis C	NotAvailable	35.23821592	10.51856337	0.449133161	1.210112035
TCGA-DD-AAVU	MALE	46	Hepatocellular Carcinoma	0	2202	Stage II	T2	Micro	3,4 - Fibrous Speta	Hepatitis B	NotAvailable	21.97164127	7.572154625	7.126979588	2.364329774
TCGA-UB-A7ME	MALE	51	Hepatocellular Carcinoma	0	486	Stage I	T1	None	1,2 - Portal Fibrosis	Hepatitis B	Hepatitis B Surface Antiger	35.45277405	17.03431863	5.983884043	2.489490403
TCGA-DD-AACO	MALE	40	Hepatocellular Carcinoma	0	1876	Stage I	T1	None	6 - Established Cirrhosis	Hepatitis B	NotAvailable	7.622869565	11.24756112	0.842706871	1.845958874
TCGA-DD-AADB	MALE	51	Hepatocellular Carcinoma	0	1242	Stage I	T1	None	NotAvailable	Hepatitis B	NotAvailable	215.9735992	40.75177954	13.90062175	5.229352455
TCGA-G3-A3CK	MALE	61	Hepatocellular Carcinoma	0	585	Stage I	T1	None	6 - Established Cirrhosis	Hepatitis B	NotAvailable	55.22751152	6.077709657	2.291661747	2.604285439
TCGA-G3-A25U	FEMALE	63	Hepatocellular Carcinoma	0	1636	Stage I	T1	None	0 - No Fibrosis	Hepatitis B	NotAvailable	192.9447089	8.457721204	1.37537594	3.498760574
TCGA-ED-A7PZ	MALE	61	Hepatocellular Carcinoma	0	6	Stage II	T2	Micro	NotAvailable	No History of Primary Risk Factors	Hepatitis B Surface Antiger	46.22746544	36.98023366	1.355615273	4.014428525
TCGA-DD-A113	FEMALE	55	Hepatocellular Carcinoma	0	2425	Stage II	T2	Micro	0 - No Fibrosis	Other	Hepatitis B Surface Antiger	37.55177296	12.48839578	0.83243879	4.676007212
TCGA-CC-A5UC	MALE	63	Hepatocellular Carcinoma	1	347	Stage IIIA	T3	NotAvailable	NotAvailable	Alcohol consumption	Hepatitis C Antibody	352.3432693	58.6023195	10.3438332	12.03784196
TCGA-DD-A115	MALE	53	Hepatocellular Carcinoma	1	2542	Stage IIIA	T3	None	0 - No Fibrosis	Alcohol consumption	Hepatitis B Surface Antiger	97.60329393	17.36323724	2.243960685	3.83786457
TCGA-CC-A8HV	FEMALE	51	Hepatocellular Carcinoma	1	279	Stage II	T2	NotAvailable	NotAvailable	No History of Primary Risk Factors	Hepatitis C Antibody	155.878269	25.50374357	6.510655293	5.990620782
TCGA-DD-A73E	MALE	66	Hepatocellular Carcinoma	0	44	Stage I	T1	None	0 - No Fibrosis	Alcohol consumption	Hepatitis B Surface Antiger	94.98062956	8.186847636	7.503937599	5.741654166
TCGA-2Y-A9GU	FEMALE	55	Hepatocellular Carcinoma	0	1939	Stage I	T1	NotAvailable	NotAvailable	No History of Primary Risk Factors	Hepatitis C Antibody	123.2390458	41.29981819	2.842026877	2.910287395
TCGA-ED-A4XI	MALE	58	Hepatocellular Carcinoma	0	819	Stage II	T2	Micro	NotAvailable	No History of Primary Risk Factors	Hepatitis B Surface Antiger	45.64009449	38.3624941	3.235690722	2.354498647
TCGA-GJ-A3OU	MALE	59	Hepatocellular Carcinoma	0	879	Stage I	T1	None	NotAvailable	Alcohol consumption	Hepatitis C Antibody	176.0277258	29.05646975	5.749406726	5.049172292
TCGA-CC-A7IJ	MALE	56	Hepatocellular Carcinoma	0	382	Stage II	T2	NotAvailable	NotAvailable	Alcohol consumption	Hepatitis C Antibody	29.40941146	53.18878536	9.95948377	3.85078456
TCGA-CC-A8HU	FEMALE	39	Hepatocellular Carcinoma	1	344	Stage IIIA	T3	NotAvailable	NotAvailable	No History of Primary Risk Factors	Hepatitis C Antibody	31.01665021	5.959179031	9.742199212	2.486943135
TCGA-ZS-A9CF	MALE	64	Hepatocellular Carcinoma	0	2412	Stage II	T2	Micro	0 - No Fibrosis	Hemochromatosis	Hepatitis C Antibody	48.54899912	31.76876975	1.28147202	3.710624129
TCGA-2Y-A9GS	MALE	58	Hepatocellular Carcinoma	1	724	NotAvailable	T2	NotAvailable	NotAvailable	Alcohol consumption	HCV Genotype	98.8013014	21.3948724	5.409158543	4.759835724
TCGA-DD-A39X	FEMALE	78	Hepatocellular Carcinoma	1	1694	Stage I	T1	None	0 - No Fibrosis	No History of Primary Risk Factors	Hepatitis B Surface Antiger	42.31029664	14.22938512	2.459248121	2.455735285
TCGA-ZS-A9CG	MALE	55	Hepatocellular Carcinoma	0	341	Stage II	T2	Micro	0 - No Fibrosis	NotAvailable	Hepatitis C Antibody	36.07056351	4.940350321	2.65109278	1.94975614
TCGA-DD-A118	FEMALE	77	Hepatocellular Carcinoma	0	3437	Stage II	T2	None	0 - No Fibrosis	NotAvailable	Hepatitis B Surface Antiger	37.11812007	18.34720355	3.820890589	2.754213445
TCGA-BD-A2L6	MALE	69	Hepatocellular Carcinoma	0	1363	NotAvailable	T2	Micro	3,4 - Fibrous Speta	Alcohol consumption	Hepatitis B Surface Antiger	50.44725633	18.1104137	4.263692782	1.939972906
TCGA-FV-A3R2	MALE	75	Hepatocellular Carcinoma	1	194	Stage I	T1	NotAvailable	NotAvailable	No History of Primary Risk Factors	Hepatitis C Antibody	60.63167256	13.09210556	7.827583761	6.430484664
TCGA-CC-5261	MALE	44	Hepatocellular Carcinoma	1	97	Stage II	T2	None	NotAvailable	NotAvailable	Hepatitis C Antibody	61.75398231	14.65813544	2.322933777	4.668615557
TCGA-ED-A7PX	FEMALE	48	Hepatocellular Carcinoma	0	6	Stage II	T2	Micro	NotAvailable	No History of Primary Risk Factors	Hepatitis B Surface Antiger	77.28547677	23.27155821	1.170861519	13.37050353
TCGA-DD-A1EB	FEMALE	72	Hepatocellular Carcinoma	0	2017	Stage I	T1	None	0 - No Fibrosis	Other	Hepatitis B Surface Antiger	386.3508109	12.19302708	2.515804759	6.410652568
TCGA-2Y-A9H7	FEMALE	81	Hepatocellular Carcinoma	0	1168	Stage I	T1	None	3,4 - Fibrous Speta	No History of Primary Risk Factors	Hepatitis C Antibody	74.78375467	41.39569846	7.671169932	5.583750946
TCGA-4R-AA8I	MALE	66	Hepatocellular Carcinoma	1	262	Stage II	T2	Micro	6 - Established Cirrhosis	Non-Alcoholic Fatty Liver Disease	Hepatitis C Antibody	4.649227477	1.479036686	2.719484232	1.265169625
TCGA-DD-A11A	MALE	67	Hepatocellular Carcinoma	0	79	Stage I	T1	None	0 - No Fibrosis	Other	Hepatitis B Surface Antiger	202.7973703	22.08159038	11.45240665	9.099840515
TCGA-G3-A7M7	MALE	65	Hepatocellular Carcinoma	0	361	Stage I	T1	None	3,4 - Fibrous Speta	Alcohol consumption	Hepatitis C Antibody	23.62952323	19.86512303	2.349289987	1.983621931
TCGA-DD-A3A7	MALE	67	Hepatocellular Carcinoma	1	419	Stage IIIB	T3b	Micro	0 - No Fibrosis	Alcohol consumption	Hepatitis B Surface Antiger	75.99395026	81.91079604	1.5924461	4.251626928
TCGA-CC-A9FW	MALE	68	Hepatocellular Carcinoma	0	248	Stage IIIA	T3	NotAvailable	NotAvailable	Alcohol consumption	Hepatitis C Antibody	172.8680083	33.56407948	13.13794349	9.910166657
TCGA-5R-AA1D	FEMALE	17	Hepatocellular Carcinoma	0	449	Stage IIIA	T3a	Micro	0 - No Fibrosis	No History of Primary Risk Factors	Hepatitis C Antibody	9.008337356	6.456495899	3.619471077	1.423569143
TCGA-CC-A123	FEMALE	24	Hepatocellular Carcinoma	0	219	Stage IIIA	T3	None	NotAvailable	NotAvailable	Hepatitis C Antibody	41.57970014	8.059349312	11.49412111	4.017051535
TCGA-DD-A4NR	FEMALE	85	Hepatocellular Carcinoma	1	9	Stage I	T1	None	6 - Established Cirrhosis	Other	Hepatitis B Surface Antiger	149.5191275	42.15121392	5.575209299	5.778894284
TCGA-DD-A73B	FEMALE	72	Hepatocellular Carcinoma	1	283	Stage I	T1	None	6 - Established Cirrhosis	Alpha-1 Antitrypsin Deficiency	Hepatitis B Surface Antiger	61.21888336	9.746508163	1.678044937	4.628285428
TCGA-ED-A66Y	FEMALE	51	Hepatocellular Carcinoma	1	296	Stage IIIA	T3a	Micro	NotAvailable	No History of Primary Risk Factors	Hepatitis B Surface Antiger	44.96633728	61.6022001	7.372068179	4.023831366
TCGA-CC-A7II	MALE	54	Hepatocellular Carcinoma	0	399	Stage IIIA	T3	NotAvailable	NotAvailable	Alcohol consumption	Hepatitis C Antibody	32.56525405	6.066664527	5.636599642	6.598449614
TCGA-CC-5263	MALE	35	Hepatocellular Carcinoma	1	129	Stage IIIA	T3	None	NotAvailable	No History of Primary Risk Factors	Hepatitis C Antibody	78.25763913	20.37243958	4.156819839	4.864728773
TCGA-ZS-A9CD	MALE	73	Hepatocellular Carcinoma	1	1386	Stage II	T2	None	5 - Nodular Formation and Incomplete Cirrhosis	NotAvailable	Hepatitis C Antibody	57.68075079	20.24077171	2.620381115	2.046700612
TCGA-K7-A5RG	MALE	66	Hepatocellular Carcinoma	0	519	Stage I	T1	None	NotAvailable	Alcohol consumption	HCV Genotype	27.90201077	12.61613667	8.879424931	2.455176467
TCGA-WX-AA46	MALE	61	Hepatocellular Carcinoma	0	756	Stage II	T2	Macro	0 - No Fibrosis	No History of Primary Risk Factors	Hepatitis C Antibody	44.56836968	8.026018694	2.317561233	1.531956756
TCGA-5R-AA1C	MALE	57	Hepatocellular Carcinoma	0	520	Stage II	T2	Micro	1,2 - Portal Fibrosis	No History of Primary Risk Factors	Hepatitis C Antibody	13.86386392	4.106873862	5.85559917	2.737206407
TCGA-ED-A82E	FEMALE	60	Hepatocellular Carcinoma	0	408	Stage IIIA	T3a	Micro	NotAvailable	No History of Primary Risk Factors	Hepatitis C Antibody	11.07098885	4.943530191	0.579661454	1.481110803
TCGA-UB-A7MC	MALE	59	Hepatocellular Carcinoma	0	500	Stage IIIA	T3a	Micro	1,2 - Portal Fibrosis	Alcohol consumption	Hepatitis C Antibody	35.2313773	3.809392027	3.178604588	4.675241482
TCGA-G3-AAV2	MALE	50	Hepatocellular Carcinoma	0	372	Stage I	T1	None	6 - Established Cirrhosis	Alcohol consumption	Hepatitis C Antibody	60.06901043	16.64311692	3.161845879	2.397366969
TCGA-CC-A3MC	MALE	54	Hepatocellular Carcinoma	0	363	Stage IIIA	T3	NotAvailable	NotAvailable	Alcohol consumption	Hepatitis C Antibody	88.0342438	8.529882528	2.413007846	3.33901839
TCGA-CC-A3MB	MALE	36	Hepatocellular Carcinoma	1	315	Stage IIIA	T3	NotAvailable	NotAvailable	No History of Primary Risk Factors	Hepatitis C Antibody	103.513112	65.70120654	6.308582346	7.871580521
TCGA-ED-A627	MALE	74	Hepatocellular Carcinoma	0	423	Stage I	T1	NotAvailable	NotAvailable	Alcohol consumption	Hepatitis C Antibody	27.78633922	16.46697227	2.436506499	1.543201446
TCGA-DD-A1EK	FEMALE	64	Hepatocellular Carcinoma	1	558	Stage IVB	T4	None	0 - No Fibrosis	Other	Hepatitis B Surface Antiger	101.3639657	19.56154136	1.822307465	4.55990854
TCGA-2Y-A9GZ	FEMALE	82	Hepatocellular Carcinoma	1	848	Stage II	T2	NotAvailable	NotAvailable	No History of Primary Risk Factors	Hepatitis C Antibody	153.7925495	2.586303624	5.29106502	1.653412624
TCGA-2Y-A9HA	MALE	70	Hepatocellular Carcinoma	1	36	Stage II	T2	NotAvailable	3,4 - Fibrous Speta	Alcohol consumption	Hepatitis C Antibody	85.42310782	11.87389198	9.333454665	4.979738932
TCGA-DD-A39Y	MALE	67	Hepatocellular Carcinoma	1	171	Stage I	T1	None	0 - No Fibrosis	No History of Primary Risk Factors	Hepatitis B Surface Antiger	166.4086798	33.68100221	13.24174901	6.59828502
TCGA-UB-AA0U	MALE	60	Hepatocellular Carcinoma	0	327	Stage II	T2	None	0 - No Fibrosis	Non-Alcoholic Fatty Liver Disease	Hepatitis C Antibody	110.6991527	19.68872744	3.228452273	5.064336023
TCGA-BD-A3ER	MALE	62	Hepatocellular Carcinoma	0	1115	Stage II	T2	None	6 - Established Cirrhosis	Hemochromatosis	Hepatitis B Surface Antiger	70.31048954	30.08199124	1.68426832	3.468893691
TCGA-2Y-A9HB	MALE	66	Hepatocellular Carcinoma	0	260	Stage I	T1	None	NotAvailable	Alcohol consumption	Hepatitis C Virus RNA	113.4226544	73.71895475	1.609301666	5.745996483
TCGA-KR-A7K0	MALE	65	Hepatocellular Carcinoma	1	65	Stage I	T1	None	NotAvailable	Alcohol consumption	Hepatitis C Antibody	45.18377581	9.881454938	3.782142061	2.524280926
TCGA-WX-AA47	FEMALE	33	Hepatocellular Carcinoma	1	556	Stage IIIA	T3a	Macro	0 - No Fibrosis	No History of Primary Risk Factors	Hepatitis C Antibody	77.82479241	8.521244602	1.749905076	1.623915939
TCGA-DD-A4NI	MALE	67	Hepatocellular Carcinoma	0	816	Stage II	T2	None	0 - No Fibrosis	Alcohol consumption	Hepatitis B Surface Antiger	54.3276073	10.53683623	1.71136376	1.460707925
TCGA-CC-5260	FEMALE	61	Hepatocellular Carcinoma	1	87	Stage IIIC	T4	None	NotAvailable	NotAvailable	Hepatitis C Antibody	31.59466464	22.69848223	4.545498608	5.288087613
TCGA-FV-A3I1	FEMALE	81	Hepatocellular Carcinoma	1	247	Stage II	T2	Micro	0 - No Fibrosis	Alcohol consumption	Hepatitis C Antibody	239.4858935	36.37915381	6.825140981	5.583106964
TCGA-DD-A73G	FEMALE	73	Hepatocellular Carcinoma	0	3478	Stage I	T1	None	0 - No Fibrosis	No History of Primary Risk Factors	Hepatitis B Surface Antiger	39.65254989	41.22614334	3.323730246	2.370609157
TCGA-UB-AA0V	FEMALE	69	Hepatocellular Carcinoma	0	314	Stage I	NotAvaila	None	0 - No Fibrosis	Hemochromatosis	Hepatitis C Antibody	67.43549711	17.50065179	1.74723534	1.78959338
TCGA-2Y-A9GT	MALE	51	Hepatocellular Carcinoma	1	1624	Stage I	T1	None	NotAvailable	Alcohol consumption	Hepatitis C Virus RNA	67.41940699	32.84095054	3.369772649	3.403193796
TCGA-WJ-A86L	FEMALE	68	Hepatocellular Carcinoma	0	345	Stage I	T1	None	0 - No Fibrosis	No History of Primary Risk Factors	Hepatitis C Antibody	100.0201622	26.37742503	0.303370104	7.636300942
TCGA-CC-A7IE	MALE	57	Hepatocellular Carcinoma	1	217	Stage IIIA	T3	NotAvailable	NotAvailable	Alcohol consumption	Hepatitis C Antibody	129.8837692	80.901433	10.61883058	5.505385886
TCGA-MR-A520	MALE	58	Hepatocellular Carcinoma	0	229	Stage I	T1	NotAvailable	NotAvailable	Other	Hepatitis C Antibody	71.66417691	4.372529035	1.891067018	2.946878084
TCGA-ED-A8O5	FEMALE	59	Hepatocellular Carcinoma	0	406	Stage IIIA	T3a	Micro	NotAvailable	No History of Primary Risk Factors	Hepatitis C Antibody	49.29493513	24.37789971	9.861459031	1.685658837
TCGA-DD-A4NA	FEMALE	67	Hepatocellular Carcinoma	0	1008	Stage IIIC	T2	NotAvailable	5 - Nodular Formation and Incomplete Cirrhosis	No History of Primary Risk Factors	Hepatitis B Surface Antiger	8.255566614	8.931317612	0.559144223	3.478317229
TCGA-DD-A1ED	MALE	68	Hepatocellular Carcinoma	0	2301	Stage I	T1	None	0 - No Fibrosis	Alcohol consumption	Hepatitis B Surface Antiger	10.5533033	11.09449834	3.353769163	1.282369685
TCGA-CC-A9FS	MALE	55	Hepatocellular Carcinoma	0	211	Stage II	T2	NotAvailable	NotAvailable	Alcohol consumption	Hepatitis C Antibody	35.60560074	6.113524488	3.1568057	2.03757406
TCGA-UB-A7MB	MALE	24	Hepatocellular Carcinoma	0	601	Stage II	T2	Micro	0 - No Fibrosis	No History of Primary Risk Factors	Hepatitis C Antibody	333.3578943	18.95175337	4.751915422	11.31364229
TCGA-MI-A75E	MALE	61	Hepatocellular Carcinoma	0	507	Stage IIIC	T4	Micro	1,2 - Portal Fibrosis	Alcohol consumption	HCV Genotype	79.82198185	19.55364563	2.217183565	4.129149759
TCGA-G3-AAV5	MALE	67	Hepatocellular Carcinoma	0	354	Stage II	T2	Macro	6 - Established Cirrhosis	Alcohol consumption	Hepatitis C Antibody	69.65336911	11.20071485	2.010958654	7.471867326
TCGA-2Y-A9H2	FEMALE	64	Hepatocellular Carcinoma	0	1731	Stage I	T1	None	NotAvailable	Alcohol consumption	Hepatitis C Antibody	29.88083286	15.7629821	1.033468664	3.645373348
TCGA-G3-A6UC	MALE	65	Hepatocellular Carcinoma	0	671	Stage IIIB	T3b	Micro	6 - Established Cirrhosis	Alcohol consumption	Hepatitis C Antibody	80.27290176	5.632139759	2.687058356	2.241479214
TCGA-CC-A3MA	MALE	61	Hepatocellular Carcinoma	1	303	Stage IIIA	T3	NotAvailable	NotAvailable	Alcohol consumption	Hepatitis C Antibody	13.65698766	10.67180836	0.682023006	4.805025691
TCGA-2Y-A9H4	MALE	68	Hepatocellular Carcinoma	0	1452	Stage I	T1	None	NotAvailable	Alcohol consumption	Hepatitis C Antibody	8.302740828	2.114172259	1.368243722	1.593188134
TCGA-DD-A4NS	FEMALE	61	Hepatocellular Carcinoma	1	2456	Stage I	T1	None	0 - No Fibrosis	Alcohol consumption	Hepatitis B Surface Antiger	77.08213492	31.99487944	2.832926042	2.500892995
TCGA-CC-A8HS	MALE	18	Hepatocellular Carcinoma	1	300	Stage IIIC	T3	NotAvailable	NotAvailable	No History of Primary Risk Factors	Hepatitis C Antibody	63.29733083	44.74855523	5.26277986	5.812341338
TCGA-DD-A11C	MALE	69	Hepatocellular Carcinoma	0	662	Stage I	T1	None	0 - No Fibrosis	NotAvailable	Hepatitis B Surface Antiger	41.55265831	11.9575257	1.567561013	1.994119502
TCGA-CC-A8HT	MALE	74	Hepatocellular Carcinoma	1	140	Stage IIIA	T3	NotAvailable	NotAvailable	Alcohol consumption	Hepatitis C Antibody	55.21794444	14.76417941	7.13487031	5.190492573
TCGA-CC-A1HT	MALE	50	Hepatocellular Carcinoma	1	101	Stage IIIA	T3	NotAvailable	NotAvailable	No History of Primary Risk Factors	Hepatitis C Antibody	32.62391736	13.72724962	3.576905346	7.416743244
TCGA-ED-A7XP	FEMALE	53	Hepatocellular Carcinoma	0	400	Stage II	T2	Micro	NotAvailable	No History of Primary Risk Factors	Hepatitis B Surface Antiger	35.64054918	10.263986	3.223364835	2.790551453
TCGA-DD-A4NJ	FEMALE	54	Hepatocellular Carcinoma	0	928	Stage II	T2	None	1,2 - Portal Fibrosis	Non-Alcoholic Fatty Liver Disease	Hepatitis B Surface Antiger	107.3294151	8.662809341	1.819598365	8.15088811
TCGA-ED-A459	MALE	47	Hepatocellular Carcinoma	0	910	Stage II	T2	Micro	NotAvailable	NotAvailable	Hepatitis B Surface Antiger	63.32819473	42.47811955	13.76195716	4.795209646
TCGA-ZS-A9CE	FEMALE	79	Hepatocellular Carcinoma	0	1241	Stage II	T2	Micro	0 - No Fibrosis	NotAvailable	Hepatitis C Antibody	134.6956032	7.978296035	0.320795758	1.959726155
TCGA-CC-A5UE	MALE	48	Hepatocellular Carcinoma	1	272	Stage IIIB	T4	NotAvailable	NotAvailable	Alcohol consumption	Hepatitis C Antibody	122.4294361	40.69655053	14.91296259	5.094792483
TCGA-2Y-A9H1	MALE	58	Hepatocellular Carcinoma	1	1229	Stage I	T1	NotAvailable	1,2 - Portal Fibrosis	Alcohol consumption	Hepatitis C Antibody	39.62069251	30.46595112	1.535581289	1.937251956
TCGA-2Y-A9GV	FEMALE	54	Hepatocellular Carcinoma	1	2532	Stage I	T1	NotAvailable	NotAvailable	Alcohol consumption	Hepatitis C Antibody	173.1542765	13.93745299	2.461680226	2.339694565
TCGA-CC-5264	MALE	71	Hepatocellular Carcinoma	1	102	Stage IIIA	T3	None	NotAvailable	NotAvailable	Hepatitis C Antibody	63.66102835	32.90913141	5.974803858	6.694134318
TCGA-CC-A7IH	MALE	58	Hepatocellular Carcinoma	0	365	Stage IIIA	T3	NotAvailable	NotAvailable	Alcohol consumption	Hepatitis C Antibody	52.35567593	9.454729837	3.506751884	2.630128985
TCGA-CC-5262	MALE	67	Hepatocellular Carcinoma	1	103	Stage IIIC	T4	NotAvailable	NotAvailable	No History of Primary Risk Factors	Hepatitis C Antibody	164.6504411	33.37674152	5.158298356	4.132235679
TCGA-ED-A5KG	FEMALE	60	Hepatocellular Carcinoma	0	854	Stage II	T2	Micro	NotAvailable	No History of Primary Risk Factors	Hepatitis B Surface Antiger	51.8344541	18.57790416	5.128315702	3.42355759
TCGA-DD-A4NV	MALE	61	Hepatocellular Carcinoma	0	2398	Stage IIIA	T3	None	0 - No Fibrosis	Non-Alcoholic Fatty Liver Disease	Hepatitis B Surface Antiger	454.0000166	15.91643957	3.654609681	3.48491532
TCGA-DD-A4NL	MALE	46	Hepatocellular Carcinoma	0	1711	Stage I	T1	None	0 - No Fibrosis	Alcohol consumption	Hepatitis B Surface Antiger	47.1195854	27.875703	3.589342469	2.125143177

TCGA-DD-A73A	MALE	71	Hepatocellular Carcinoma	0	728	Stage I	T1	None	3,4 - Fibrous Speta	Alcohol consumption	Hepatitis B Surface Antigen	58.36501265	31.17096798	4.168904839	3.579579729
TCGA-CC-A7IF	MALE	59	Hepatocellular Carcinoma	1	649	Stage IIIA	T3	NotAvailable	NotAvailable	Alcohol consumption	Hepatitis C Antibody	68.41921755	5.013834472	1.80324018	3.570664224
TCGA-G3-A7M9	MALE	70	Hepatocellular Carcinoma	1	56	Stage IIIB	T3b	Macro	6 - Established Cirrhosis	Hemochromatosis	Hepatitis C Antibody	57.0626093	12.76573097	12.60379679	5.920305874
TCGA-DD-A1EF	FEMALE	57	Hepatocellular Carcinoma	1	394	Stage I	T1	None	3,4 - Fibrous Speta	No History of Primary Risk Factors	Hepatitis B Surface Antigen	29.33255981	12.56949509	23.12914344	3.723312112
TCGA-DD-A4NG	MALE	77	Hepatocellular Carcinoma	1	802	Stage IIIA	T3a	Micro	NotAvailable	Alcohol consumption	Hepatitis B Surface Antigen	105.1912799	27.36019748	7.312236641	4.835965457
TCGA-CC-A3M9	MALE	45	Hepatocellular Carcinoma	1	300	Stage IIIA	T3	NotAvailable	NotAvailable	Alcohol consumption	Hepatitis C Antibody	12.51887873	18.24087687	9.548144473	2.382155337
TCGA-MI-A75G	MALE	63	Hepatocellular Carcinoma	0	698	Stage II	T2	None	6 - Established Cirrhosis	Alcohol consumption	HCV Genotype	317.3240035	175.9084908	5.206287872	2.644820116
TCGA-ED-A7XO	MALE	29	Hepatocellular Carcinoma	0	427	Stage IIIA	T3a	Micro	NotAvailable	No History of Primary Risk Factors	Hepatitis C Antibody	29.86465449	6.937619317	1.862672971	1.395479541
TCGA-ED-A66X	MALE	35	Hepatocellular Carcinoma	0	406	Stage IIIA	T3a	Micro	NotAvailable	Alcohol consumption	Hepatitis B Surface Antigen	179.6117372	23.81663371	6.613418521	4.41439382
TCGA-5R-AAAM	FEMALE	65	Hepatocellular Carcinoma	1	46	Stage II	T2	Micro	6 - Established Cirrhosis	No History of Primary Risk Factors	Hepatitis C Antibody	40.78570908	11.09734557	3.397863446	2.87683988
TCGA-2Y-A9H5	FEMALE	59	Hepatocellular Carcinoma	1	555	Stage I	T1	None	1,2 - Portal Fibrosis	Alcohol consumption	Hepatitis C Antibody	82.77895557	27.63646264	3.750127808	3.369707914
TCGA-DD-A4NP	MALE	32	Hepatocellular Carcinoma	0	3308	Stage I	T1	None	0 - No Fibrosis	No History of Primary Risk Factors	Hepatitis B Surface Antigen	47.08663945	13.8758756	2.932378518	2.103589802
TCGA-CC-A7IK	MALE	59	Hepatocellular Carcinoma	1	262	Stage IIIA	T3	NotAvailable	NotAvailable	Alcohol consumption	Hepatitis C Antibody	123.7561075	8.521139091	10.81419273	8.58132072
TCGA-G3-A7M5	MALE	76	Hepatocellular Carcinoma	0	447	Stage I	T1	None	0 - No Fibrosis	Alcohol consumption	Hepatitis C Antibody	136.4911631	3.258287589	1.555516943	1.287162383
TCGA-CC-A9FV	MALE	57	Hepatocellular Carcinoma	0	0	Stage IIIA	T3	NotAvailable	NotAvailable	Alcohol consumption	Hepatitis C Antibody	7.271537814	9.373383224	22.6032698	1.806409358
TCGA-DD-A3A8	MALE	75	Hepatocellular Carcinoma	1	11	Stage II	T2	None	0 - No Fibrosis	No History of Primary Risk Factors	Hepatitis B Surface Antigen	59.41352446	12.69470208	2.54449943	2.511960073
TCGA-G3-AAV6	FEMALE	53	Hepatocellular Carcinoma	1	65	Stage IIIA	T3a	Micro	0 - No Fibrosis	No History of Primary Risk Factors	Hepatitis B Surface Antigen	77.91199587	25.60160645	8.185790793	2.922284136
TCGA-2Y-A9H3	MALE	45	Hepatocellular Carcinoma	0	1516	Stage II	T2	Micro	1,2 - Portal Fibrosis	Alcohol consumption	Hepatitis C Antibody	9.9427272	12.39870232	2.868148414	1.001086034
TCGA-CC-A7IG	MALE	47	Hepatocellular Carcinoma	1	299	Stage II	T2	NotAvailable	NotAvailable	Alcohol consumption	Hepatitis C Antibody	179.7765947	75.57058812	11.98105806	4.366017857
TCGA-DD-A1EG	MALE	76	Hepatocellular Carcinoma	1	1372	Stage I	T1	None	1,2 - Portal Fibrosis	Alcohol consumption	Hepatitis B Surface Antigen	52.36435474	17.43668564	4.979361875	3.341142142
TCGA-DD-A73C	FEMALE	65	Hepatocellular Carcinoma	0	701	Stage IIIA	T3a	None	0 - No Fibrosis	No History of Primary Risk Factors	Hepatitis B Surface Antigen	237.5359837	7.586085991	3.787100048	5.874619246
TCGA-G3-A7M8	MALE	31	Hepatocellular Carcinoma	0	430	Stage I	T1	None	6 - Established Cirrhosis	Alcohol consumption	Hepatitis C Antibody	8.215694013	4.199130344	0.969059785	1.651170104
TCGA-CC-A7IL	MALE	61	Hepatocellular Carcinoma	1	278	Stage IIIA	T3	NotAvailable	NotAvailable	Alcohol consumption	Hepatitis C Antibody	226.4494305	17.50475891	2.623365917	11.45662986
TCGA-DD-A4NH	FEMALE	65	Hepatocellular Carcinoma	0	917	Stage IIIB	T3b	Macro	1,2 - Portal Fibrosis	Alcohol consumption	Hepatitis B Surface Antigen	67.57764516	18.73056935	5.942659327	3.879539714
TCGA-WX-AA44	FEMALE	64	Hepatocellular Carcinoma	0	615	Stage I	T1	None	6 - Established Cirrhosis	Non-Alcoholic Fatty Liver Disease	Hepatitis C Antibody	35.74759224	16.49625628	2.186843892	3.68234806
TCGA-UB-A7MD	MALE	67	Hepatocellular Carcinoma	1	52	Stage I	T1	Micro	6 - Established Cirrhosis	Alcohol consumption	Hepatitis C Antibody	61.6625228	7.390882671	5.922962419	2.488767735
TCGA-CC-A5UD	MALE	45	Hepatocellular Carcinoma	1	304	Stage IIIA	T3	NotAvailable	NotAvailable	Alcohol consumption	Hepatitis C Antibody	54.35557581	63.10025463	12.04761673	7.07800757
TCGA-ED-A7PY	FEMALE	20	Hepatocellular Carcinoma	0	390	Stage II	T2	Micro	NotAvailable	No History of Primary Risk Factors	Hepatitis B Surface Antigen	22.87971457	25.0985338	4.231924005	1.806584609
TCGA-DD-A4NK	FEMALE	80	Hepatocellular Carcinoma	1	1210	Stage IIIA	T3	None	0 - No Fibrosis	No History of Primary Risk Factors	Hepatitis B Surface Antigen	143.4113918	6.356812583	1.033035009	2.438914152
TCGA-DD-A1EJ	FEMALE	71	Hepatocellular Carcinoma	1	1005	Stage IIIC	T1	None	0 - No Fibrosis	No History of Primary Risk Factors	Hepatitis B Surface Antigen	196.6064199	65.64743725	19.57166118	10.64990904
TCGA-CC-5258	MALE	48	Hepatocellular Carcinoma	1	129	Stage II	T2	None	NotAvailable	NotAvailable	Hepatitis C Antibody	138.1099645	20.85487555	14.05592295	7.458566297
TCGA-DD-A3A6	FEMALE	72	Hepatocellular Carcinoma	1	3258	Stage II	T2	Micro	0 - No Fibrosis	No History of Primary Risk Factors	Hepatitis B Surface Antigen	5.82791927	5.014243722	64.39303131	0.940745129
TCGA-2Y-A9GX	MALE	68	Hepatocellular Carcinoma	0	2442	Stage I	T1	NotAvailable	NotAvailable	Alcohol consumption	Hepatitis B Surface Antigen	77.99422282	24.44129941	2.41069751	2.96988247
TCGA-DD-A11B	MALE	73	Hepatocellular Carcinoma	1	14	Stage I	T1	None	6 - Established Cirrhosis	Non-Alcoholic Fatty Liver Disease	Hepatitis B Surface Antigen	27.38670205	3.424652504	4.745184239	1.502475647
TCGA-DD-A4NE	FEMALE	75	Hepatocellular Carcinoma	1	660	Stage IIIA	T3a	None	0 - No Fibrosis	No History of Primary Risk Factors	Hepatitis C Virus RNA	137.018847	12.6953084	1.051236579	4.482108729
TCGA-K7-A6G5	MALE	66	Hepatocellular Carcinoma	0	512	Stage I	T1	None	NotAvailable	Alcohol consumption	HCV Genotype	238.5316393	58.16386567	1.88907723	2.179768743
TCGA-CC-5259	FEMALE	60	Hepatocellular Carcinoma	0	250	Stage IIIC	T4	NotAvailable	NotAvailable	No History of Primary Risk Factors	Hepatitis C Antibody	16.35628616	25.18456077	8.897492042	1.309887486
TCGA-2Y-A9H8	FEMALE	85	Hepatocellular Carcinoma	1	633	NotAvailable	T1	NotAvailable	NotAvailable	Alcohol consumption	Hepatitis C Virus RNA	130.8545634	23.56848438	4.548001136	2.189085139
TCGA-HP-A5MZ	MALE	78	Hepatocellular Carcinoma	1	91	Stage I	T1	None	NotAvailable	No History of Primary Risk Factors	Hepatitis C Antibody	66.34265366	20.30384517	2.421898438	2.684095033
TCGA-ED-A8O6	FEMALE	50	Hepatocellular Carcinoma	1	56	Stage IIIA	T3a	Micro	NotAvailable	No History of Primary Risk Factors	Hepatitis C Antibody	125.0288171	36.57743402	9.730002951	7.06355762

3. non-viral

barcode	gender	Age at diagnosis	histological type	FU OS status	FU days	pathologic stage	tnm.t	vascular tumor cell type	fibrosis ishak score	hist hepato carc fact	viral hepatitis serology	FAS	SREBF1	PPARG	ACACA
TCGA-DD-AACX	MALE	66	Hepatocellular Carcinoma	0	170	Stage II	T2	None	3,4 - Fibrous Speta	Alcohol consumption	NotAvailable	110.3545815	40.22657352	0.828350478	6.308463591
TCGA-BC-A10U	MALE	69	Hepatocellular Carcinoma	1	837	NotAvailable	T2	NotAvailable	NotAvailable	Alcohol consumption	NotAvailable	68.25064326	44.57964375	3.248799811	4.507946886
TCGA-DD-A39W	FEMALE	29	Hepatocellular Carcinoma	1	827	Stage III	T3	None	NotAvailable	No History of Primary Risk Factors	NotAvailable	146.8902539	16.92549598	7.051987713	7.761207652
TCGA-EP-A26S	MALE	70	Hepatocellular Carcinoma	0	608	Stage I	T1	None	0 - No Fibrosis	Alcohol consumption	NotAvailable	95.07114356	41.68039782	3.200925301	5.208870163
TCGA-G3-A3CI	MALE	71	Hepatocellular Carcinoma	0	180	Stage I	T1	None	0 - No Fibrosis	Non-Alcoholic Fatty Liver Disease	NotAvailable	25.24240905	3.141053279	3.145160138	1.771717721
TCGA-ZP-A9D0	FEMALE	67	Hepatocellular Carcinoma	0	1091	NotAvailable	T1	None	0 - No Fibrosis	Non-Alcoholic Fatty Liver Disease	NotAvailable	14.12772427	6.776177729	2.522731601	0.976602205
TCGA-DD-AADU	MALE	60	Hepatocellular Carcinoma	0	554	Stage II	T2	Micro	3,4 - Fibrous Speta	Alcohol consumption	NotAvailable	18.74922347	7.354447331	4.061550401	1.138001701
TCGA-DD-AAEA	MALE	65	Hepatocellular Carcinoma	0	575	Stage I	T1	None	1,2 - Portal Fibrosis	Alcohol consumption	NotAvailable	96.18540521	8.110674102	3.998302508	3.310961535
TCGA-DD-A3A4	MALE	37	Hepatocellular Carcinoma	1	612	Stage IIIA	T3	None	0 - No Fibrosis	Other	NotAvailable	63.13735095	25.23407299	3.925411971	3.010452779
TCGA-BC-A10X	FEMALE	52	Hepatocellular Carcinoma	1	770	Stage IIIA	T3a	Micro	NotAvailable	No History of Primary Risk Factors	NotAvailable	42.2693865	24.72597022	2.330830076	1.669190239
TCGA-DD-AADS	MALE	63	Hepatocellular Carcinoma	0	474	Stage I	T1	None	NotAvailable	Alcohol consumption	NotAvailable	4.246434955	3.946627623	1.732744477	0.837478902
TCGA-BC-A216	FEMALE	62	Hepatocellular Carcinoma	0	1351	Stage IIIA	T3	None	0 - No Fibrosis	No History of Primary Risk Factors	NotAvailable	74.63144503	88.87105276	4.719073507	4.193764586
TCGA-BC-A3KF	FEMALE	66	Hepatocellular Carcinoma	0	8	Stage I	T1	None	NotAvailable	No History of Primary Risk Factors	NotAvailable	81.78618545	23.24642317	4.344645087	3.871896326
TCGA-EP-A3JL	MALE	76	Hepatocellular Carcinoma	0	303	Stage I	T1	None	NotAvailable	Alcohol consumption	NotAvailable	40.91387732	15.25735805	3.093293347	1.91012291
TCGA-5C-A9VH	MALE	70	Hepatocellular Carcinoma	0	322	Stage I	T1	None	NotAvailable	No History of Primary Risk Factors	NotAvailable	33.94873621	19.08315547	2.872009373	2.880274837
TCGA-5C-A9VG	MALE	58	Hepatocellular Carcinoma	0	328	Stage II	T2	Micro	NotAvailable	Other	NotAvailable	103.4613073	25.86997558	3.757217363	10.20932435
TCGA-NI-A8LF	MALE	74	Hepatocellular Carcinoma	0	799	Stage I	T1	None	NotAvailable	Alcohol consumption	NotAvailable	150.9229965	6.634075063	7.67551391	3.906992301
TCGA-DD-A11D	FEMALE	57	Hepatocellular Carcinoma	1	1560	Stage I	T1	None	6 - Established Cirrhosis	Non-Alcoholic Fatty Liver Disease	NotAvailable	130.0341656	21.58690057	2.859404368	2.742900346
TCGA-BC-A10Q	FEMALE	72	Hepatocellular Carcinoma	1	1135	NotAvailable	T2	None	NotAvailable	No History of Primary Risk Factors	NotAvailable	16.42605231	15.2541232	4.150982032	1.79817636
TCGA-DD-AAEH	MALE	73	Hepatocellular Carcinoma	0	784	Stage I	T1	Micro	1,2 - Portal Fibrosis	NotAvailable	NotAvailable	6.579694738	6.252246761	3.02425637	0.700111601
TCGA-ZP-A9D1	FEMALE	56	Hepatocellular Carcinoma	0	21	NotAvailable	T1	None	5 - Nodular Formation and Incomplete Cirrhos	Non-Alcoholic Fatty Liver Disease	NotAvailable	33.7998475	13.27867022	4.307229109	2.249402704
TCGA-KR-A7K7	FEMALE	61	Hepatocellular Carcinoma	0	951	Stage II	T2	Micro	NotAvailable	No History of Primary Risk Factors	NotAvailable	22.55157517	5.1310501	5.20713003	2.407748896
TCGA-DD-AADG	MALE	70	Hepatocellular Carcinoma	0	1145	Stage IIIA	T3a	None	3,4 - Fibrous Speta	Alcohol consumption	NotAvailable	320.9120891	16.91143286	1.5886101	8.517032559
TCGA-BC-A5W4	MALE	69	Hepatocellular Carcinoma	1	547	Stage IIIA	T3a	Micro	NotAvailable	Alcohol consumption	NotAvailable	24.46506493	7.392713554	4.002043076	2.183088487
TCGA-DD-AAW2	MALE	69	Hepatocellular Carcinoma	0	1855	Stage I	T1	None	6 - Established Cirrhosis	Non-Alcoholic Fatty Liver Disease	NotAvailable	42.32141451	47.15192199	3.38816584	2.684991279
TCGA-BD-A3EP	FEMALE	75	Hepatocellular Carcinoma	0	409	Stage I	T1	None	1,2 - Portal Fibrosis	No History of Primary Risk Factors	NotAvailable	126.5758919	13.19447854	7.077346344	2.779479635
TCGA-DD-AAEG	FEMALE	59	Hepatocellular Carcinoma	0	719	Stage I	T1	None	6 - Established Cirrhosis	No History of Primary Risk Factors	NotAvailable	52.80917949	10.5030274	1.882628961	1.909960486
TCGA-BC-A110	FEMALE	51	Hepatocellular Carcinoma	1	2116	NotAvailable	T1	None	NotAvailable	Alcohol consumption	NotAvailable	14.2600088	7.724585331	3.035238171	0.728939998
TCGA-DD-AADV	MALE	50	Hepatocellular Carcinoma	0	574	Stage I	T1	None	6 - Established Cirrhosis	Alcohol consumption	NotAvailable	32.85571063	9.676485955	6.602604716	1.315358049
TCGA-EP-A2KA	FEMALE	52	Hepatocellular Carcinoma	1	627	Stage IIIA	T3a	NotAvailable	1,2 - Portal Fibrosis	No History of Primary Risk Factors	NotAvailable	30.09406873	57.00053874	7.458825337	3.081580811
TCGA-DD-AAD5	MALE	54	Hepatocellular Carcinoma	0	1345	Stage I	T1	None	3,4 - Fibrous Speta	Alcohol consumption	NotAvailable	65.68215061	56.9462891	8.188373036	7.886950092
TCGA-ZP-A9CV	MALE	59	Hepatocellular Carcinoma	1	1088	NotAvailable	T1	None	NotAvailable	Alcohol consumption	NotAvailable	96.25878612	60.25695281	2.349004373	3.45344669
TCGA-KR-A7K8	MALE	57	Hepatocellular Carcinoma	0	906	Stage I	T1	None	NotAvailable	No History of Primary Risk Factors	NotAvailable	90.26559562	22.11520485	7.671383204	1.107703422
TCGA-HP-A5N0	FEMALE	90	Hepatocellular Carcinoma	1	1147	NotAvailable	TX	NotAvailable	0 - No Fibrosis	No History of Primary Risk Factors	NotAvailable	39.84123449	7.915800113	2.759848932	1.640960717
TCGA-NI-A4U2	MALE	71	Hepatocellular Carcinoma	1	1791	Stage IIIA	T3	None	1,2 - Portal Fibrosis	Alcohol consumption	NotAvailable	55.27210876	6.184697907	4.59662587	1.643960598
TCGA-G3-A5SL	MALE	70	Hepatocellular Carcinoma	0	621	Stage II	T2	Micro	0 - No Fibrosis	Alcohol consumption	NotAvailable	159.8903676	6.353057295	3.200623583	10.28536525
TCGA-DD-A3A3	MALE	45	Hepatocellular Carcinoma	1	535	Stage I	T1	Macro	0 - No Fibrosis	No History of Primary Risk Factors	NotAvailable	67.61481263	10.99815801	2.574215355	3.532728402
TCGA-DD-AAVY	MALE	56	Hepatocellular Carcinoma	0	1970	Stage IIIA	T3	Micro	0 - No Fibrosis	No History of Primary Risk Factors	NotAvailable	50.43033876	13.73000775	2.67330824	3.929209134
TCGA-BW-A5NQ	MALE	63	Hepatocellular Carcinoma	0	0	Stage I	T1	None	6 - Established Cirrhosis	Alcohol consumption	NotAvailable	71.40372799	7.949008686	11.77911903	7.078588924
TCGA-DD-A1EC	FEMALE	20	Hepatocellular Carcinoma	0	602	Stage I	T1	None	0 - No Fibrosis	NotAvailable	NotAvailable	28.54724958	31.79494068	2.446980626	6.124338501
TCGA-FV-A3R3	FEMALE	38	Hepatocellular Carcinoma	1	366	Stage I	T1	None	NotAvailable	No History of Primary Risk Factors	NotAvailable	12.41658589	24.99075418	4.492028329	2.553023486
TCGA-O8-A75V	MALE	54	Hepatocellular Carcinoma	0	538	Stage I	T1	None	6 - Established Cirrhosis	Alcohol consumption	NotAvailable	55.54877714	22.99367399	3.913312835	3.430389811
TCGA-BC-A69H	MALE	64	Hepatocellular Carcinoma	0	444	Stage II	T2	Micro	NotAvailable	Alcohol consumption	NotAvailable	197.5220198	32.85330165	18.4217719	8.272651453
TCGA-DD-AAED	MALE	51	Hepatocellular Carcinoma	0	763	Stage I	T1	None	1,2 - Portal Fibrosis	Alcohol consumption	NotAvailable	21.12528064	33.71987663	7.589523711	4.099811253
TCGA-RC-A6M4	FEMALE	74	Hepatocellular Carcinoma	0	22	Stage IIIA	T3	None	NotAvailable	No History of Primary Risk Factors	NotAvailable	131.530462	30.06255001	2.732691975	7.381488159
TCGA-DD-AACP	MALE	64	Hepatocellular Carcinoma	0	415	Stage I	T1	None	NotAvailable	Alcohol consumption	NotAvailable	117.2442141	19.75110673	12.48199463	6.509160985
TCGA-FV-A4ZP	MALE	78	Hepatocellular Carcinoma	1	2486	Stage IIIA	T3	Micro	NotAvailable	Alcohol consumption	NotAvailable	57.97509595	26.25301525	2.279576204	2.260646115
TCGA-LG-A9QC	MALE	48	Hepatocellular Carcinoma	0	425	Stage I	T1	Micro	NotAvailable	No History of Primary Risk Factors	NotAvailable	324.1306685	43.01674817	2.834358622	4.627048985
TCGA-BC-A217	FEMALE	75	Hepatocellular Carcinoma	1	1397	Stage II	T2	Micro	0 - No Fibrosis	Alcohol consumption	NotAvailable	26.68174018	14.95798181	5.264557559	1.920575177
TCGA-DD-AADL	MALE	58	Hepatocellular Carcinoma	0	636	Stage I	T1	Micro	NotAvailable	Alcohol consumption	NotAvailable	65.1943466	15.25482267	4.070707072	5.101438915
TCGA-2Y-A9H9	MALE	70	Hepatocellular Carcinoma	0	697	Stage I	T1	NotAvailable	NotAvailable	Alcohol consumption	NotAvailable	74.71218183	5.73465591	2.113919225	2.595891863
TCGA-G3-A5SJ	MALE	59	Hepatocellular Carcinoma	0	698	Stage I	T1	None	6 - Established Cirrhosis	Alcohol consumption	NotAvailable	13.38628109	41.74749306	3.420392505	2.514032376
TCGA-G3-AAV3	FEMALE	58	Hepatocellular Carcinoma	0	412	Stage II	T2	None	6 - Established Cirrhosis	Alcohol consumption	NotAvailable	39.76988148	8.760149078	3.81974026	2.376179135
TCGA-BC-A10Z	FEMALE	62	Hepatocellular Carcinoma	1	34	Stage I	T1	None	NotAvailable	No History of Primary Risk Factors	NotAvailable	534.7769863	31.48728426	10.59181365	18.56039969
TCGA-BC-A10T	MALE	76	Hepatocellular Carcinoma	1	837	NotAvailable	T4	None	1,2 - Portal Fibrosis	No History of Primary Risk Factors	NotAvailable	94.96374176	11.75179443	1.073698718	4.769200853
TCGA-G3-A25V	MALE	68	Hepatocellular Carcinoma	0	860	Stage I	T1	None	6 - Established Cirrhosis	Alcohol consumption	NotAvailable	41.26864913	23.72280415	2.985473228	2.132132203
TCGA-DD-AACW	MALE	43	Hepatocellular Carcinoma	0	1424	Stage I	T1	None	NotAvailable	Alcohol consumption	NotAvailable	43.43051679	44.27545585	12.50710244	4.596389763
TCGA-T1-A6J8	MALE	68	Hepatocellular Carcinoma	0	23	NotAvailable	T1	None	NotAvailable	Alcohol consumption	NotAvailable	31.9874301	9.297626914	6.837452883	2.410825597
TCGA-DD-A3A5	FEMALE	66	Hepatocellular Carcinoma	1	3125	Stage III	T3	None	0 - No Fibrosis	No History of Primary Risk Factors	NotAvailable	39.6665923	14.1544423	0.703398688	0.671737332
TCGA-DD-AACV	MALE	53	Hepatocellular Carcinoma	0	1531	Stage I	T1	Micro	NotAvailable	Alcohol consumption	NotAvailable	84.27829016	19.31048376	1.102194105	2.486544788
TCGA-2Y-A9GW	MALE	64	Hepatocellular Carcinoma	1	1271	Stage I	T1	NotAvailable	NotAvailable	Alcohol consumption	NotAvailable	34.64177538	14.51388412	2.425484574	2.271759246
TCGA-PD-A5DF	FEMALE	58	Hepatocellular Carcinoma	1	639	Stage IIIB	T4	NotAvailable	NotAvailable	No History of Primary Risk Factors	NotAvailable	51.58443238	11.49532168	4.806020378	4.50532432
TCGA-BC-A69I	MALE	69	Hepatocellular Carcinoma	0	387	Stage I	T1	None	NotAvailable	No History of Primary Risk Factors	NotAvailable	58.03627745	6.86136934	2.527184284	1.545488215
TCGA-ZP-A9D4	FEMALE	64	Hepatocellular Carcinoma	0	395	NotAvailable	T1	None	0 - No Fibrosis	No History of Primary Risk Factors	NotAvailable	85.6673349	23.99595693	2.660595792	6.833362029
TCGA-FV-A2OR	MALE	75	Hepatocellular Carcinoma	1	581	Stage I	T1	NotAvailable	NotAvailable	Other	NotAvailable	179.8452452	29.92853388	0.545774422	4.324096361
TCGA-DD-AAE7	MALE	72	Hepatocellular Carcinoma	0	644	Stage I	T1	None	3,4 - Fibrous Speta	Alcohol consumption	NotAvailable	10.90129429	7.248101415	2.308531417	1.037988971
TCGA-DD-AAW3	MALE	69	Hepatocellular Carcinoma	0	1633	Stage I	T1	None	0 - No Fibrosis	No History of Primary Risk Factors	NotAvailable	69.76200786	29.10505955	3.736415175	4.107677555
TCGA-WQ-A9G7	FEMALE	71	Hepatocellular Carcinoma	0	30	NotAvailable	T3a	None	NotAvailable	No History of Primary Risk Factors	NotAvailable	87.92366374	62.82614167	4.165640719	8.978180208
TCGA-DD-A4ND	FEMALE	56	Hepatocellular Carcinoma	0	2746	Stage I	T1	None	0 - No Fibrosis	No History of Primary Risk Factors	NotAvailable	160.4854831	44.79147192	4.780462753	5.272453702
TCGA-DD-A3A9	FEMALE	64	Hepatocellular Carcinoma	1	931	Stage IVB	T4	None	0 - No Fibrosis	Other	NotAvailable	30.1890054	30.91599893	2.83514089	3.159928132
TCGA-FV-A2OO	MALE	80	Hepatocellular Carcinoma	0	729	Stage I	T1	None	NotAvailable	Alcohol consumption	NotAvailable	38.56819904	15.48962751	3.309940275	2.2980919
TCGA-ZP-A9CY	FEMALE	66	Hepatocellular Carcinoma	0	782	NotAvailable	T1	None	6 - Established Cirrhosis	Non-Alcoholic Fatty Liver Disease	NotAvailable	54.45559309	25.92447548	2.176435128	2.661806762
TCGA-DD-AADJ	FEMALE	70	Hepatocellular Carcinoma	0	1066	Stage I	T1	None	NotAvailable	NotAvailable	NotAvailable	55.29889873	57.41362016	3.464909143	3.217550195
TCGA-GJ-A9DB	MALE	68	Hepatocellular Carcinoma	1	67	Stage I	T1	None	NotAvailable	No History of Primary Risk Factors	NotAvailable	80.21766099	20.71122322	2.812368687	4.485808446
TCGA-2V-A95S	MALE	NotAvailable	Hepatocellular Carcinoma	0	NotAvaila	Stage II	T2	Micro	6 - Established Cirrhosis	Other	NotAvailable	60.2237991	46.09881458	8.500983844	3.658097649
TCGA-DD-AACZ	FEMALE	63	Hepatocellular Carcinoma	1	171	Stage I	T1	Macro	NotAvailable	NotAvailable	NotAvailable	293.6570647	68.82404257	21.43795095	12.48052214
TCGA-LG-A9OD	MALE	68	Hepatocellular Carcinoma	0	366	Stage IIIA	T3a	None	NotAvailable	No History of Primary Risk Factors	NotAvailable	39.85410446	21.49984847	3.244306786	1.734402841
TCGA-DD-AADN	MALE	59	Hepatocellular Carcinoma	0	898	Stage I	T1	Micro	NotAvailable	Alcohol consumption	NotAvailable	140.9267061	28.38084853	11.26167576	3.299471576
TCGA-BC-4072	FEMALE	74	Hepatocellular Carcinoma	1	1490	Stage IIIA	T3	Macro	NotAvailable	No History of Primary Risk Factors	NotAvailable	26.19817708	18.12193184	3.584001556	5.3826676
TCGA-DD-AACJ	MALE	75	Hepatocellular Carcinoma	0	2102	Stage II	T2	Micro	NotAvailable	NotAvailable	NotAvailable	89.46705523	11.05756037	3.570381319	8.113809877
TCGA-ES-A2HS	MALE	80	Hepatocellular Carcinoma	1	688	Stage I	T1	None	0 - No Fibrosis	No History of Primary Risk Factors	NotAvailable	10.86727474	37.82148528	4.184989341	1.914879785
TCGA-RC-A6M3	MALE	24	Hepatocellular Carcinoma	0	0	Stage II	T2	Micro	NotAvailable	NotAvailable	NotAvailable	23.28338654	24.05675741	2.10800764	4.35510046
TCGA-2Y-A9H0	MALE	49	Hepatocellular Carcinoma	0	3675	Stage IIIA	T3	NotAvailable	NotAvailable	Alcohol consumption	NotAvailable	83.04038468	20.61132463	2.720367709	3.392066983
TCGA-DD-A39V	MALE	77	Hepatocellular Carcinoma	1	643	Stage II	T2	None	0 - No Fibrosis	No History of Primary Risk Factors	NotAvailable	8.661050466	5.139731213	7.409190061	1.864677174
TCGA-DD-A3A1	MALE	65	Hepatocellular Carcinoma	1	233	Stage IIIA	T3b	Macro	0 - No Fibrosis	No History of Primary Risk Factors	NotAvailable	82.88855091	48.24157982	2.059333005	4.038486365
TCGA-DD-A73F	FEMALE	77	Hepatocellular Carcinoma	0	1085	Stage I	T1	None	0 - No Fibrosis	No History of Primary Risk Factors	NotAvailable	128.2692524	24.96227618	2.20588942	5.306697264
TCGA-DD-AAE6	FEMALE	59	Hepatocellular Carcinoma	0	141	Stage I	T1	Micro	6 - Established Cirrhosis	NotAvailable	NotAvailable	133.6058728	80.17778918	9.669875001	5.210496211
TCGA-XR-A8TD	FEMALE	49	Hepatocellular Carcinoma	0	1030	Stage IIIB	T3	None	0 - No Fibrosis	No History of Primary Risk Factors	NotAvailable	351.0009255	45.57549305	2.056007104	3.299524025
TCGA-BC-A10S	MALE	81	Hepatocellular Carcinoma	1	1423	NotAvailable	T3	Micro	NotAvailable	Alcohol consumption	NotAvailable	16.47983223	14.93100951	5.331846785	2.04537753
TCGA-XR-A8TE	MALE	16	Hepatocellular Carcinoma	0	925	Stage IIIA	T3	None	0 - No Fibrosis	No History of Primary Risk Factors	NotAvailable	119.7188828	26.82320263	4.771268663	4.338881566
TCGA-DD-AAEB	MALE	60	Hepatocellular Carcinoma	0	478	Stage I	T1	None	NotAvailable	Alcohol consumption	NotAvailable	27.06143253	4.405713605	3.104127238	1.468034045
TCGA-LG-A6GG	FEMALE	79	Hepatocellular Carcinoma	0	387	Stage II	T2	Micro	NotAvailable	Other	NotAvailable	40.03513455	26.47285727	6.934768164	2.362605067
TCGA-BC-A10R	FEMALE	66	Hepatocellular Carcinoma	1	308	NotAvailable	T3	Micro	NotAvailable	No History of Primary Risk Factors	NotAvailable	32.65685269	34.4032877	2.960218666	1.985646012
TCGA-DD-A4NN	FEMALE	56	Hepatocellular Carcinoma	1	899	Stage I	T1	None	0 - No Fibrosis	No History of Primary Risk Factors	NotAvailable	61.24232012	17.25831047	2.898827042	2.336459512
TCGA-DD-A3A2	FEMALE	76	Hepatocellular Carcinoma	1	2131	Stage I	T1	None	0 - No Fibrosis	No History of Primary Risk Factors	NotAvailable	117.8359741	10.5066399	2.394750427	3.195960277
TCGA-DD-AAW1	MALE	55	Hepatocellular Carcinoma	0	1989	Stage IIIA	T3	Micro	6 - Established Cirrhosis	No History of Primary Risk Factors	NotAvailable	60.52690593	20.95123035	5.380180246	4.462527526
TCGA-BC-4073	MALE	73	Hepatocellular Carcinoma	0	849	Stage IIIA	T3	Macro	NotAvailable	Hemochromatosis	NotAvailable	172.2786531	70.93281062	8.906923239	9.326019521
TCGA-EP-A3RK	MALE	73	Hepatocellular Carcinoma	0	363	Stage IIIA	T3a	NotAvailable	1,2 - Portal Fibrosis	Alcohol consumption	NotAvailable	51.86710619	8.545199024	4.278850581	3.615233383
TCGA-BC-A8YO	FEMALE	66	Hepatocellular Carcinoma	0	562	Stage IIIC	T4	Macro	NotAvailable	Alcohol consumption	NotAvailable	56.15755593	16.94970941	3.372039899	5.715604484
TCGA-2Y-A9GY	FEMALE	64	Hepatocellular Carcinoma	1	757	Stage II	T2	NotAvailable	NotAvailable	No History of Primary Risk Factors	NotAvailable	45.46071016	25.49030247	9.634418593	4.328909992
TCGA-BC-A3KG	FEMALE	68	Hepatocellular Carcinoma	0	680	Stage II	T2	Micro	NotAvailable	Alcohol consumption	NotAvailable	219.7612963	15.98237237	13.27370644	6.327459563
TCGA-DD-AADR	MALE	58	Hepatocellular Carcinoma	0	2028	Stage I	T1	None	6 - Established Cirrhosis	Alcohol consumption	NotAvailable	19.60064348	15.58659509	7.174082347	3.38387216
TCGA-RC-A6M6	MALE	75	Hepatocellular Carcinoma	0	9	Stage II	T2	None	NotAvailable	Alcohol consumption	NotAvailable	54.97310336	8.934035178	17.3622309	4.223766035
TCGA-DD-AADM	MALE	58	Hepatocellular Carcinoma	1	12	Stage II	T2	None	NotAvailable	Alcohol consumption	NotAvailable	27.83579172	17.14500919	1.890987138	1.920925528
TCGA-GJ-A6C0	FEMALE	75	Hepatocellular Carcinoma	1	31	Stage II	T2	Micro	NotAvailable	No History of Primary Risk Factors	NotAvailable	158.2532109	32.65835269	3.458480949	1.838142876
TCGA-BC-A10Y	MALE	76	Hepatocellular Carcinoma	1	711	NotAvailable	T4	Micro	NotAvailable	Alcohol consumption	NotAvailable	37.11127532	5.564391013	1.031471427	2.057512206
TCGA-G3-A25S	MALE	64	Hepatocellular Carcinoma	1	416	Stage I	T1	Micro	6 - Established Cirrhosis	Alcohol consumption	NotAvailable	33.93264794	18.16174111	7.892510267	2.393192961
TCGA-WQ-AB4B	MALE	62	Hepatocellular Carcinoma	0	395	Stage II	T2	Micro	NotAvailable	Alcohol consumption	NotAvailable	14.01291192	8.431351518	3.168827702	1.534235598
TCGA-DD-AAE3	MALE	50	Hepatocellular Carcinoma	0	566	Stage I	T1	None	1,2 - Portal Fibrosis	Alcohol consumption	NotAvailable	9.712481334	5.732129892	4.766461428	0.839388954
TCGA-DD-AAE9	MALE	69	Hepatocellular Carcinoma	0	722	Stage I	T1	None	1,2 - Portal Fibrosis	Alcohol consumption	NotAvailable	32.5138205	7.647208798	11.42778625	1.850685567
TCGA-DD-AACI	MALE	69	Hepatocellular Carcinoma	0	1618	Stage II	T2	None	5 - Nodular Formation and Incomplete Cirrhos	NotAvailable	NotAvailable	6.05496454	10.17545167	2.327193589	0.984313229
TCGA-G3-A5SM	MALE	58	Hepatocellular Carcinoma	0	520	Stage II	T2	None	6 - Established Cirrhosis	Alcohol consumption	NotAvailable	44.65743735	10.86994413	3.1818537	1.649105042
TCGA-DD-AADO	MALE	55	Hepatocellular Carcinoma	0	453	Stage I	T1	Micro	NotAvailable	Alcohol consumption	NotAvailable	86.45697978	24.06875264	5.57257798	3.049488726
TCGA-BC-A112	MALE	80	Hepatocellular Carcinoma	1	153	NotAvailable	T3	None	0 - No Fibrosis	Alcohol consumption	NotAvailable	55.70864601	16.34880512	2.325365836	6.91436767
TCGA-DD-AADQ	MALE	59	Hepatocellular Carcinoma	0	436	Stage II	T2	None	NotAvailable	Alcohol consumption	NotAvailable	18.17838795	11.81655835	6.832524971	1.622059932
TCGA-DD-AAD3	MALE	43	Hepatocellular Carcinoma	0	1295	Stage I	T1	None	1,2 - Portal Fibrosis	Alcohol consumption	NotAvailable	58.9129694	19.42495075	2.90732453	1.502338749
TCGA-DD-A39Z	FEMALE	43	Hepatocellular Carcinoma	1	601	Stage II	T2	None	NotAvailable	Alcohol consumption	NotAvailable	62.21710765	34.3605601	2.574042903	3.137008652
TCGA-FV-A496	FEMALE	84	Hepatocellular Carcinoma	0	10	Stage I	T1	None	0 - No Fibrosis	No History of Primary Risk Factors	NotAvailable	323.442354	49.08917899	2.507103495	5.722974434
TCGA-2Y-A9H6	FEMALE	68	Hepatocellular Carcinoma	0	357	Stage I	T1	Macro	NotAvailable	Alcohol consumption	NotAvailable	17.78779157	20.39687671	5.505748866	1.902971851

4. HBV only

barcode	gender	Age at dia	histologica	FU OS stat	FU days	pathologic_stage	tnm.t	vascular_tumor_cell_type	fibrosis_ishak_score	hist_hepato_carc_fact	viral_hepatitis_serology	FAS	SREBF1	PPARG	ACACA
TCGA-DD	MALE	38	Hepatocel	0	1570	Stage II	T2	Micro	6 - Established Cirrhosis	Hepatitis B	NotAvailable	22.35896454	8.210454049	4.283887727	2.327359447
TCGA-DD	FEMALE	45	Hepatocel	0	555	Stage IIIA	T3a	None	3,4 - Fibrous Speta	Hepatitis B	NotAvailable	27.44049215	12.43057362	0.861039863	4.428688781
TCGA-DD	FEMALE	49	Hepatocel	0	608	Stage I	T1	None	6 - Established Cirrhosis	Hepatitis B	NotAvailable	5.011447983	4.40811326	0.845980615	0.808653462
TCGA-DD	MALE	72	Hepatocel	1	16	Stage I	T1	Micro	1,2 - Portal Fibrosis	Hepatitis B	NotAvailable	45.58343487	6.222013285	7.219150775	3.044772198
TCGA-ZP-	MALE	72	Hepatocel	0	706	NotAvailable	T1	None	3,4 - Fibrous Speta	Hepatitis B	Hepatitis B Surface Antigen	111.4998549	14.23879312	1.721384415	6.456417677
TCGA-DD	FEMALE	73	Hepatocel	0	1219	Stage I	T1	None	1,2 - Portal Fibrosis	Hepatitis B	NotAvailable	48.78757385	45.78684926	5.915565217	2.234575807
TCGA-BC-	MALE	50	Hepatocel	1	91	NotAvailable	T4	NotAvailable	NotAvailable	Hepatitis B	Hepatitis B Surface Antigen	87.94499928	18.69450873	4.811624085	5.337103686
TCGA-DD	MALE	48	Hepatocel	0	587	Stage I	T1	None	6 - Established Cirrhosis	Hepatitis B	NotAvailable	262.0518876	21.64545907	3.160424607	5.889634834
TCGA-DD	MALE	46	Hepatocel	0	183	Stage I	T1	None	6 - Established Cirrhosis	Hepatitis B	Hepatitis B Surface Antigen	94.35856415	27.4027725	12.20716007	7.735490162
TCGA-DD	MALE	55	Hepatocel	0	810	Stage I	T1	None	6 - Established Cirrhosis	Hepatitis B	NotAvailable	43.45675107	14.04087585	4.773940965	5.659115523
TCGA-G3-	MALE	58	Hepatocel	0	655	Stage I	T1	None	5 - Nodular Formation and Incomplete Cirrhosis	Hepatitis B	NotAvailable	155.4292121	6.432353775	4.536046234	4.065499824
TCGA-RC-	MALE	53	Hepatocel	0	588	Stage II	T2	None	0 - No Fibrosis	Hepatitis B	NotAvailable	41.75390313	5.684825968	5.641269466	2.145437565
TCGA-G3-	FEMALE	52	Hepatocel	1	452	Stage I	T1	None	3,4 - Fibrous Speta	Hepatitis B	NotAvailable	36.78101852	8.266749951	3.147699718	2.406178993
TCGA-DD	MALE	66	Hepatocel	0	658	Stage I	T1	Micro	1,2 - Portal Fibrosis	Hepatitis B	NotAvailable	48.88275503	35.62400519	3.899109004	3.563789701
TCGA-DD	MALE	61	Hepatocel	0	1450	Stage I	T1	None	3,4 - Fibrous Speta	Hepatitis B	NotAvailable	38.10785097	58.6561023	1.566370128	1.403780067
TCGA-QA	MALE	48	Hepatocel	0	94	Stage II	T2	Micro	0 - No Fibrosis	Hepatitis B	NotAvailable	558.5990297	9.341860448	0.735383118	7.307906855
TCGA-DD	MALE	38	Hepatocel	0	2728	Stage I	T1	None	6 - Established Cirrhosis	Hepatitis B	NotAvailable	67.83390497	18.06255302	3.459593768	2.928457054
TCGA-DD	FEMALE	43	Hepatocel	0	1085	Stage I	T1	None	NotAvailable	Hepatitis B	NotAvailable	13.16556356	11.66794234	2.302309092	1.581880434
TCGA-RC-	FEMALE	47	Hepatocel	0	640	Stage I	T1	None	6 - Established Cirrhosis	Hepatitis B	NotAvailable	28.17905612	52.23758414	1.667180482	2.776343015
TCGA-DD	MALE	68	Hepatocel	1	1622	Stage IIIA	T3	NotAvailable	3,4 - Fibrous Speta	Hepatitis B	Hepatitis B Surface Antigen	82.14236987	33.28550893	4.604862931	4.63919003
TCGA-DD	MALE	35	Hepatocel	0	2317	Stage I	T1	None	6 - Established Cirrhosis	Hepatitis B	NotAvailable	41.9977929	12.34449204	4.710399125	2.932025793
TCGA-DD	MALE	68	Hepatocel	0	2415	Stage II	T2	Micro	1,2 - Portal Fibrosis	Hepatitis B	Hepatitis B Surface Antigen	96.00189831	7.629237594	2.630774095	5.450202707
TCGA-BW	FEMALE	26	Hepatocel	0	0	Stage IV	T2	Micro	3,4 - Fibrous Speta	Hepatitis B	NotAvailable	65.60107808	35.17188812	12.33693715	4.092725003
TCGA-DD	FEMALE	69	Hepatocel	0	1562	Stage I	T1	None	NotAvailable	Hepatitis B	NotAvailable	37.80510244	8.062792877	2.241004666	1.343692835
TCGA-DD	MALE	65	Hepatocel	0	2301	Stage I	T1	None	6 - Established Cirrhosis	Hepatitis B	NotAvailable	147.2871873	21.81553581	8.340089259	9.258887475
TCGA-DD	MALE	52	Hepatocel	1	469	Stage II	T2	Micro	6 - Established Cirrhosis	Hepatitis B	NotAvailable	28.00462612	13.82738582	11.6920013	4.848896807
TCGA-DD	MALE	40	Hepatocel	1	223	Stage IV	T3a	Micro	0 - No Fibrosis	Hepatitis B	Hepatitis B Surface Antigen	15.26215757	7.181914193	1.658046016	1.648328917
TCGA-DD	MALE	69	Hepatocel	1	195	Stage II	T2	Micro	6 - Established Cirrhosis	Hepatitis B	NotAvailable	79.05129227	15.20669634	8.87366793	4.840465581
TCGA-DD	MALE	32	Hepatocel	0	1302	Stage I	T1	None	NotAvailable	Hepatitis B	NotAvailable	28.06110146	10.23873081	2.873206573	1.983358972
TCGA-DD	MALE	45	Hepatocel	0	458	Stage I	T1	Micro	NotAvailable	Hepatitis B	NotAvailable	171.6408011	63.52753909	3.610401721	6.288990917
TCGA-XR-	MALE	74	Hepatocel	1	693	Stage I	T1	None	3,4 - Fibrous Speta	Hepatitis B	NotAvailable	30.52110704	8.266115558	4.50976608	2.120107984
TCGA-DD	MALE	56	Hepatocel	0	1823	Stage I	T1	None	3,4 - Fibrous Speta	Hepatitis B	NotAvailable	33.12415257	21.21615866	4.936105949	2.527253166
TCGA-DD	MALE	52	Hepatocel	0	552	Stage I	T1	None	6 - Established Cirrhosis	Hepatitis B	NotAvailable	48.14240483	43.1189918	10.84294068	4.034345989
TCGA-DD	MALE	51	Hepatocel	0	1067	Stage II	T2	None	6 - Established Cirrhosis	Hepatitis B	NotAvailable	274.3637837	26.35411551	4.078058299	4.885526949
TCGA-DD	FEMALE	64	Hepatocel	1	115	Stage I	T1	None	NotAvailable	Hepatitis B	NotAvailable	49.5328932	19.54496625	1.492617659	0.974220846
TCGA-DD	MALE	39	Hepatocel	0	1804	Stage I	T1	None	NotAvailable	Hepatitis B	NotAvailable	408.6402122	84.09066544	2.762831373	4.96538461
TCGA-DD	FEMALE	74	Hepatocel	0	2324	Stage I	T1	Micro	6 - Established Cirrhosis	Hepatitis B	NotAvailable	31.03942867	21.90131408	5.889517769	2.412580492
TCGA-DD	MALE	50	Hepatocel	1	432	Stage II	T2	None	NotAvailable	Hepatitis B	NotAvailable	217.4578804	90.26693263	2.282000658	5.281190662
TCGA-G3-	MALE	73	Hepatocel	0	1779	Stage II	T2	None	1,2 - Portal Fibrosis	Hepatitis B	NotAvailable	10.37390723	13.31918748	1.361225827	2.63982402
TCGA-DD	MALE	48	Hepatocel	0	2752	Stage I	T1	None	6 - Established Cirrhosis	Hepatitis B	NotAvailable	105.4434865	8.820539422	3.438179681	2.09682591
TCGA-G3-	MALE	53	Hepatocel	0	780	Stage IIIA	T3a	None	6 - Established Cirrhosis	Hepatitis B	NotAvailable	43.77876416	6.955590044	1.438079436	3.153686029
TCGA-DD	MALE	59	Hepatocel	0	1567	Stage I	T1	None	NotAvailable	Hepatitis B	NotAvailable	44.31382225	33.16533441	8.36980364	2.940005689
TCGA-DD	FEMALE	68	Hepatocel	0	1049	Stage II	T2	Micro	6 - Established Cirrhosis	Hepatitis B	NotAvailable	50.77906557	19.6810922	1.56462182	3.309460907
TCGA-DD	MALE	44	Hepatocel	0	2513	Stage I	T1	None	6 - Established Cirrhosis	Hepatitis B	NotAvailable	7.744355816	10.52354718	4.690963428	1.259310537
TCGA-RC-	MALE	42	Hepatocel	0	468	Stage II	T2	Micro	3,4 - Fibrous Speta	Hepatitis B	NotAvailable	192.6492615	45.25693712	4.070914815	3.559663052
TCGA-G3-	MALE	38	Hepatocel	0	361	Stage II	T2	Micro	5 - Nodular Formation and Incomplete Cirrhosis	Hepatitis B	Hepatitis B Surface Antigen	106.8044246	44.03867044	4.878304096	5.886164397
TCGA-DD	MALE	51	Hepatocel	0	1231	Stage I	T1	None	NotAvailable	Hepatitis B	NotAvailable	275.510312	42.79741474	12.72404637	11.04603212
TCGA-DD	MALE	23	Hepatocel	1	415	Stage II	T2	None	0 - No Fibrosis	Hepatitis B	Hepatitis B Surface Antigen	44.45905908	8.632217834	7.974559241	6.730572247
TCGA-DD	MALE	51	Hepatocel	0	638	Stage I	T1	None	6 - Established Cirrhosis	Hepatitis B	NotAvailable	246.6385523	16.3357645	3.271827228	3.270711011
TCGA-DD	MALE	53	Hepatocel	1	425	Stage I	T1	Macro	NotAvailable	Hepatitis B	NotAvailable	111.115912	25.40459636	9.398126706	3.526943737
TCGA-DD	MALE	23	Hepatocel	0	1495	Stage III	T3	Macro	3,4 - Fibrous Speta	Hepatitis B	Hepatitis B Surface Antigen	123.0620211	34.93840124	6.758114407	5.958636403
TCGA-DD	FEMALE	51	Hepatocel	0	564	Stage I	T1	None	NotAvailable	Hepatitis B	NotAvailable	87.95637512	31.86566	2.632586593	5.915725821
TCGA-DD	MALE	54	Hepatocel	0	2015	Stage I	T1	None	3,4 - Fibrous Speta	Hepatitis B	NotAvailable	92.72983606	14.75119788	2.926219257	2.721700008
TCGA-G3-	FEMALE	83	Hepatocel	1	27	Stage I	T1	None	3,4 - Fibrous Speta	Hepatitis B	NotAvailable	61.13105925	15.72341021	6.603662432	3.366424235
TCGA-DD	MALE	61	Hepatocel	1	1685	Stage I	T1	None	6 - Established Cirrhosis	Hepatitis B	NotAvailable	34.70702032	15.0000639	4.658065278	1.744236543
TCGA-DD	FEMALE	55	Hepatocel	0	555	Stage I	T1	None	6 - Established Cirrhosis	Hepatitis B	NotAvailable	76.65406344	61.0823058	1.812014788	4.429060475
TCGA-DD	FEMALE	66	Hepatocel	0	1233	Stage I	T1	Macro	NotAvailable	Hepatitis B	NotAvailable	13.52638145	8.319093675	2.939794695	0.902240475
TCGA-DD	MALE	51	Hepatocel	0	347	Stage I	T1	None	6 - Established Cirrhosis	Hepatitis B	NotAvailable	56.56278175	16.3942853	2.371094789	3.152828773
TCGA-DD	FEMALE	73	Hepatocel	0	137	Stage I	T1	None	NotAvailable	Hepatitis B	NotAvailable	135.6281997	23.10309257	4.148754266	3.12617131
TCGA-DD	MALE	56	Hepatocel	0	2455	Stage II	T2	Micro	3,4 - Fibrous Speta	Hepatitis B	NotAvailable	50.7453905	21.01785196	3.344908299	2.589705549
TCGA-DD	MALE	62	Hepatocel	0	2184	Stage I	T1	None	6 - Established Cirrhosis	Hepatitis B	NotAvailable	78.03995486	7.451054094	6.56507473	2.939735613
TCGA-DD	MALE	48	Hepatocel	1	381	Stage I	T1	Micro	1,2 - Portal Fibrosis	Hepatitis B	NotAvailable	22.11687848	7.774573354	2.043352897	2.001744438
TCGA-XR-	FEMALE	43	Hepatocel	0	1339	Stage I	T1	None	6 - Established Cirrhosis	Hepatitis B	NotAvailable	65.80291304	9.998084893	2.65018227	3.319410339
TCGA-DD	MALE	72	Hepatocel	0	1531	Stage I	T1	None	6 - Established Cirrhosis	Hepatitis B	NotAvailable	64.36096458	5.46258697	2.727351907	1.829459639
TCGA-DD	MALE	70	Hepatocel	0	9	Stage I	T1	Micro	6 - Established Cirrhosis	Hepatitis B	NotAvailable	183.5277152	35.2808815	5.75003522	6.308846841
TCGA-DD	MALE	38	Hepatocel	0	1900	Stage I	T1	None	6 - Established Cirrhosis	Hepatitis B	NotAvailable	62.93784594	20.849151	7.922898882	3.27377346
TCGA-DD	MALE	66	Hepatocel	0	672	Stage IIIA	T3a	None	NotAvailable	Hepatitis B	NotAvailable	84.7011449	25.72715246	14.69678632	8.006447592
TCGA-DD	MALE	46	Hepatocel	0	2202	Stage II	T2	Micro	3,4 - Fibrous Speta	Hepatitis B	NotAvailable	21.97164127	7.572154625	7.126979588	2.364329774
TCGA-UB-	MALE	51	Hepatocel	0	486	Stage I	T1	None	1,2 - Portal Fibrosis	Hepatitis B	Hepatitis B Surface Antigen	35.45277405	17.03431863	5.983884043	2.489490403
TCGA-DD	MALE	40	Hepatocel	0	1876	Stage I	T1	None	6 - Established Cirrhosis	Hepatitis B	NotAvailable	7.622869565	11.24756112	0.842706871	1.845958874
TCGA-DD	MALE	51	Hepatocel	0	1242	Stage I	T1	None	NotAvailable	Hepatitis B	NotAvailable	215.9735992	40.75177954	13.90062175	5.229352455
TCGA-G3-	MALE	61	Hepatocel	0	585	Stage I	T1	None	6 - Established Cirrhosis	Hepatitis B	NotAvailable	55.22751152	6.077709657	2.291661747	2.604285439
TCGA-G3-	FEMALE	63	Hepatocel	0	1636	Stage I	T1	None	0 - No Fibrosis	Hepatitis B	NotAvailable	192.9447089	8.457721204	1.37537594	3.498760574
TCGA-ED-	MALE	61	Hepatocel	0	6	Stage II	T2	Micro	NotAvailable	No History of Primary Risk Factors	Hepatitis B Surface Antigen	46.22746544	36.98023366	1.355615273	4.014428525
TCGA-ED-	MALE	58	Hepatocel	0	819	Stage II	T2	Micro	NotAvailable	No History of Primary Risk Factors	Hepatitis B Surface Antigen	45.64009449	38.3624941	3.235690722	2.354498647
TCGA-DD	FEMALE	78	Hepatocel	1	1694	Stage I	T1	None	0 - No Fibrosis	No History of Primary Risk Factors	Hepatitis B Surface Antigen	42.31029664	14.22938512	2.459248121	2.455735285
TCGA-ED-	FEMALE	48	Hepatocel	0	6	Stage II	T2	Micro	NotAvailable	No History of Primary Risk Factors	Hepatitis B Surface Antigen	77.28547677	23.27155821	1.170861519	13.37050353
TCGA-ED-	FEMALE	51	Hepatocel	1	296	Stage IIIA	T3a	Micro	NotAvailable	No History of Primary Risk Factors	Hepatitis B Surface Antigen	44.96633728	61.6022001	7.372068179	4.023831366
TCGA-DD	MALE	67	Hepatocel	1	171	Stage I	T1	None	0 - No Fibrosis	No History of Primary Risk Factors	Hepatitis B Surface Antigen	166.4086798	33.68100221	13.24174901	6.59828502
TCGA-DD	FEMALE	73	Hepatocel	0	3478	Stage I	T1	None	0 - No Fibrosis	No History of Primary Risk Factors	Hepatitis B Surface Antigen	39.65254989	41.22614334	3.323730246	2.370609157
TCGA-DD	FEMALE	67	Hepatocel	0	1008	Stage IIIC	T2	NotAvailable	5 - Nodular Formation and Incomplete Cirrhosis	No History of Primary Risk Factors	Hepatitis B Surface Antigen	8.255566614	8.931317612	0.559144223	3.478317229
TCGA-ED-	FEMALE	53	Hepatocel	0	400	Stage II	T2	Micro	NotAvailable	No History of Primary Risk Factors	Hepatitis B Surface Antigen	35.64054918	10.263986	3.223364835	2.790551453
TCGA-ED-	FEMALE	60	Hepatocel	0	854	Stage II	T2	Micro	NotAvailable	No History of Primary Risk Factors	Hepatitis B Surface Antigen	51.8344541	18.57790416	5.128315702	3.42355759
TCGA-DD	FEMALE	57	Hepatocel	1	394	Stage I	T1	None	3,4 - Fibrous Speta	No History of Primary Risk Factors	Hepatitis B Surface Antigen	29.33255981	12.56949509	23.12914344	3.723312112
TCGA-DD	MALE	32	Hepatocel	0	3308	Stage I	T1	None	0 - No Fibrosis	No History of Primary Risk Factors	Hepatitis B Surface Antigen	47.08663945	13.8758756	2.932378518	2.103589802
TCGA-DD	MALE	75	Hepatocel	1	11	Stage II	T2	None	0 - No Fibrosis	No History of Primary Risk Factors	Hepatitis B Surface Antigen	59.41352446	12.69470208	2.54449943	2.511960073
TCGA-G3-	FEMALE	53	Hepatocel	1	65	Stage IIIA	T3a	Micro	0 - No Fibrosis	No History of Primary Risk Factors	Hepatitis B Surface Antigen	77.91199587	25.60160645	8.185790793	2.922284136
TCGA-DD	FEMALE	65	Hepatocel	0	701	Stage IIIA	T3a	None	0 - No Fibrosis	No History of Primary Risk Factors	Hepatitis B Surface Antigen	237.5359837	7.586085991	3.787100048	5.874619246
TCGA-ED-	FEMALE	20	Hepatocel	0	390	Stage II	T2	Micro	NotAvailable	No History of Primary Risk Factors	Hepatitis B Surface Antigen	22.87971457	25.0985338	4.231924005	1.806584609
TCGA-DD	FEMALE	80	Hepatocel	1	1210	Stage IIIA	T3	None	0 - No Fibrosis	No History of Primary Risk Factors	Hepatitis B Surface Antigen	143.4113918	6.356812583	1.033035009	2.438914152
TCGA-DD	FEMALE	71	Hepatocel	1	1005	Stage IIIC	T1	None	0 - No Fibrosis	No History of Primary Risk Factors	Hepatitis B Surface Antigen	196.6064199	65.64743725	19.57166118	10.64990904
TCGA-DD	FEMALE	72	Hepatocel	1	3258	Stage II	T2	Micro	0 - No Fibrosis	No History of Primary Risk Factors	Hepatitis B Surface Antigen	5.82791927	5.014243722	64.39303131	0.940745129

5. HCV only

barcode	gender	Age at diagnosis	histological type	FU OS status	FU days	pathologic stage	tnm.t	vascular tumor cell type	fibrosis ishak score	hist hepato carc fact	viral hepatitis serology	FAS	SREBF1	PPARG	ACACA
TCGA-DD-A1EE	MALE	73	Hepatocellular Carcinoma	1	349	Stage IIIA	T3	None	6 - Established Cirrhosis	Hepatitis C	Hepatitis C Virus RNA	171.2477357	31.56232743	2.818505458	7.212838105
TCGA-G3-A5SK	MALE	58	Hepatocellular Carcinoma	0	744	Stage I	T1	NotAvailable	6 - Established Cirrhosis	Hepatitis C	NotAvailable	28.76042765	20.27476332	1.379266397	0.699957383
TCGA-XR-A8TG	MALE	58	Hepatocellular Carcinoma	0	898	Stage I	T1	None	6 - Established Cirrhosis	Hepatitis C	NotAvailable	29.71127635	18.91190129	5.289780207	3.04193043
TCGA-K7-A5RF	MALE	64	Hepatocellular Carcinoma	0	631	Stage I	T1	NotAvailable	NotAvailable	Hepatitis C	Hepatitis C Virus RNA	19.91464746	14.25842509	2.325291372	0.924920034
TCGA-RC-A7SF	MALE	66	Hepatocellular Carcinoma	0	579	Stage I	T1	None	3,4 - Fibrous Speta	Hepatitis C	NotAvailable	287.3975975	70.03215383	1.871034719	3.449339248
TCGA-3K-AAZ8	MALE	65	Hepatocellular Carcinoma	0	396	Stage IIIB	T3b	NotAvailable	5 - Nodular Formation and Incomplete Cirrhosis	Hepatitis C	NotAvailable	42.17715464	9.989275835	4.237541694	2.892015052
TCGA-MI-A75I	MALE	61	Hepatocellular Carcinoma	0	630	NotAvailable	T2	NotAvailable	NotAvailable	Hepatitis C	NotAvailable	155.1220033	14.44159457	4.951347417	4.785145094
TCGA-EP-A2KB	FEMALE	46	Hepatocellular Carcinoma	1	596	Stage I	T1	None	NotAvailable	Hepatitis C	NotAvailable	27.27083342	10.5315698	15.35883156	3.258766832
TCGA-G3-A3CG	MALE	80	Hepatocellular Carcinoma	0	673	Stage I	T1	Micro	6 - Established Cirrhosis	Hepatitis C	NotAvailable	261.6235756	102.8241115	2.40491564	5.145438345
TCGA-G3-A25T	FEMALE	45	Hepatocellular Carcinoma	0	1553	Stage IIIA	T3	None	0 - No Fibrosis	Hepatitis C	NotAvailable	9.840705427	15.43974363	2.430765624	1.531885334
TCGA-DD-A73D	FEMALE	68	Hepatocellular Carcinoma	0	693	Stage II	T2	Micro	6 - Established Cirrhosis	Hepatitis C	Hepatitis C Virus RNA	36.62111796	3.321474261	4.871095444	3.766990665
TCGA-FV-A495	FEMALE	51	Hepatocellular Carcinoma	0	1	Stage II	T2	None	3,4 - Fibrous Speta	Hepatitis C	NotAvailable	69.45027742	23.13687422	2.920166597	1.963071364
TCGA-DD-A4NF	MALE	72	Hepatocellular Carcinoma	0	942	Stage I	T1	None	6 - Established Cirrhosis	Hepatitis C	Hepatitis C Virus RNA	38.53690401	11.80172355	4.843333056	5.053814754
TCGA-YA-A8S7	MALE	68	Hepatocellular Carcinoma	1	412	Stage IIIA	T3a	None	NotAvailable	Hepatitis C	NotAvailable	22.81635384	13.87341997	7.75784656	2.827828214
TCGA-G3-A3CJ	MALE	52	Hepatocellular Carcinoma	0	594	Stage II	T2	Micro	5 - Nodular Formation and Incomplete Cirrhosis	Hepatitis C	NotAvailable	54.49914199	12.26989785	2.895612104	2.727803972
TCGA-ZP-A9D2	MALE	51	Hepatocellular Carcinoma	1	765	NotAvailable	T2	Micro	1,2 - Portal Fibrosis	Hepatitis C	Hepatitis C Virus RNA	23.37276315	4.292433642	1.029497401	6.084070048
TCGA-DD-AACF	MALE	68	Hepatocellular Carcinoma	1	365	Stage I	T1	None	6 - Established Cirrhosis	Hepatitis C	NotAvailable	40.5850971	4.093340471	0.7235322	1.233982504
TCGA-FV-A4ZO	MALE	52	Hepatocellular Carcinoma	0	12	Stage I	T1	None	NotAvailable	Hepatitis C	Hepatitis C Virus RNA	110.136577	30.56297335	12.85758501	5.954798723
TCGA-G3-A7M6	FEMALE	60	Hepatocellular Carcinoma	0	632	Stage I	T1	None	1,2 - Portal Fibrosis	Hepatitis C	NotAvailable	33.60391377	12.9661357	8.503783012	5.721688291
TCGA-MI-A75H	MALE	77	Hepatocellular Carcinoma	0	747	NotAvailable	NotAvailable	Micro	6 - Established Cirrhosis	Hepatitis C	HCV Genotype	33.73881428	6.614166144	3.923277482	1.59080586
TCGA-BW-A5NO	MALE	50	Hepatocellular Carcinoma	0	20	Stage IIIA	T3a	None	3,4 - Fibrous Speta	Hepatitis C	NotAvailable	118.4495983	16.19240409	5.97655406	5.780826008
TCGA-DD-AACL	FEMALE	66	Hepatocellular Carcinoma	1	107	Stage I	T1	None	NotAvailable	Hepatitis C	NotAvailable	122.3638785	21.36575212	10.72592292	5.616332916
TCGA-EP-A12J	MALE	62	Hepatocellular Carcinoma	0	570	Stage I	T1	None	NotAvailable	Hepatitis C	NotAvailable	53.18848029	25.55438145	8.296543295	4.453507434
TCGA-DD-A114	MALE	42	Hepatocellular Carcinoma	1	1149	Stage II	T2	Micro	5 - Nodular Formation and Incomplete Cirrhosis	Hepatitis C	Hepatitis C Virus RNA	102.5492797	16.0029413	2.464405697	3.608413625
TCGA-FV-A23B	FEMALE	70	Hepatocellular Carcinoma	1	1852	Stage II	T2	NotAvailable	NotAvailable	Hepatitis C	NotAvailable	50.68394213	20.70666467	10.92522691	5.702100166
TCGA-DD-A4NO	MALE	65	Hepatocellular Carcinoma	0	2245	Stage I	T1	None	0 - No Fibrosis	Hepatitis C	Hepatitis C Virus RNA	105.8240857	8.562818463	2.679466137	4.017252919
TCGA-ES-A2HT	MALE	54	Hepatocellular Carcinoma	1	438	Stage I	T1	None	0 - No Fibrosis	Hepatitis C	NotAvailable	29.41584572	29.86534411	0.601138464	1.684021587
TCGA-RG-A7D4	MALE	69	Hepatocellular Carcinoma	0	1098	Stage II	T2	NotAvailable	NotAvailable	Hepatitis C	Hepatitis C Antibody	58.4577994	8.222505363	10.89771786	7.710954959
TCGA-RC-A7SK	MALE	59	Hepatocellular Carcinoma	0	472	Stage I	T1	Micro	1,2 - Portal Fibrosis	Hepatitis C	NotAvailable	38.27241917	14.12737358	3.021037412	2.777680829
TCGA-EP-A2KC	MALE	62	Hepatocellular Carcinoma	1	19	Stage I	T1	None	NotAvailable	Hepatitis C	NotAvailable	99.98486277	8.88424776	4.733373142	6.533093477
TCGA-G3-A5SI	MALE	44	Hepatocellular Carcinoma	1	768	Stage II	T2	None	6 - Established Cirrhosis	Hepatitis C	NotAvailable	35.23821592	10.51856337	0.449133161	1.210112035
TCGA-CC-A8HV	FEMALE	51	Hepatocellular Carcinoma	1	279	Stage II	T2	NotAvailable	NotAvailable	No History of Primary Risk Factors	Hepatitis C Antibody	155.878269	25.50374357	6.510655293	5.990620782
TCGA-2Y-A9GU	FEMALE	55	Hepatocellular Carcinoma	0	1939	Stage I	T1	NotAvailable	NotAvailable	No History of Primary Risk Factors	Hepatitis C Antibody	123.2390458	41.29981819	2.842026877	2.910287395
TCGA-CC-A8HU	FEMALE	39	Hepatocellular Carcinoma	1	344	Stage IIIA	T3	NotAvailable	NotAvailable	No History of Primary Risk Factors	Hepatitis C Antibody	31.01665021	5.959179031	9.742199212	2.486943135
TCGA-ZS-A9CG	MALE	55	Hepatocellular Carcinoma	0	341	Stage II	T2	Micro	0 - No Fibrosis	NotAvailable	Hepatitis C Antibody	36.07056351	4.940350321	2.65109278	1.94975614
TCGA-FV-A3R2	MALE	75	Hepatocellular Carcinoma	1	194	Stage I	T1	NotAvailable	NotAvailable	No History of Primary Risk Factors	Hepatitis C Antibody	60.63167256	13.09210556	7.827583761	6.430484664
TCGA-CC-5261	MALE	44	Hepatocellular Carcinoma	1	97	Stage II	T2	None	NotAvailable	NotAvailable	Hepatitis C Antibody	61.75398231	14.65813544	2.322933777	4.668615557
TCGA-2Y-A9H7	FEMALE	81	Hepatocellular Carcinoma	0	1168	Stage I	T1	None	3,4 - Fibrous Speta	No History of Primary Risk Factors	Hepatitis C Antibody	74.78375467	41.39569846	7.671169932	5.583750946
TCGA-5R-AA1D	FEMALE	17	Hepatocellular Carcinoma	0	449	Stage IIIA	T3a	Micro	0 - No Fibrosis	No History of Primary Risk Factors	Hepatitis C Antibody	9.008337356	6.456495899	3.619471077	1.423569143
TCGA-CC-A123	FEMALE	24	Hepatocellular Carcinoma	0	219	Stage IIIA	T3	None	NotAvailable	NotAvailable	Hepatitis C Antibody	41.57970014	8.059349312	11.49412111	4.017051535
TCGA-CC-5263	MALE	35	Hepatocellular Carcinoma	1	129	Stage IIIA	T3	None	NotAvailable	No History of Primary Risk Factors	Hepatitis C Antibody	78.25763913	20.37243958	4.156819839	4.864728773
TCGA-ZS-A9CD	MALE	73	Hepatocellular Carcinoma	1	1386	Stage II	T2	None	5 - Nodular Formation and Incomplete Cirrhosis	NotAvailable	Hepatitis C Antibody	57.68075079	20.24077171	2.620381115	2.046700612
TCGA-WX-AA46	MALE	61	Hepatocellular Carcinoma	0	756	Stage II	T2	Macro	0 - No Fibrosis	No History of Primary Risk Factors	Hepatitis C Antibody	44.56836968	8.026018694	2.317561233	1.531956756
TCGA-5R-AA1C	MALE	57	Hepatocellular Carcinoma	0	520	Stage II	T2	Micro	1,2 - Portal Fibrosis	No History of Primary Risk Factors	Hepatitis C Antibody	13.86386392	4.106873862	5.85559917	2.737206407
TCGA-ED-A82E	FEMALE	60	Hepatocellular Carcinoma	0	408	Stage IIIA	T3a	Micro	NotAvailable	No History of Primary Risk Factors	Hepatitis C Antibody	11.07098885	4.943530191	0.579661454	1.481110803
TCGA-CC-A3MB	MALE	36	Hepatocellular Carcinoma	1	315	Stage IIIA	T3	NotAvailable	NotAvailable	No History of Primary Risk Factors	Hepatitis C Antibody	103.513112	65.70120654	6.308582346	7.871580521
TCGA-2Y-A9GZ	FEMALE	82	Hepatocellular Carcinoma	1	848	Stage II	T2	NotAvailable	NotAvailable	No History of Primary Risk Factors	Hepatitis C Antibody	153.7925495	2.586303624	5.29106502	1.653412624
TCGA-WX-AA47	FEMALE	33	Hepatocellular Carcinoma	1	556	Stage IIIA	T3a	Macro	0 - No Fibrosis	No History of Primary Risk Factors	Hepatitis C Antibody	77.82479241	8.521244602	1.749905076	1.623915939
TCGA-CC-5260	FEMALE	61	Hepatocellular Carcinoma	1	87	Stage IIIC	T4	None	NotAvailable	NotAvailable	Hepatitis C Antibody	31.59466464	22.69848223	4.545498608	5.288087613
TCGA-WJ-A86L	FEMALE	68	Hepatocellular Carcinoma	0	345	Stage I	T1	None	0 - No Fibrosis	No History of Primary Risk Factors	Hepatitis C Antibody	100.0201622	26.37742503	0.303370104	7.636300942
TCGA-ED-A8O5	FEMALE	59	Hepatocellular Carcinoma	0	406	Stage IIIA	T3a	Micro	NotAvailable	No History of Primary Risk Factors	Hepatitis C Antibody	49.29493513	24.37789971	9.861459031	1.685658837
TCGA-UB-A7MB	MALE	24	Hepatocellular Carcinoma	0	601	Stage II	T2	Micro	0 - No Fibrosis	No History of Primary Risk Factors	Hepatitis C Antibody	333.3578943	18.95175337	4.751915422	11.31364229
TCGA-CC-A8HS	MALE	18	Hepatocellular Carcinoma	1	300	Stage IIIC	T3	NotAvailable	NotAvailable	No History of Primary Risk Factors	Hepatitis C Antibody	63.29733083	44.74855523	5.26277986	5.812341338
TCGA-CC-A1HT	MALE	50	Hepatocellular Carcinoma	1	101	Stage IIIA	T3	NotAvailable	NotAvailable	No History of Primary Risk Factors	Hepatitis C Antibody	32.62391736	13.72724962	3.576905346	7.416743244
TCGA-ZS-A9CE	FEMALE	79	Hepatocellular Carcinoma	0	1241	Stage II	T2	Micro	0 - No Fibrosis	NotAvailable	Hepatitis C Antibody	134.6956032	7.978296035	0.320795758	1.959726155
TCGA-CC-5264	MALE	71	Hepatocellular Carcinoma	1	102	Stage IIIA	T3	None	NotAvailable	NotAvailable	Hepatitis C Antibody	63.66102835	32.90913141	5.974803858	6.694134318
TCGA-CC-5262	MALE	67	Hepatocellular Carcinoma	1	103	Stage IIIC	T4	NotAvailable	NotAvailable	No History of Primary Risk Factors	Hepatitis C Antibody	164.6504411	33.37674152	5.158298356	4.132235679
TCGA-ED-A7XO	MALE	29	Hepatocellular Carcinoma	0	427	Stage IIIA	T3a	Micro	NotAvailable	No History of Primary Risk Factors	Hepatitis C Antibody	29.86465449	6.937619317	1.862672971	1.395479541
TCGA-5R-AAAM	FEMALE	65	Hepatocellular Carcinoma	1	46	Stage II	T2	Micro	6 - Established Cirrhosis	No History of Primary Risk Factors	Hepatitis C Antibody	40.78570908	11.09734557	3.397863446	2.87683988
TCGA-CC-5258	MALE	48	Hepatocellular Carcinoma	1	129	Stage II	T2	None	NotAvailable	NotAvailable	Hepatitis C Antibody	138.1099645	20.85487555	14.05592295	7.458566297
TCGA-DD-A4NE	FEMALE	75	Hepatocellular Carcinoma	1	660	Stage IIIA	T3a	None	0 - No Fibrosis	No History of Primary Risk Factors	Hepatitis C Virus RNA	137.018847	12.6953084	1.051236579	4.482108729
TCGA-CC-5259	FEMALE	60	Hepatocellular Carcinoma	0	250	Stage IIIC	T4	NotAvailable	NotAvailable	No History of Primary Risk Factors	Hepatitis C Antibody	16.35628616	25.18456077	8.897492042	1.309887486
TCGA-HP-A5MZ	MALE	78	Hepatocellular Carcinoma	1	91	Stage I	T1	None	NotAvailable	No History of Primary Risk Factors	Hepatitis C Antibody	66.34265366	20.30384517	2.421898438	2.684095033
TCGA-ED-A8O6	FEMALE	50	Hepatocellular Carcinoma	1	56	Stage IIIA	T3a	Micro	NotAvailable	No History of Primary Risk Factors	Hepatitis C Antibody	125.0288171	36.57743402	9.730002951	7.06355762
