# Supplementary material for: De novo lipogenesis is elicited dramatically in human hepatocellular carcinoma especially in hepatitis C virus‐induced hepatocellular carcinoma
Source: MedComm (2020). 2020 Jul 9;1(2):178–87. doi: 10.1002/mco2.15 (PMC8491216; doi:10.1002/mco2.15)
Supplement: Supplementary file 6 — Table S5 [file MCO2-1-178-s001.doc]

| **Table S5. Pearson Correlation of lipogenic genes (n=361)** | | | | |
| --- | --- | --- | --- | --- |
|  | FAS | ACC | PPARγ | SREBP1c |
| FAS | 1 | 0.56** | 0.07 | 0.38** |
| ACC | 0.56** | 1 | 0.24** | 0.29** |
| PPARγ | 0.07 | 0.24** | 1 | 0.10 |
| SREBP1c | 0.38** | 0.29** | 0.10 | 1 |
|  |  |  |  |  |

**. Correlation is significant at the 0.01 level (2-tailed).
